# ROP: dumpster diving in RNA-sequencing to find the source of 1 trillion reads across diverse adult human tissues

**DOI:** 10.1186/s13059-018-1403-7

**Published:** 2018-02-15

**Authors:** Serghei Mangul, Harry Taegyun Yang, Nicolas Strauli, Franziska Gruhl, Hagit T. Porath, Kevin Hsieh, Linus Chen, Timothy Daley, Stephanie Christenson, Agata Wesolowska-Andersen, Roberto Spreafico, Cydney Rios, Celeste Eng, Andrew D. Smith, Ryan D. Hernandez, Roel A. Ophoff, Jose Rodriguez Santana, Erez Y. Levanon, Prescott G. Woodruff, Esteban Burchard, Max A. Seibold, Sagiv Shifman, Eleazar Eskin, Noah Zaitlen

**Affiliations:** 1 Department of Computer Science, University of California, Los Angeles, CA USA; 2 Institute for Quantitative and Computational Biosciences, University of California, Los Angeles, CA USA; 3 Biomedical Sciences Graduate Program, University of California, San Francisco, CA USA; 4 Center for Integrative Genomics, University of Lausanne, Lausanne, Switzerland; 5 SIB Swiss Institute of Bioinformatics, Lausanne, Switzerland; 6 The Mina and Everard Goodman Faculty of Life Sciences, Bar-Ilan University, Ramat-Gan, Israel; 7 Department of Bioengineering, University of California, Los Angeles, CA USA; 8 Molecular and Computational Biology, Department of Biological Sciences, University of Southern California, Los Angeles, CA USA; 9 Division of Pulmonary, Critical Care, Sleep and Allergy, Department of Medicine, and Cardiovascular Research Institute, University of California, San Francisco, CA USA; 10 Center for Genes, Environment, and Health, National Jewish Health, Denver, CO USA; 11 Department of Medicine, University of California, San Francisco, CA USA; 12 Department of Bioengineering and Therapeutic Sciences, University of California, San Francisco, CA USA; 13 Institute for Quantitative Biosciences, University of California, San Francisco, CA USA; 14 Institute for Human Genetics, University of California, San Francisco, San Francisco, CA USA; 15 Center for Neurobehavioral Genetics, Semel Institute for Neuroscience and Human Behavior, University California, Los Angeles, CA USA; 16 Department of Human Genetics, University of California, Los Angeles, CA USA; 17 Department of Psychiatry, Brain Center Rudolf Magnus, University Medical Center Utrecht, Utrecht, The Netherlands; 18 Centro de Neumología Pediátrica, San Juan, Puerto Rico; 19 Department of Pediatrics, National Jewish Health, Denver, CO USA; 20 University of Colorado School of Medicine, Denver, CO USA; 21 Department of Genetics, The Institute of Life Sciences, The Hebrew University of Jerusalem, Jerusalem, Israel; 22 Schools of Pharmacy and Medicine, Department of Bioengineering and Therapeutic Sciences, University of California, San Francisco, CA USA

## Abstract

High-throughput RNA-sequencing (RNA-seq) technologies provide an unprecedented opportunity to explore the individual transcriptome. Unmapped reads are a large and often overlooked output of standard RNA-seq analyses. Here, we present Read Origin Protocol (ROP), a tool for discovering the source of all reads originating from complex RNA molecules. We apply ROP to samples across 2630 individuals from 54 diverse human tissues. Our approach can account for 99.9% of 1 trillion reads of various read length. Additionally, we use ROP to investigate the functional mechanisms underlying connections between the immune system, microbiome, and disease. ROP is freely available at https://github.com/smangul1/rop/wiki.

**Electronic supplementary material:**

The online version of this article (10.1186/s13059-018-1403-7) contains supplementary material, which is available to authorized users.

## Background

Advances in RNA-sequencing (RNA-seq) technology have provided an unprecedented opportunity to explore gene expression across individuals, tissues, and environments [1–3] by efficiently profiling the RNA sequences present in a sample of interest [4]. RNA-seq experiments currently produce tens of millions of short read subsequences sampled from the complete set of RNA transcripts that are provided to the sequencing platform. An increasing number of bioinformatic protocols are being developed to analyze reads in order to annotate and quantify the sample’s transcriptome [5–7]. When a reference genome sequence or, preferably, a transcriptome of the sample is available, mapping-based RNA-seq analysis protocols align the RNA-seq reads to the reference sequences, identify novel transcripts, and quantify the abundance of expressed transcripts.

Unmapped reads, the reads that fail to map to the human reference, are a large and often overlooked output of standard RNA-seq analyses. Even in carefully executed experiments, the unmapped reads can comprise a substantial fraction of the complete set of reads produced; for example, approximately 9–20% of reads remain unmapped in recent large human RNA-seq projects [8–10]. Unmapped reads can arise due to technical sequencing artifacts that were produced by low quality and error prone copies of the nascent RNA sequence being sampled [11]. A recent study by Baruzzo et al. [12] suggests that at least 10% of the reads simulated from human references remain unmapped across 14 contemporary state-of-the art RNA aligners. This rate may be due to shortcomings of the aligner’s efficient yet heuristic algorithms [13]. Reads can also remain unmapped due to unknown transcripts [14], recombined B and T cell receptor sequences [15, 16], A-to-G mismatches from A-to-I RNA editing [17], trans-splicing [18], gene fusion [19], circular RNAs [20], and the presence of non-host RNA sequences [21] (e.g. bacterial, fungal, and viral organisms). Unmapped reads represent a rich resource for the study of B and T cell receptor repertoires and the human microbiome system—without incurring the expense of additional targeted sequencing. Studies of B and T cell repertoires produce results key to understanding the response of adaptive immunity during health and disease.

In this work, we report the development of a comprehensive method that can characterize the origin of unmapped reads obtained by RNA-seq experiments. Analyzing unmapped reads can inform future development of read mapping methods, provide access to additional biological information, and resolve the irksome puzzle of the origin of unmapped reads. We developed the Read Origin Protocol (ROP), a multi-step approach that leverages accurate alignment methods for both host and microbial sequences. The ROP tool contains a combination of novel algorithms and existing tools focused on specific categories of unmapped reads [15, 21–24]. The comprehensive analytic nature of the ROP tool prevents biases that can otherwise arise when using standard targeted analyses. ROP offers a flexible interface to customize the computational tools used in the protocol. Instructions on how to customize the tools are provided as part of the ROP tutorial.

## Results and Discussion

### ROP: a computational protocol to explain unmapped reads in RNA-seq

**Fig. 1:**
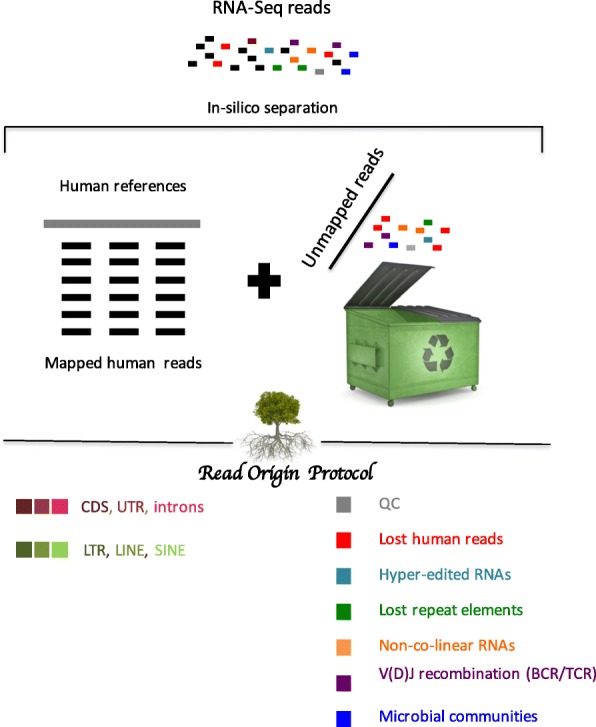
*Schematic* of the ROP. Human reads are identified by mapping all reads onto the reference sequences using a standard high-throughput mapping algorithm. ROP protocol categorizes mapped reads into categories of genomic (*red colors*) and repetitive (*green colors*) reads. Unmapped reads that fail to map are extracted and further filtered to exclude low-quality reads, low-complexity reads, and reads from ribosomal DNA (rDNA) (*grey color*). ROP protocol is able to identify unmapped reads aligned to human references with use of a more sensitive alignment tool (lost human reads: *red color*), unmapped reads aligned to human references with excessive (“hyper”) editing (hyper-edited RNAs: *cyan color*), unmapped reads aligned to the repeat sequences (lost repeat elements: *green color*), unmapped reads spanning sequences from distant loci (non-co-linear: *orange color*), unmapped reads spanning antigen receptor gene rearrangement in the variable domain (V(D)J recombination of B cell receptor and T cell receptor: *violet color*), and unmapped reads aligned to the microbial reference genomes and marker genes (microbial reads: *blue color*)

Mapping-based RNA-seq analysis protocols overlook reads that fail to map onto the human reference sequences (i.e. unmapped reads). We designed a ROP that identifies the origin of both mapped and unmapped reads (Fig. 1). The protocol first identifies human reads by using a standard high-throughput mapping algorithm to map them onto a reference genome and transcriptome [25]. We used TopHat v. 2.0.12 with ENSEMBL GRCh37 transcriptome and hg19 build, but many other mapping tools are available and have recently been reviewed [12]. After alignment, reads are grouped into genomic (e.g. CDS, UTRs, introns) and repetitive (e.g. SINEs, LINEs, LTRs) categories. The rest of the ROP protocol characterizes the remaining unmapped reads, which failed to map to the human reference sequences.

The ROP protocol effectively processes the unmapped reads in seven steps. The pairing information of the unmapped reads is disregarded and each read from the pair is counted separately. First, we apply a quality control step to exclude low-quality reads, low-complexity reads, and reads that match ribosomal DNA (rDNA) complete repeating unit among the unmapped reads (FASTQC [26], SEQCLEAN [https://sourceforge.net/projects/seqclean/]). Next, we employ Megablast [27], a more sensitive alignment method, to search for human reads missed due to heuristics implemented for computational speed in conventional aligners and reads with additional mismatches. These reads typically include those with mismatches and short gaps relative to the reference set, but they can also include perfectly matched reads. Hyper-editing pipelines recognize reads with excessive (“hyper”) editing, which are usually rejected by standard alignment methods due to many A-to-G mismatches [17]. We use a database of repeat sequences to identify lost repeat reads among the unmapped reads. Megablast, and similar sensitive alignment methods, are not designed to identify “non-co-linear” (NCL) RNA [22] reads from circular RNAs (circRNAs), gene fusions, and trans-splicing events, which combine a sequence from distant elements. Similarly, reads from B cell receptor (BCR) and T cell receptor (TCR) loci, which are subject to recombination and somatic hyper-mutation (SHM), require specifically designed methods. For this case, we use IgBlast [28]. The remaining reads that did not map to any known human sequence are potentially microbial in origin. We use microbial genomes and phylogenetic marker genes to identify microbial reads and assign them to corresponding taxa [29]. Microbial reads can be introduced by contamination or natural microbiome content in the sample, such as viral, bacterial, fungi, or other microbial species [30].

Taken together, ROP considers six classes of unmapped reads: (1) lost human reads; (2) hyper-edited reads; (3) lost repeat elements; (4) reads from NCL RNAs; (5) reads from the recombination of BCR and TCR segments (i.e. variable (diversity) joining [V(D)J] recombination); and (6) microbial reads. Previously proposed individual methods do examine some of these classes [15, 21–24]. However, we find that performing a sequential analysis, in the order described above, is critical for minimizing misclassification of reads due to homologous sequences between the different classes. Given short read length, a read may be mapped to multiple ROP categories with the same score. For example, viral genomes contain regions homologous to human sequences (viral DNA present in human genome) [31]. This may cause human reads to be mapped onto a viral genome. We therefore filter human reads before mapping reads onto viral genomes in order to be maximally conservative when assigning reads to microbial species. Furthermore, as shown in the “Results” section below, only a comprehensive analysis allows comparison across these classes. Complete details of ROP, including all parameters and thresholds used, are provided in the Additional file 1: Supplementary Methods.

#### Validation of accuracy of ROP’s read assignments

To demonstrate the accuracy of ROP’s read assignment, we simulated RNA-seq data as a mixture of transcriptomic, repeat, immune, and microbial reads (Additional file 1: Supplementary Methods). We first map the RNA-seq reads using TopHat2 (v2.1.1) aligner. TopHat2 was able to map 75.1% of transcriptomic reads. In addition to transcriptomic reads, it mapped 59.9% of repeat reads and 80.7% of immune reads (Additional file 2: Table S1a). We consider categorizing repeat and immune reads as human reads by TopHat2 or lost human reads by ROP as correct assignment, due to the presence of repeat sequences and immune genes in the human genome. Running ROP on unmapped reads identified additional 23.4% of transcriptomic reads; additional 39.2% of repeat reads; additional 12.3% of immune reads; and 100% of microbial reads. Altering the order of steps executed by ROP analysis resulted in 3.0% of repeat reads and 0.01% of transcriptomic reads to be reclassified as microbial (Additional file 2: Table S1b–d). Immune reads spanning V(D)J recombinations may not sufficiently overlap V and J genes for a reliable identification and 7.7% of those reads were missed by the ROP protocol.

In additional to simulated data, we have used TCRB-seq data prepared from three samples of kidney renal clear cell carcinoma (KIRC) by Li et al. [32] to demonstrate the assignment accuracy of immune reads. We downloaded matching RNA-seq samples from the TCGA portal. In total, we obtained 301 million 2 × 50-bp reads from three RNA-seq samples. We considered the recombinations of V and J genes obtained from TCRB-seq as the total immune repertoire. On average, ROP is able to capture 4.3% of total immune repertoire. All T cell receptor recombinations detected by ROP were confirmed by TCRB-seq (Additional file 2: Table S2). Complete details of simulated and real data, including all parameters and reference databases used, are provided in Additional file 1: Supplementary Methods, and the raw reads are available at https://smangul1.github.io/recycle.RNA.seq/.

### The ROP protocol is able to account for 99.9% of all reads

To test ROP, we applied it to 1 trillion RNA-seq reads across 54 tissues from 2630 individuals. The data were combined from three studies: (1) in-house RNA-seq data (*n* = 86) from the peripheral blood, nasal, and large airway epithelium of asthmatic and control individuals (S1); (2) multi-tissue RNA-seq data from Genotype-Tissue Expression (GTEx v6) from 53 human body sites [33] (*n* = 8555) (S2); and (3) randomly selected RNA-seq samples from the Sequence Read Archive (SRA) (*n* = 2000) (S3). The 2000 SRA RNA-seq samples are listed in Additional file 3: Table S3. Unless otherwise noted, we reported the percentage of reads averaged across three datasets.

RNA-seq data obtained from the three sources represent a large collection of tissue types and read diversity. We selected these three sources to most accurately model the precision and broad applicability of ROP. The in-house RNA-seq data were collected from 53 asthmatic individuals and 33 control individuals. RNA-seq libraries were prepared from total RNA with two types of RNA enrichment methods: (1) poly(A) enrichment libraries, applied to RNA from peripheral blood and nasal epithelium (*n* = 38); and (2) ribo-depletion libraries, applied to RNA from large airway epithelium (*n* = 49). The GTEx dataset was derived from 38 solid organ tissues, 11 brain subregions, whole blood, and three cell lines across 544 individuals. Randomly selected SRA RNA-seq samples included samples from whole blood, brain, various cell lines, muscle, and placenta. Length of reads from in-house data was 100 bp, read length in GTEx data was 76 bp, read length in SRA data was in the range of 36–100 bp. In total, 1 trillion reads (97 Tbp) derived from 10,641 samples were available for ROP (Additional file 1: Supplementary Methods and Additional file 2: Table S4). For counting purposes, the pairing information of the reads is disregarded and each read from a pair is counted separately.

**Fig. 2:**
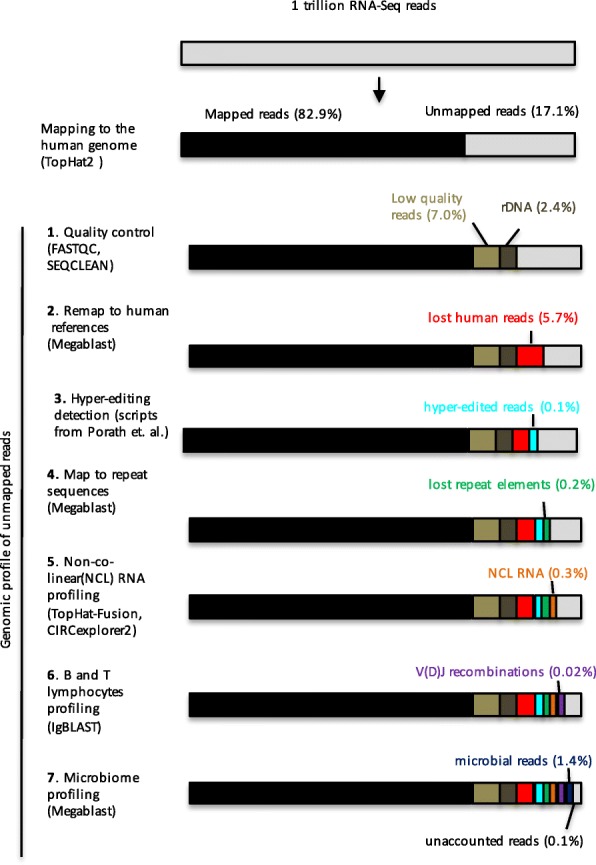
Genomic profile of unmapped reads across 10,641 samples and 54 tissues. Percentage of unmapped reads for each category is calculated as a fraction from the total number of reads. *Bars* of the plot are not scaled. Human reads (*black color*) are mapped to the reference genome and transcriptome via TopHat2. Unmapped reads are profiled using the seven steps of ROP protocol, described below. (1) Low quality/low-complexity (*light brown*) and reads matching rDNA repeating unit (*dark brown*) were excluded. (2) ROP identifies lost human reads (*red color*) from unmapped reads using a more sensitive alignment. (3) Hyper-edited reads are captured by hyper-editing the pipeline proposed in [17]. (4) ROP identifies lost repeat sequences (*green color*) by mapping unmapped reads onto the reference repeat sequences. (5) Reads arising from trans-spicing, gene fusion, and circRNA events (*orange color*) are captured by a TopHat-Fusion and CIRCexplorer2 tools. (6) IgBlast is used to identify reads spanning B and T cell receptor gene rearrangement in the variable domain (V(D)J recombinations) (*violet color*). (7) Microbial reads (*blue color*) are captured by mapping reads onto the microbial reference genomes

We used standard read mapping procedures to obtain mapped and unmapped reads from all three data sources. Read mapping for GTEx data was performed by the GTEx consortium using TopHat2 [25]. Following the GTEx consortium practice, we used TopHat2 to map reads from in-house and SRA studies. ROP protocol allows user to map the reads with their RNA-seq aligner of choice. High-throughput mapping using TopHat2 [25] recovered 83.1% of all reads from three studies (Fig. 2a), with the smallest fraction of reads mapped in the SRA study (79% mapped reads). From the unmapped reads, we first excluded low-quality/low-complexity reads and reads mapping to the rDNA repeating unit, which together accounted for 7.0% and 2.4% of all reads, respectively (Fig. 2b). We were then able to align unmapped reads to human reference sequences (5.7% of all reads, Fig. 2c) and identify “hyper-edited” reads (0.1% of all reads Fig. 2d). We then referenced repeat sequences (0.2% of all reads, Fig. 2d), reads identified as NCL RNAs (circRNAs, gene fusion, or trans-splicing) (0.3% of all reads, Fig. 2e), and reads mapped to recombined BCRs and TCRs (0.02% off all reads, Fig. 2f). The remaining reads were mapped to the microbial sequences (1.4% off all reads, Fig. 2g). Following the seven steps of ROP, the origins of 99.9% of reads were identified. The genomic profile of unmapped reads for each dataset is separately reported in Additional file 2: Table S5. Uncategorized reads from SRA samples are freely available at https://smangul1.github.io/recycle.RNA.seq/. This resource allows the bioinformatics community to further increase the number of reads with known origin.

### The ROP protocol identifies lost human reads

Some human reads may remain unmapped due to the heuristic nature of high-throughput aligners [12, 13]. As shown by Baruzzo et al., even the best performing RNA-seq aligners fail to map at least 10% of reads simulated from the human references. We used the slower and more sensitive Megablast aligner on this subset of unmapped reads. This method allows us to filter an additional 5.7% of human reads. One-fourth of the lost human reads are within the TopHat2 threshold (edit distance ≤ 2). Other reads missed by contained additional mismatches and/or short gaps (Additional file 4: Figure S1).

Using both mapped and unmapped reads across the studies, we classified on average 7.5% of the RNA-seq reads as repetitive sequences originated from various repeat classes and families (Additional file 4: Figure S2). We observe Alu elements to have 33% relative abundance, which was the highest among all the repeat classes. Among DNA repeats, hAT-Charlie was the most abundant element with 50% relative abundance (Additional file 4: Figure S3). Among SVA retrotransposons, SVA-D was the most abundant element with 50% relative abundance (Additional file 4: Figure S4). Consistent with repEnrich [34], when using in-house data, we observe the differences in proportions of L1 and Alu elements between poly(A) and ribo-depletion libraries. Among the repeat reads, poly(A) samples have the highest fraction of reads mapped to Alu elements and ribo-depleted samples have the highest fraction mapped to L1 elements (Additional file 4: Figure S5). Among the GTEx tissues, testis showed significantly higher expression of SVA F retrotransposons compared to other GTEx tissues (*p* = 2.46 × 10^− 33^) (Additional file 4: Figure S6). Furthermore, we observe high co-expression of Alu elements and L1 elements across GTEx tissues (R^2^ = 0.7615) (Additional file 4: Figure S7).

### ROP identifies hyper-edited reads

Using standard read mapping approaches, some human reads may remain unmapped due to “hyper editing.” An extremely common post-transcriptional modification of RNA transcripts in human is adenosine-to-inosine (A-to-I) RNA editing [35]. We define a read as hyper-edited if the number of A-to-G mismatches exceeds 5% of its length. Adenosine deaminases acting on RNA (ADARs) proteins can modify a genetically encoded A into an inosine I. I is read by the cellular machinery as a guanosine (G), and, in turn, sequencing of I results in G where the corresponding DNA sequencing reads A. Current methods to detect A-to-I editing sites identify such A-to-G mismatches using the alignment of RNA-seq reads to the genome. Reads with excessive (“hyper”) editing are usually rejected by standard alignment methods. In this case, many A-to-G mismatches obscure the genomic origin of these reads.

We have identified hyper-edited reads by using the pipeline proposed in Porath et al. [17]. This hyper-editing pipeline transforms all As into Gs, in both the unmapped reads and the reference genome. Next, the pipeline realigns the transformed RNA-seq reads and the transformed reference genome. The method then recovers original sequences and searches for dense clusters of A-to-G mismatches.

A total of 201,676,069 hyper-edited reads were identified across all samples from the three studies. As a control for the detection, we calculated the prevalence of all six possible nucleotide substitutions and found that 79.9% (201,676,069/252,376,867) of the detected reads were A-to-G mismatches (Additional file 4: Figure S8). The in-house RNA-seq samples have an even higher (96.1%) rate of A-to-G mismatches. This massive over-representation of mismatches strongly suggests that these reads resulted from ADAR mediated RNA editing. In addition, we found that the nucleotide sequence context of the detected editing sites complies with the typical sequence motif of ADAR targets (Additional file 4: Figure S9) supports the identification of the sites as true products of editing by ADAR.

### The ROP protocol complements transcriptome profiling by non-co-linear RNAs

The ROP protocol is able to detect NCL reads via TopHat-Fusion [36] and CIRCexplorer2 [37] tools from three classes of events: (1) reads spliced distantly on the same chromosome supporting trans-splicing events; (2) reads spliced across different chromosomes supporting gene fusion events; and (3) reads spliced in a head-to-tail configuration supporting circRNAs. On average, we observed 816 trans-splicing events, 7510 fusion events, and 930 circular events per individual sample supported by > 1 read. Over 90% of non-co-linear events were supported by fewer than ten samples (Additional file 4: Figure S10). We used a liberal threshold, based on number of reads and individuals, because our interest is the mapping of all reads. However, a more stringent cut off is recommended for confident identification of NCL events, especially in clinical settings.

Based on the in-house RNA-seq data, we observe that the library preparation technique strongly affects the capture rate of NCL transcripts. To compare the number of NCL events, we subsampled unmapped reads to 4,985,914 for each sample, which corresponded to the sample with the smallest number of unmapped reads among in-house RNA-seq samples. We observed an average increase of 92% of circRNAs in samples prepared by ribo-depletion compared to poly(A) protocol (*p* value = 3 × 10^− 12^) (Additional file 4: Figure S11). At the same time, we observed an average 43% decrease of trans-splicing and fusion events in samples prepared by ribo-depletion when compared to those prepared using a poly(A) protocol (*p* value < 8 × 10^− 4^) (Additional file 4: Figure S11). However, because the tissues differed between protocols (e.g. nasal vs large airway epithelium), this effect might be due in part to tissue differences in NCL events. We view the tissue differences effect to be unlikely. We previously showed that gene expression profiles of nasal airway tissue largely recapitulate expression profiles in the large airway epithelium tissue [38].

Furthermore, many NCL events will not be captured by poly-A selection. Therefore, we expect systematic differences in NCL abundance between capture methods. There were no statistically significant differences (*p* value > 5 × 10^− 3^) between NCL events in cases and in controls. We have compared number of NCL reads across GTEx tissue and we observe the highest fraction of NCL reads across pancreas samples with 0.75% of reads classified as NCL reads. While interesting, we caution that this observation could be driven by both biological and technical artifacts and requires further validation. In all other tissue types, ROP classified approximately 0.3% reads as NCL reads, which corresponds to 160,000 reads per sample (Additional file 4: Figure S12).

### ROP identifies microbial and immune reads and differentiates tissue types and disease status

Reads mapped to BCR and TCR loci and unmapped reads were used to survey the human adaptive immune repertoires in health and disease. We first used the mapped reads to extract reads entirely aligned to BCR and TCR genes. Using IgBlast [28], we identified unmapped reads with extensive somatic hyper mutations (SHM) and reads arising from V(D)J recombination. After we identified all the reads with human origin, we detected microbial reads by mapping the remaining reads onto microbial reference genomes and phylogenetic marker genes. Here, the total number of microbial reads obtained from the sample is used to estimate microbial load. We use MetaPhlAn2 [29] to assign reads on microbial marker genes and determine the taxonomic composition of the microbial communities.

Using in-house RNA-seq data, we compare immunological and microbial profiles across asthmatics and unaffected controls for the peripheral blood, nasal and large airway epithelium tissues. A total of 339 bacterial taxa were assigned with Metaphlan2 [29] across all studies and are freely available at https://smangul1.github.io/recycle.RNA.seq/.

Using Metaphlan2, we detected bacterial reads in all GTEx tissues except testis, adrenal gland, heart, brain, and nerve. We also observe no bacteria reads in the following cell lines: EBV-transformed lymphocytes(LCLs), Cells-Leukemia (CML), and Cells-Transformed fibroblasts cell lines. On average, we observe 1.43 ± 0.43 phyla assigned per sample. All samples were dominated by the phylum Proteobacteria (relative genomic abundance of 73% ± 28%). Other phyla detected included Acidobacteria, Actinobacteria, Bacteroidetes, Cyanobacteria, Fusobacteria, and Firmicutes. Consistent with previous studies, we observe the nasal epithelium is dominated by Actinobacteria phyla (particularly the *Propionibacterium* genus) [39] and the large airway epithelium is dominated by Proteobacteria phyla [40] (Additional file 2: Table S6). As a positive control for virus detection, we used GTEx samples from EBV-transformed lymphoblastoid cell lines (LCLs). ROP detected EBV virus across all LCL samples. An example of a coverage profile of EBV virus for one of the LCL samples is presented in Additional file 4: Figure S13. Additionally, we have investigated the amount of microbial reads that originate from the reagents used to construct the RNA-seq libraries [41]. We investigated number of reads mapped to Enterobacteria phage phiX174, which is routinely used as a part of the sequencing protocol. We observe no traces of Enterobacteria phage phiX174 in 95.6% of the samples. Other samples contain 1 in 1 million reads mapped to Enterobacteria phage.

We assess combinatorial diversity of the BCR and TCR repertoires by examining the recombination of the of V and J gene segments from the variable region of BCR and TCR loci. We used per sample alpha diversity (Shannon entropy) to incorporate the total number of VJ combinations and their relative proportions into a single diversity metric. We observed a mean alpha diversity of 0.7 among all the samples for immunoglobulin kappa chain (IGK). Spleen, minor salivary gland, and small intestine (terminal ileum) were the most immune diverse tissue, with corresponding IGK alpha diversity of 86.9, 52.05, and 43.96, respectively (Additional file 4: Figures S14 and S15). Across all the tissues and samples, we obtained a total of 312 VJ recombinations for IGK chains and 194 VJ recombinations for immunoglobulin lambda (IGL) chains. Inferred recombinations are freely available at https://smangul1.github.io/recycle.RNA.seq/.

Joint analysis of unmapped reads offered by the ROP protocol provides the opportunity to interrogate relationships between different features; for example, exploring the interactions between the immune system, microbiome, and gene expression [42]. To explore interactions between the immune system and microbiome, we compared immune diversity against microbial load across in-house samples. Microbes trigger immune responses, eliciting proliferation of antigen-specific lymphocytes. This dramatic expansion skews the antigen receptor repertoire in favor of a few dominant clonotypes and decreases immune diversity [43]. Therefore, we reasoned that antigen receptor diversity in the presence of microbial insults should shrink. In line with our expectation, we observed that the combinatorial immune diversity of the IGK locus was negatively correlated with viral load (Pearson coefficient r = − 0.55, *p* value = 2.4 × 10^− 6^), consistent also for bacteria and eukaryotic pathogens across BCR and TCR loci (Additional file 4: Figure S16).

**Fig. 3:**
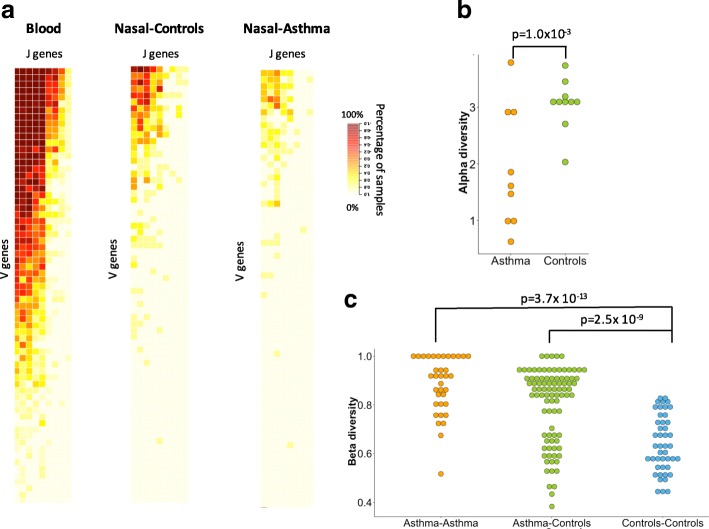
Combinatorial diversity of IGK locus differentiates disease status. **a** *Heatmap* depicting the percentage of RNA-seq samples supporting of particular VJ combination for whole blood (*n* = 19), nasal epithelium of healthy controls (*n* = 10), and asthmatic individuals (*n* = 9). Each *row* corresponds to a V gene and each *column* corresponds to a J gene. **b** Alpha diversity of nasal samples is measured using the Shannon entropy and incorporates total number of VJ combinations and their relative proportions. Nasal epithelium of asthmatic individuals exhibits decreased combinatorial diversity of IGK locus compared to healthy controls (*p* value = 1 × 10^− 3^). **c** Compositional similarities between the nasal samples in terms of gain or loss of VJ combinations of IGK locus are measured across paired samples from the same group (Asthma, Controls) and paired samples from different groups (Asthma vs Controls) using Sørensen–Dice index. Lower level of similarity is observed between nasal samples of asthmatic individuals compared to unaffected controls (*p* value < 7.3 × 10^− 13^). Nasal samples of unaffected controls are more similar to each other than to the asthmatic individuals (*p* value < 2.5 × 10^− 9^)

Using in-house data, we compared alpha diversity of asthmatic individuals (*n* = 9) and healthy controls (*n* = 10). The combinatorial profiles of BCRs and TCRs in blood and large airway tissue provide no differentiation between case control statuses. Compared to nasal and large airways, blood yields increased number of combinations of gene segments, with 191 combinations, on average, per sample for IGK locus (Fig. 3a). Among nasal samples, we observed decreased alpha diversity for asthmatic individuals relative to healthy controls (*p* value = 10^− 3^) (Fig. 3b). Additionally, we used beta diversity (Sørensen–Dice index) to measure compositional similarities between samples, including gain or loss of VJ combinations of IGK locus. We observed higher beta diversity corresponding to a lower level of similarity across the nasal samples of asthmatic individuals in comparison to samples from unaffected controls (Fig. 3c, *p* value < 3.7 × 10^− 13^). Moreover, nasal samples of unaffected controls are significantly more similar than samples from the asthmatic individuals (Fig. 3c, *p* value < 2.5 × 10^− 9^). Recombination profiles of IGL locus and T cell receptor beta and gamma (TCRB and TCRG) loci yielded a similar pattern of decreased beta diversity across nasal samples of asthmatic individuals (Additional file 4: Figures S17–S19). Together the results demonstrate the ability of ROP to interrogate additional features of the immune system without the expense of additional TCR/BCR sequencing.

#### The impact of RNA-seq aligners and mapping parameters on the number of accounted reads

Following Baruzzo et al. [12], we have selected five RNA-seq aligners that were reported to map a minimum of 75% of the reads simulated from human transcriptome using recommended parameters (GSNAP [version 2017–09-30], HISAT2 [2.1.0], Novoalign [v3.05.01], TopHat2 [v2.1.1], and STAR [v2.5.3a]). We use simulated data, described in the section “Validation of accuracy of ROP’s read assignments,” comprising transcriptomic, repeat, immune, and microbial reads, to investigate the effect of RNA-seq aligner on the number of mapped reads. On average, 90.9% of simulated transcriptomic reads are mapped by RNA-seq aligners with default and tuned parameters. STAR (default and tuned setting) and Novoalign (tuned settings) are able to map > 99% of transcriptomic reads. For other categories of simulated reads, RNA-seq aligners were able to map 47.6% of reads (Additional file 4: Figure S20a).

We have investigated the effects that choice of RNA-seq aligner can have on the fraction of reads accounted by ROP. Overall, ROP increases the number of categorized reads by an average of 39.5% across different RNA-seq aligners. The best results were achieved by ROP using STAR (default and tuned setting), Novoalign (default and tuned settings), and HISAT2 (tuned setting), which allows ROP to account for > 99.0% of all reads from RNA-seq mixture (Additional file 4: Figure S20b). Both STAR and Novoalign perform extensive soft clipping (partial mapping), resulting in alignment of recombined V(D)J sequences, which are not part of the human genome (Additional file 4: Figure S21). This can partially explain the increased number of mapped reads produced by ROP when compared to the results from other tools. Notably, Novoalign with default and tuned settings mapped 0.6% of microbial reads to the human genome, which corresponds to false-positive hits (Additional file 4: Figure S20b).

In addition to simulated data, we randomly selected ten SRA samples to investigate the effect of RNA-seq aligner on the number of mapped reads from the real datasets. On average we observe 91.8% of reads mapped; the best performance is achieved by HISAT2 and STAR, which allow mapping of 93.1% and 92.7% of the reads, respectively (Additional file 4: Figure S22). Based on the achieved results and reported results in Baruzzo et al. [12], we recommend using HISAT2 and STAR aligners to map the RNA-seq reads and prepare unmapped reads. Novoalign is not recommended for use with ROP protocol due to its substantially longer running time, which makes it computationally infeasible.

## Conclusion

Our study is the first that systematically accounts for almost all reads, totaling 1 trillion, which are available via three RNA-seq datasets. We demonstrate the value of analyzing unmapped reads present in RNA-seq data for the study of NCL RNA editing, immunological, and microbiome profiles of a tissue. We developed a new tool (ROP) that accounts for 99.9% of the reads, a substantial increase compared to the 82.2% of reads accounted for using conventional protocols. We found that the majority of unmapped reads are human in origin and from diverse sources, including repetitive elements, A-to-I RNA editing, circRNAs, gene fusions, trans-splicing, and recombined BCR and TCR sequences. In addition to those derived from human RNA, many reads were microbial in origin and often occurred in numbers sufficiently large to study the taxonomic composition of microbial communities in the tissue type represented by the sample.

We found that both unmapped human reads and reads with microbial origins are useful for differentiating between type of tissue and status of disease. For example, we found that the immune profiles of asthmatic individuals have decreased immune diversity when compared to those of controls. Further, we used our method to show that immune diversity is inversely correlated with microbial load. This case study highlights the potential for producing novel discoveries, when the information in RNA-seq data is fully leveraged by incorporating the analysis of unmapped reads, without need for additional TCR/BCR or microbiome sequencing. The ROP profile of unmapped reads output by our method is not limited to RNA-seq technology and may apply to whole-exome and whole-genome sequencing. We anticipate that ROP profiling will have broad future applications in studies involving different tissue and disease types.

We observed large effects when using different library preparation protocols on NCL, immunological, and microbial profiles. For example, the poly(A) protocol better captures antibody transcripts by enriching for polyadenylated transcripts, while ribo-depletion protocols capture more circRNAs. The results presented here suggest that selection of a protocol impacts quality of analysis results and our study may guide the choice of protocol depending on the features of interest.

The ROP protocol facilitates a simultaneous study of immune systems and microbial communities and this novel method advances our understanding of the functional, interrelated mechanisms driving the immune system, microbiome, human gene expression, and disease etiology. We hope that future efforts will provide a quantitative and qualitative assessment of the immune and microbial components of disease across various tissues. Recent increase in read length and sequencing efficiency provides opportunity for studying individual microbial species and full TCR/BCR sequencing.

## Methods

### In-house RNA-seq data

For poly(A) selected samples (*n* = 38), we used a subset of Puerto Rican Islanders recruited as part of the on-going Genes-environments & Admixture in Latino Americans study (GALA II) [44–47]. Nasal epithelial cells were collected from behind the inferior turbinate with a cytology brush using a nasal illuminator. Whole blood was collected using PAXgene RNA blood tubes. RNA was isolated using PAXgene RNA blood extraction kits. For ribo-depleted samples (*n* = 49), adults aged 18–70 years were recruited to undergo research bronchoscopy. During bronchoscopy airway epithelial brushings, samples were obtained from third- to fourth-generation bronchi. RNA was extracted from the epithelial brushing samples using the Qiagen RNeasy mini-kit.

Poly(A) selected RNA-seq libraries (*n* = 38) were constructed using 500 ng of blood and nasal airway epithelial total RNA from nine atopic asthmatics and ten non-atopic controls. Libraries were constructed and barcoded with the Illumina TruSeq RNA Sample Preparation v2 protocol. Barcoded nasal airway RNA-seq libraries from each of the 19 individuals were pooled and sequenced as 2 × 100-bp paired-end reads across two flow cells of an Illumina HiSeq 2000. Barcoded blood RNA-seq libraries from each of the 19 participants were pooled and sequenced as 2 × 100-bp paired-end reads across four lanes of an Illumina Hiseq 2000 flow cell. Ribo-depleted RNA-seq libraries (*n* = 38) were constructed using 100 ng of isolated RNA of large airway epithelium total RNA from 61 samples. Libraries were constructed and barcoded with the TruSeq Stranded Total RNA using a Ribo-Zero Human/Mouse/Rat library preparation kit, per manufacturer’s protocol. Barcoded bronchial epithelial RNA-seq libraries were multiplexed and sequenced as 2 × 100-bp paired-end reads on an Illumina HiSeq 2500. We excluded 12 samples from further analyses due to high ribosomal RNA read counts (library preparation failure), leaving a total of 49 samples suitable for further analyses.

### GTEx RNA-seq data

We used RNA-seq data from the Genotype-Tissue Expression study (GTEx Consortium v.6) that corresponds to 8555 samples collected from 544 individuals from 53 tissues obtained from the Genotype-Tissue Expression study (GTEx v6). RNA-seq data are from Illumina HiSeq sequencing of 75-bp paired-end reads. The data were derived from 38 solid organ tissues, 11 brain subregions, whole blood, and three cell lines of postmortem donors. The collected samples are from adults matched for age across males and females. We downloaded the mapped and unmapped reads in BAM format from dbGap (http://www.ncbi.nlm.nih.gov/gap).

### SRA RNA-seq data

Samples (*n* = 2000) were randomly selected using SQLite database from R/Bioconductor package SRAdb (https://bioconductor.org/packages/release/bioc/html/SRAdb.html). We used a script from https://github.com/nellore/runs/blob/master/sra/define_and_get_fields_SRA.R to select run_accessions from the sra table with platform = “ILLUMINA,” library_strategy = “RNA-Seq,” and taxon_id = 9606 (human).

### Workflow to categorize mapped reads

We mapped reads onto the human transcriptome (Ensembl GRCh37) and genome reference (Ensembl hg19) using TopHat2 (v 2.0.13) with the default parameters. TopHat2 was supplied with a set of known transcripts (as a GTF formatted file, Ensembl GRCh37) using –G option. The mapped reads of each sample are stored in a binary format (.bam). ROP (gprofile.py) categorizes the reads into genomic categories (junction read, CDS, intron, UTR3, UTR5, introns, inter-genic read, deep a deep inter-genic read, mitochondrial read, and multi-mapped read) based on their compatibility with the features defined by Ensembl (GRCh37) gene annotations. ROP (rprofile.py) categorizes reads into repeat elements (classes and families) based on their compatibility with repeat instances defined by RepeatMasker annotation (RepeatMasker v3.3, Repeat Library 20,120,124). We count the number of reads overlapping V, D, J, and constant (C) gene segments of BCR and TCR loci using htseq-count (HTSeq v0.6.1).

### Workflow to categorize unmapped reads

We first converted the unmapped reads saved by TopHat2 from a BAM file into a FASTQ file (using samtools with bam2fq option). The FASTQ file of unmapped reads contains full read pairs (both ends of a read pair were unmapped) and discordant read pairs (one read end was mapped while the other end was unmapped). We disregarded the pairing information of the unmapped reads and categorized unmapped reads using the protocol’s seven steps. Reads identified at each step are filtered out.

1. *Quality control.* Low-quality reads, defined as reads that have quality < 30 in at least 75% of their base pairs, were identified by FASTX (v 0.0.13). Low-complexity reads, defined as reads with sequences of consecutive repetitive nucleotides, were identified by SEQCLEAN. As a part of the quality control, we also excluded unmapped reads mapped onto the rDNA repeat sequence (HSU13369 Human ribosomal DNA complete repeating unit) (BLAST+ 2.2.30). Starting from new release (ROP v1.0.8), low-quality reads are not filtered out; instead, these reads were marked as low quality in the read name and passed to the downstream analysis. An example of ROP v1.0.8 output is presented for two SRA RNA-seq samples in Additional file 2: Table S7.
2. *Remap to human references.* We remapped the remaining unmapped reads to the human reference genome (hg19) and transcriptome (known transcripts, Ensembl GRCh37) using Megablast (BLAST+ 2.2.30).
3. *Hyper-editing detection.* We used a hyper-editing pipeline (HE-pipeline http://levanonlab.ls.biu.ac.il/resources/zip/hyper_editing_scripts.zip), which is capable of identifying hyper-edited reads. Before proceeding, users are advised to prepare the reference and provide the necessary third-party tools by following the instructions in the README of HE-pipeline that is included with the scripts. Ensure that the output directory is set correctly in config_file.sh (it is acceptable to use a single output directory) and check that the list of input files has been prepared correctly. Details on how to run HE-pipeline are available here: https://github.com/smangul1/rop/wiki/How-to-run-hyper-editing-pipeline.
4. *Map to repeat sequences.* The remaining unmapped reads were mapped to the reference repeat sequences using Megablast (BLAST+ 2.2.30). The reference repeat sequences were downloaded from Repbase v20.07 (http://www.girinst.org/repbase/). Human repeat elements (humrep.ref. and humsub.ref) were merged into a single reference.
5. *NCL RNA profiling.* NCL events include three classes of events: reads supporting trans-splicing events that are spliced distantly on the same chromosome; reads supporting gene fusion events that are spliced across different chromosomes; and reads supporting circRNAs that are spliced in a head-to-tail configuration. To distinguish between these three categories, we use circExplorer2 (v2.0.13). CircExplorer2 relies on TopHat-Fusion (v2.0.13, bowtie1 v0.12.) and allows simultaneous monitoring of NCL events in the same run. To extract trans-spicing and gene fusion events from the TopHat-Fusion output, we ran a ruby custom script that is part of the ROP pipeline (NCL.rb).
6. *B and T lymphocytes profiling.* We used IgBlast (v. 1.4.0) with a stringent e-value threshold (e-value < 10^− 20^) to map the remaining unmapped reads onto the V(D)J gene segments of the of the BCR and TCR loci. Gene segments of BCRs and TCRs were imported from IMGT version: 3.1.17 (International ImMunoGeneTics information system). The IMGT database contains: V gene segments; D gene segments; and J gene segments.
7. *Microbiome profiling.* We used Megablast (BLAST+ 2.2.30) to align remaining unmapped reads onto the collection of bacterial, viral, and eukaryotic reference genomes. Bacterial and viral genomes were downloaded from NCBI (ftp://ftp.ncbi.nih.gov/). Genomes of eukaryotic pathogens were downloaded from EuPathDB (http://eupathdb.org/eupathdb/). We used MetaPhlAn2 (Metagenomic Phylogenetic Analysis, v 2.0) to obtain the taxonomic profile of microbial communities present in the sample.

### Reference databases

A detailed description of reference databases used by ROP is provided in the Supplemental Material.

### Comparing diversity across groups

First, we subsampled unmapped reads by only including reads corresponding to a sample with the smallest number of unmapped reads. Diversity within a sample was assessed using the richness and alpha diversity indices. Richness was defined as a total number of distinct events in a sample. We used the Shannon Index (SI), incorporating richness and evenness components, to compute alpha diversity, which is calculated as follows:

$$ \mathrm{SI}=-\sum \left(p\times {\mathit{\log}}_2(p)\right) $$

We used beta diversity (Sørensen–Dice index) to measure compositional similarities between the samples in terms of gain or loss in events. We calculated the beta diversity for each combination of the samples and we produced a matrix of all pairwise sample dissimilarities. The Sørensen–Dice beta diversity index is measured as $1-\frac{2\mathrm{J}}{\mathrm{A}+\mathrm{B}}$, where J is the number of shared events, while A and B are the total number of events for each sample, respectively.

## Additional files

Additional file 1: Supplementary methods. (PDF 789 kb)

Additional file 2: **Table S1.** The effect of altering order of ROP step on the classification accuracy. **Table S2.** Concordance of targeted TCRB-Seq and ROP based on three TCGA samples from kidney renal clear cell carcinoma (KIRC). **Table S4.** RNA-seq datasets overview. **Table S5.** Genomic profile of unmapped reads reported for each dataset (S1, S2, S3). **Table S6.** Relative genomic abundance of microbial taxa at different levels of taxonomic classification after removal of reads with human origin (average over all samples of three tissues, performed for in-house RNA-Seqdata). **Table S7.** Genomic profile of unmapped reads across two SRA RNA-seq samples using ROP v1.0.8. Percentage for each category is calculated as a fraction from the total number of reads. (PDF 100 kb)

Additional file 3: **Table S3.** The list of the 2000 SRA RNA-seq samples used in the analysis. SRA samples were randomly selected from the Sequence Read Archive (SRA). (CSV 22 kb)

Additional file 4: **Figure S1.** Edit distance of lost human reads. **Figure S2.** Profile of repeat elements based on repeat sequences inferred from mapped and unmapped reads (lost repeat reads). **Figure S3.** Profile of DNA repeats based on repeat sequences inferred from mapped and unmapped reads (lost repeat reads). **Figure S4.** Profile of SVA retrotransposons based on repeat sequences inferred from mapped and unmapped reads (lost repeat reads). **Figure S5.** Profile of repeat elements across poly(A) enrichment and ribodepletion libraries. **Figure S6.** Average number of SVA-F reads across GTEx tissues. **Figure S7.** Co-expression of Alu and L1 elements across GTEx tissues. **Figure S8.** Distribution 77 of hyper-edited reads. **Figure S9.** The sequence context of Figure S8. **Figure S10.** Distribution of NCL events across 10,641 samples. **Figure S11.** Number of NCL events across in-house tissues and library preparation protocols. **Figure S12.** Percentage of NCL reads across GTEx tissues (*n* = 54). **Figure S13.** An example of coverage plot of EBV virus. **Figure S14.** Number of VJ recombinations across GTEx human tissues for IGK chain. **Figure S15.** Number of VJ recombinations across GTEx human tissues for IGL chain. **Figure S16.** Association between microbial load and immune diversity. **Figure S17.** Combinatorial diversity of IGL locus differentiates disease status. **Figure S18.** Combinatorial diversity of TCRB locus differentiates disease status. **Figure S19.** Combinatorial diversity of TCRG locus differentiates disease status. **Figure S20.** The effect of RNA-seq aligner on the fraction of reads accounted by ROP. **Figure S21.** Relationship between the number of soft clipped RNA-seq reads (partially mapped reads) and the total number of reads. **Figure S22.** Number of the RNA-seq reads mapped to the human reference genome across five state-of-the-art RNA-seq aligners. (PDF 2344 kb)

## References

[1] Sultan M, Schulz MH, Richard H, Magen A, Klingenhoff A, Scherf M (2008). A global view of gene activity and alternative splicing by deep sequencing of the human transcriptome. Science..

[2] Cloonan N, Forrest AR, Kolle G, Gardiner BB, Faulkner GJ, Brown MK (2008). Stem cell transcriptome profiling via massive-scale mRNA sequencing. Nat Methods..

[3] Tang F, Barbacioru C, Wang Y, Nordman E, Lee C, Xu N (2009). mRNA-Seq whole-transcriptome analysis of a single cell. Nat Methods..

[4] Wang Z, Gerstein M, Snyder M (2009). RNA-Seq: a revolutionary tool for transcriptomics. Nat Rev Genet..

[5] Trapnell C, Williams BA, Pertea G, Mortazavi A, Kwan G, van Baren MJ (2010). Transcript assembly and quantification by RNA-Seq reveals unannotated transcripts and isoform switching during cell differentiation. Nat Biotechnol..

[6] Nicolae M, Mangul S, Mandoiu II, Zelikovsky A (2011). Estimation of alternative splicing isoform frequencies from RNA-Seq data. Algorithms Mol Biol..

[7] Pertea M, Pertea GM, Antonescu CM, Chang TC, Mendell JT, Salzberg SL (2015). StringTie enables improved reconstruction of a transcriptome from RNA-seq reads. Nat Biotechnol..

[8] Ardlie KG, Deluca DS, Segre AV, Sullivan TJ, Young TR, Gelfand ET (2015). The Genotype-Tissue Expression (GTEx) pilot analysis: Multitissue gene regulation in humans. Science..

[9] Li S, Tighe SW, Nicolet CM, Grove D, Levy S, Farmerie W (2014). Multi-platform assessment of transcriptome profiling using RNA-seq in the ABRF next-generation sequencing study. Nat Biotechnol..

[10] Seqc/Maqc-Iii Consortium (2014). A comprehensive assessment of RNA-seq accuracy, reproducibility and information content by the Sequencing Quality Control Consortium. Nat Biotechnol..

[11] Ozsolak F, Milos PM (2011). RNA sequencing: advances, challenges and opportunities. Nat Rev Genet..

[12] Baruzzo G, Hayer KE, Kim EJ, Di Camillo B, FitzGerald GA, Grant GR (2017). Simulation-based comprehensive benchmarking of RNA-seq aligners. Nat Methods.

[13] Siragusa E, Weese D, Reinert K (2013). Fast and accurate read mapping with approximate seeds and multiple backtracking. Nucleic Acids Res..

[14] Grabherr MG, Haas BJ, Yassour M, Levin JZ, Thompson DA, Amit I (2011). Full-length transcriptome assembly from RNA-Seq data without a reference genome. Nat Biotechnol..

[15] Blachly JS, Ruppert AS, Zhao W, Long S, Flynn J, Flinn I (2015). Immunoglobulin transcript sequence and somatic hypermutation computation from unselected RNA-seq reads in chronic lymphocytic leukemia. Proc Natl Acad Sci..

[16] Strauli NB, Hernandez RD (2016). Statistical inference of a convergent antibody repertoire response to influenza vaccine. Genome Med..

[17] Porath HT, Carmi S, Levanon EY (2014). A genome-wide map of hyper-edited RNA reveals numerous new sites. Nat Commun..

[18] Wu C-S, Yu CY, Chuang CY, Hsiao M, Kao CF, Kuo HC (2014). Integrative transcriptome sequencing identifies trans-splicing events with important roles in human embryonic stem cell pluripotency. Genome Res..

[19] Wang X-S, Prensner JR, Chen G, Cao Q, Han B, Dhanasekaran SM (2009). An integrative approach to reveal driver gene fusions from paired-end sequencing data in cancer. Nat Biotechnol..

[20] Jeck WR, Sharpless NE (2014). Detecting and characterizing circular RNAs. Nat Biotechnol..

[21] Kostic AD, Ojesina AI, Pedamallu CS, Jung J, Verhaak RG, Getz G (2011). PathSeq: software to identify or discover microbes by deep sequencing of human tissue. Nat Biotechnol..

[22] Chuang T-J, Wu CS, Chen Y, Hung LY, Chiang TW, Yang MY (2016). NCLscan: accurate identification of non-co-linear transcripts (fusion, trans-splicing and circular RNA) with a good balance between sensitivity and precision. Nucleic Acids Res..

[23] Brown SD, Raeburn LA, Holt RA (2015). Profiling tissue-resident T cell repertoires by RNA sequencing. Genome Med..

[24] Strauli N, Hernandez R (2016). Statistical inference of a convergent antibody repertoire response to influenza vaccine. Genome Med..

[25] Kim D, Pertea G, Trapnell C, Pimentel H, Kelley R, Salzberg SL (2013). TopHat2: accurate alignment of transcriptomes in the presence of insertions, deletions and gene fusions. Genome Biol..

[26] Andrews S. FastQC: a quality control tool for high throughput sequence data. 2010. Available online at: http://www.bioinformatics.babraham.ac.uk/projects/fastqc.

[27] Camacho C, Coulouris G, Avagyan V, Ma N, Papadopoulos J, Bealer K (2009). BLAST+: architecture and applications. BMC Bioinformatics..

[28] Ye J, Ma N, Madden TL, Ostell JM (2013). IgBLAST: an immunoglobulin variable domain sequence analysis tool. Nucleic Acids Res..

[29] Truong DT, Franzosa EA, Tickle TL, Scholz M, Weingart G, Pasolli E (2015). MetaPhlAn2 for enhanced metagenomic taxonomic profiling. Nat Methods..

[30] Salter SJ, Cox MJ, Turek EM, Calus ST, Cookson WO, Moffatt MF (2014). Reagent and laboratory contamination can critically impact sequence-based microbiome analyses. BMC Biol..

[31] Tausch SH, Renard BY, Nitsche A, Dabrowski PW (2015). RAMBO-K: rapid and sensitive removal of background sequences from next generation sequencing data. PLoS One..

[32] Li B, Li T, Pignon JC, Wang B, Wang J, Shukla SA (2016). Landscape of tumor-infiltrating T cell repertoire of human cancers. Nat Genet..

[33] GTEx Consortium (2015). The Genotype-Tissue Expression (GTEx) pilot analysis: Multitissue gene regulation in humans. Science..

[34] Criscione SW, Zhang Y, Thompson W, Sedivy JM, Neretti N (2014). Transcriptional landscape of repetitive elements in normal and cancer human cells. BMC Genomics..

[35] Bazak L, Haviv A, Barak M, Jacob-Hirsch J, Deng P, Zhang R (2014). A-to-I RNA editing occurs at over a hundred million genomic sites, located in a majority of human genes. Genome Res..

[36] Kim D, Salzberg SL (2011). TopHat-Fusion: an algorithm for discovery of novel fusion transcripts. Genome Biol..

[37] Zhang XO, Dong R, Zhang Y, Zhang JL, Luo Z, Zhang J (2016). Diverse alternative back-splicing and alternative splicing landscape of circular RNAs. Genome Res..

[38] Poole A, Urbanek C, Eng C, Schageman J, Jacobson S, O’Connor BP (2014). Dissecting childhood asthma with nasal transcriptomics distinguishes subphenotypes of disease. J Allergy Clin Immunol..

[39] Yan M, Pamp SJ, Fukuyama J, Hwang PH, Cho DY, Holmes S (2013). Nasal microenvironments and interspecific interactions influence nasal microbiota complexity and S. aureus carriage. Cell Host Microbe..

[40] Beck JM, Young VB, Huffnagle GB (2012). The microbiome of the lung. Transl Res..

[41] Strong MJ, Xu G, Morici L, Splinter Bon-Durant S, Baddoo M, Lin Z (2014). Microbial contamination in next generation sequencing: implications for sequence-based analysis of clinical samples. PLoS Pathog..

[42] Westermann AJ, Gorski SA, Vogel J (2012). Dual RNA-seq of pathogen and host. Nat Rev Microbiol..

[43] Spreafico R, Rossetti M, van Loosdregt J, Wallace CA, Massa M, Magni-Manzoni S (2016). A circulating reservoir of pathogenic-like CD4+ T cells shares a genetic and phenotypic signature with the inflamed synovial micro-environment. Ann Rheum Dis..

[44] Jin Y, Tam OH, Paniagua E, Hammell M (2015). TEtranscripts: a package for including transposable elements in differential expression analysis of RNA-seq datasets. Bioinformatics..

[45] Melé M, Ferreira PG, Reverter F, DeLuca DS, Monlong J, Sammeth M (2015). The human transcriptome across tissues and individuals. Science..

[46] Anders S, Pyl PT, Huber W (2014). HTSeq--A Python framework to work with high-throughput sequencing data. Bioinformatics..

[47] Tarailo-Graovac M, Chen N (2009). Using RepeatMasker to identify repetitive elements in genomic sequences. Curr Protoc Bioinformatics.

[48] Mangul S, Yang HT, Strauli N, Gruhl F, Porath HT, Hsieh K, et al. ROP: Dumpster Diving in RNA-sequencing to find the source of 1 trillion reads across diverse adult human tissues. Gene Expression Omnibus. 2018; https://www.ncbi.nlm.nih.gov/geo/query/acc.cgi?acc=GSE109313. 10.1186/s13059-018-1403-7 PMC5857127 29548336

[49] Mangul S, Yang HT, Strauli N, Gruhl F, Porath HT, Hsieh K, et al. ROP: Dumpster Diving in RNA-sequencing to find the source of 1 trillion reads across diverse adult human tissues. Gene Expression Omnibus. 2018; https://www.ncbi.nlm.nih.gov/geo/query/acc.cgi?acc=GSE109484. 10.1186/s13059-018-1403-7 PMC5857127 29548336

