# Supplementary material for: ROP: dumpster diving in RNA-sequencing to find the source of 1 trillion reads across diverse adult human tissues
Source: Genome Biol. 2018 Feb 15;19:36. doi: 10.1186/s13059-018-1403-7 (PMC5857127; doi:10.1186/s13059-018-1403-7)
Supplement: Supplementary file 1 — Supplementary methods. (PDF 789 kb) [file 13059_2018_1403_MOESM1_ESM.pdf]

Comprehensive analysis of RNA-sequencing to find the source of 1 trillion reads across diverse adult human tissues

Supplementary Methods

In-house RNA-Seq data

Subject Recruitment

Poly(A) selected RNA-Seq samples (n=38). In this analysis, we used a subset of Puerto Rican Islanders recruited as part of the on-going Genes-environments & Admixture in Latino Americans study (GALA II) [3–6]. We classified asthma by physician diagnosis and the presence of at least two symptoms (wheezing, coughing, or shortness of breath) during 2 years prior to the enrollment. All study subjects had no history of smoking or recent (within 4 weeks of recruitment) nasal steroid use. The study was approved by local institutional review boards, and written assent/consent was received from all subjects and, if applicable, parents of subjects under the age of legal consent.

Ribo-Zero RNA-Seq samples (n=49). Via community-based advertising, we recruited adults aged 18-70 years to participate in a study, in which they underwent research bronchoscopy. The study was approved by the University of California at San Francisco Committee on Human Research. Written informed consent was obtained from all subjects, and all studies were performed in accordance with the principles expressed in the Declaration of Helsinki.

Sample Collection

Poly(A) selected RNA-Seq samples (n=38). Methods for nasal epithelial cell collection and processing are described in Poole et al. [6]. Briefly, nasal epithelial cells were collected from behind the inferior turbinate with a cytology brush using a nasal illuminator. The collected brush was submerged in a mixture of RLT Plus lysis buffer and beta-mercaptoethanol, and frozen at -80 C until extraction was performed with a Qiagen Allprep RNA/DNA extraction kit (Qiagen, Valencia, CA). We collected 10ml of whole blood using PAXgene RNA blood tubes (PreAnalytiX, Valencia, CA) and isolated RNA using PAXgene RNA blood extraction kits, according to the manufacturers' protocol. Portions of the nasal airway epithelial whole transcriptome data were published in a previous manuscript [6].

Ribo-Zero RNA-Seq samples (n=49). During bronchoscopy airway epithelial brushings, samples were obtained from 3rd-4th generation bronchi. RNA was extracted from the epithelial brushing samples using the Qiagen RNeasy mini-kit (Qiagen, Valencia, CA), according to manufacturer's protocol.

Whole Transcriptome Sequencing

Poly(A) selected RNA-Seq samples (n=38). We constructed Poly-A RNA-seq libraries using 500 ng of blood and nasal airway epithelial total RNA from 9 atopic asthmatics and 10 non-atopic controls. Libraries were constructed and barcoded with the Illumina TruSeq

RNA Sample Preparation v2 protocol. Barcoded nasal airway RNA-seq libraries from each of the 19 subjects were pooled and sequenced as 2 x 100bp paired-end reads across two flow cells of an Illumina HiSeq 2000. Barcoded blood RNA-seq libraries from each of the 19 subjects were pooled and sequenced as 2 x 100bp paired end reads across 4 lanes of an Illumina HiSeq 2000 flow cell.

Ribo-Zero RNA-Seq samples (n=49). We used 100ng of isolated RNA from a total of 61 samples to construct ribo-depleted RNA-seq libraries using the TruSeq Stranded Total RNA with Ribo-Zero Human/Mouse/Rat library preparation kit, per manufacturer's protocol. Barcoded bronchial epithelial RNA-seq libraries were multiplexed and sequenced as 2 x 100bp paired end reads on an Illumina HiSeq 2500. On average, 37 million reads were generated per sample. We excluded 12 samples from further analyses due to high ribosomal RNA read counts (library preparation failure), leaving a total of 49 samples suitable for further analyses.

<u>**GTEx RNA-Seq data**</u>

We used RNA-Sequencing data from Genotype-Tissue Expression study (GTEx Consortium v.6) corresponding to 8,555 samples collected from 544 individuals from 53 tissues obtained from Genotype-Tissue Expression study (GTEx v6). RNA-Seq data is from Illumina HiSeq sequencing of 75 bp paired-end reads. The data was derived from 38 solid organ tissues, 11 brain subregions, whole blood, and three cell lines of postmortem donors. The collected samples are from adults matched for age across males and females. We

downloaded the mapped and unmapped reads in BAM format from dbGap (http://www.ncbi.nlm.nih.gov/gap).

SRA RNA-Seq data

Samples (n=2000) were randomly selected using SQLite database from R/Bioconductor package SRAdb (https://bioconductor.org/packages/release/bioc/html/SRAdb.html). We have used a script from https://github.com/nellore/runs/blob/master/sra/define_and_get_fields_SRA.R to select run_accessions from the sra table with platform = ’ILLUMINA’, library_strategy = ’RNA-Seq’, and taxon_id = 9606 (human).

Workflow to categorize the mapped reads

Data preparation

ROP assumes that Illumina adapter sequences were deleted from the input sequencing data.

Map reads onto human genome and transcriptome

We used standard read mapping procedures to obtain mapped and unmapped reads from all three data sources. Read mapping for GTEx data was performed by the GTEx consortium using TopHat2 [7]. Following the GTEx consortium practice, we used TopHat v. 2.0.12 with ENSEMBL GRCh37 transcriptome and hg19 build to map reads from in-house

and SRA studies. High-throughput mapping using TopHat2 [7] recovered 83.1% of all reads from three studies (Fig. 2.a), with the smallest fraction of reads mapped in the SRA study (79% mapped reads). We have investigated the effect of RNA-Seq aligner choice on the number of mapped reads and performance of ROP (See main text).

.

We mapped reads onto the human transcriptome (Ensembl GRCh37) and genome reference (Ensembl hg19) using tophat2 (v 2.0.13) with the default parameters. Tophat2 was supplied with a set of known transcripts (as a GTF formatted file, Ensembl GRCh37) using –G option. The mapped reads of each sample are stored in a binary format (.bam).

Categorize mapped reads into genomic categories

ROP categorizes the reads into genomic categories based on the compatibility of each read from the pair with the features defined by Ensembl (GRCh37) gene annotations. First, we determined CDS, UTR3, UTR5 coordinates. We downloaded annotations for CDS, UTR3, UTR5 from UCSC Genome Browser (http://genome.ucsc.edu/cgi-bin/hgTables) in BED (browser extensible data) format. Next, we used gene annotations (a GTF formatted file, Ensembl GRCh37) to determine intron coordinates and inter-genic regions. We defined two types of inter-genic regions: '(proximate) inter-genic' region (1Kb from the gene boundaries) and 'deep inter-genic' (beyond a proximity of 1Kb from the gene boundaries).

Next, we checked the compatibility of the mapped reads with the defined genomic features, as follows:

a. Read mapped to multiple locations on the reference genome is categorized as a multi-mapped read.
b. Read fully contained within the CDS, intron, UTR3, or UTR5 boundaries of a least one transcript is classified as a CDS, intronic, UTR3, or UTR5, respectively.
c. Read simultaneously overlapping UTR3 and UTR5 regions is classified as a UTR read.
d. Read spanning exon-exon boundary is defined as a junction read.
e. Read mapped outside of gene boundaries and within a proximity of 1Kb is defined as a (proximal) inter-genic read.
f. Read mapped outside of gene boundaries and beyond the proximity of 1Kb is defined as a deep inter-genic read.
g. Read mapped to mitochondrial DNA (MT tag in hg19) is classified as a mitochondrial read.
h. Reads from a pair mapped to different chromosomes are classified as a fusion read.

Scripts to categorize mapped reads into genomic categories are distributed with ROP protocol.

Categorize mapped reads overlapping repeat instances

Mapped reads were categorized based on the overlap with the repeat instances defined by RepeatMasker annotation (RepeatMasker v3.3, Repeat Library 20120124). RepeatMasker masks the repeats using the RepBase library: (http://www.girinst.org/repbase/update/index.html), which contains prototypic sequences representing repetitive DNA from different eukaryotic species. We use GTF files generated from the RepeatMasker annotations by Jin, Ying, et al. [3] and downloaded from: http://labshare.cshl.edu/shares/mhammelllab/www-data/TEToolkit/TE_GTF/hg19_rmsk_TE.gtf.gz

Following Melé, Marta, et al. [4], repeat elements overlapping CDS regions are excluded from the analysis. We filtered out 6,873 repeat elements overlapping CDS regions. Prepared repeat annotations (bed formatted file) are available at https://drive.google.com/file/d/0Bx1fyWeQo3cORi1UNWhxOW9kYUk/view?pref=2&pli=1

The prepared repeat annotations contain 8 Classes and 43 Families. Number of elements per family and class represented below (Supplemental Methods Table SM1):

**classID**	**N**

DNA	458223
LINE	1478382
LTR	707384
RC	2226
SVA	3582
RNA	717
Satellite	8950
SINE	1765403

Supplemental Methods Table SM1. Number of repeat elements per class. Repeat instances are defined by RepeatMasker (RepeatMasker v3.3, Repeat Library 20120124) based on RepBase library. RepBase library contains prototypic sequences representing repetitive DNA from different eukaryotic species.

familyID	n
acro	44
Alu	1173282
centr	2272
CR1	60577
Deu	1262
DNA	4609

**Dong-R4**	554
**ERV**	579
**ERV1**	172612
**ERVK**	10446
**ERVL**	159606
**ERVL-MaLR**	343266
**Gypsy**	18553
**hAT**	15418
**hAT-Blackjack**	19578
**hAT-Charlie**	251618
**hAT-Tip100**	30204
**Helitron**	2226
**L1**	937636
**L2**	461296
**LTR**	2322
**Merlin**	55
**MIR**	589496
**MuDR**	1978
**Penelope**	51
**PiggyBac**	2352
**RNA**	717
**RTE**	17617

**RTE-BovB**	651
**Satellite**	6247
**SINE**	1363
**SVA_A**	257
**SVA_B**	465
**SVA_C**	279
**SVA_D**	1358
**SVA_E**	232
**SVA_F**	991
**TcMar**	5354
**TcMar-Mariner**	16253
**TcMar-Tc2**	8098
**TcMar-Tigger**	102706
**telo**	387

Supplemental Methods Table SM2. Number of repeat elements per family. Repeat instances are defined by RepeatMasker (RepeatMasker v3.3, Repeat Library 20120124) based on RepBase library.

We determined the coordinates of repeat elements (*class_id* and *family_id attributes from the GTF file*) from the repeat annotations. Next, we checked the compatibility of the mapped reads with the repeat instances. We disregarded the pairing information for the

unmapped reads and count each end as a separate read. Reads entirely mapped to the corresponding repeat instance are counted. Scripts to categorize mapped reads based on the overlap with the repeat instances are distributed with ROP protocol.

Categorize mapped reads overlapping B cell receptor (BCR) and T cell receptor (TCR) loci

We used the gene annotations (Ensembl GRCh37) to extract BCR and TCR genes. We extracted gene annotations of the 'constant' (labeled as IG_C_gene, Ensembl GRCh37), 'variable' (labeled as IG_V_gene, Ensembl GRCh37), 'diversity' (labeled as IG_D_gene, Ensembl GRCh37), and 'joining' genes (labeled as IG_J_gene, Ensembl GRCh37) of BCR and TCR loci. We excluded the BCR and TCR pseudogenes (labeled as IG_C_pseudogene, IG_V_pseudogene, IG_D_pseudogene, IG_J_pseudogene, TR_C_pseudogene, TR_V_pseudogene, TR_D_pseudogene, and TR_J_pseudogene). In addition, we excluded the patch contigs *HG1592_PATCH and HG7_PATCH,* as they are not part of the Ensembl hg19 reference, and reads are not mapped on the patch contigs by high throughput aligners. After following the filtering steps described above, we extracted a total of 386 immune genes: 207 BCR genes and 179 TCR genes. The gene annotations for antibody genes (GTF formatted file) are available at https://drive.google.com/file/d/0Bx1fyWeQo3cObFZNT3kyQlZUS1E/view?pref=2&pli=1

The number of VDJ genes per locus is reported in the Table 3.

	C domain	V domain	D domain	J domain

IGH locus	8	55	38	6
IGK locus	1	46	-	5
IGL locus	4	37	-	7
TCRA locus	1	46	-	57
TCRB locus	1	39	0	8
TRG locus	2	9	-	5
TRD locus	1	3	11	4

Supplemental Methods Table SM3. The number of VDJ genes for each antibody chains. Antibody genes were extracted from the gene annotations (Ensembl GRCh37).

The list of the genes encoding the C region of the BCR and TCR chains is presented in Supplemental Methods Table SM4.

Name of the chain	Genes encoding for the C region of the chain
IG@ locus	
α heavy IG chain	IGHA1, IGHA2
δ heavy IG chain	IGHD
γ heavy IG chain	IGHG1, IGHG2, IGHG3, IGHG4
ε heavy IG chain	IGHE
µ heavy IG chain	IGHM

**κ light IG chain**	IGKC
**λ light IG chain**	IGLC1, IGLC2, IGLC3, IGLC7
**TCR@ locus**	
**α TCR chain**	TRAC
**Β TCR chain**	TRBC2
**γ TCR chain**	TRGC1, TRGC2
**δ TCR chain**	TRDC

Supplemental Methods Table SM4. List of the genes encoding the C region of the BCR and TCR chains. Genes were extracted from the gene annotations (Ensembl GRCh37).

The number of reads mapping to each C-V-D-J genes was *obtained by counting the number of* sequencing reads that align, with high confidence, to each of the genes (HTSeq v0.6.1) [5]. Script "htseq-count" is supplied with the gene annotations for BCR and TCR genes (genes_Ensembl_GRCh37_BCR_TCR.gtf) and a bam file. The bam file contains reads mapped to the human genome and transcriptome using tophat2 (See Section ***"Map reads onto human genome and transcriptome"*** for details). The script generates individual gene counts by examining the read compatibility with BCR and TCR genes. We chose a conservative setting (--mode=intersection-strict) to handle reads overlapping more than one feature. Thus, a read overlapping several genes simultaneously is marked as a read with no feature and is excluded from the consideration.

<u>Workflow for categorizing the unmapped reads</u>

We first converted the unmapped reads saved by tophat2 from a BAM file into a FASTQ file (using bamtools). The FASTQ file of unmapped contain full read pairs (both ends of a read pair were unmapped) and discordant read pairs (one read end was mapped while the other end was unmapped). We disregarded the pairing information of the unmapped reads and categorize unmapped reads using the following steps:

A. Quality Control

Low quality reads, defined as reads that have quality lower than 30 in at least 75% of their base pairs, were identified by in house script . Low complexity reads, defined as reads with sequences of consecutive repetitive nucleotides, are identified by SEQCLEAN. As a part of the quality control, we also excluded unmapped reads mapped onto the rRNA repeat sequence (HSU13369 Human ribosomal DNA complete repeating unit) (BLAST+ 2.2.30). We have masked the HSU13369 rRNA sequence using Repeat Masker via online interface at http://www.repeatmasker.org/cgi-bin/WEBRepeatMasker.

The report from Repeat Masker is provided below:

```
==================================================
file name: RM2_rRNA.fa_1508888790
sequences:      1
total length:   42999 bp (42999 bp excl N/X-runs)
GC level:    58.38 %
```

```
bases masked:   23004 bp ( 53.50 %)
==================================================
      number of   length percentage
      elements*  occupied of sequence
--------------------------------------------------
SINEs:       25    5881 bp 13.68 %
   ALUs     25    5881 bp 13.68 %
   MIRs      0      0 bp  0.00 %

LINEs:        2     541 bp  1.26 %
   LINE1     2     541 bp  1.26 %
   LINE2     0      0 bp  0.00 %
   L3/CR1     0      0 bp  0.00 %

LTR elements:    2     250 bp  0.58 %
   ERVL      0      0 bp  0.00 %
   ERVL-MaLRs   0      0 bp  0.00 %
   ERV_classI   2     250 bp  0.58 %
   ERV_classII  0      0 bp  0.00 %

DNA elements:    1     388 bp  0.90 %
  hAT-Charlie   0      0 bp  0.00 %
```

TcMar-Tigger 0 0 bp 0.00 %

Unclassified: 0 0 bp 0.00 %

Total interspersed repeats: 7060 bp 16.42 %

Small RNA: 2 6862 bp 15.96 %

Satellites: 0 0 bp 0.00 %

Simple repeats: 66 8941 bp 20.79 %

Low complexity: 3 141 bp 0.33 %

==

We prepared the index from masked rRNA repeat sequence using makeblastdb and makembindex from BLAST+. We used the following command for makeblastdb:

- makeblastdb -parse_seqids -dbtype nucl -in <fasta file>.

We used the following command for makembindex:

- makembindex -input <fasta file> -output <index> -iformat blastdb

B. Mapping unmapped reads onto the human references.

We remapped the unmapped reads to the human reference sequences using Megablast (BLAST+ 2.2.30). We mapped reads onto the following references:

- Reference transcriptome (known transcripts), Ensembl GRCh37
- Reference genome, hg19 Ensembl

We prepared the index from each reference sequence using makeblastdb and makembindex. We mapped the reads separately onto each reference in the order listed above. Reads mapped to the reference genome and transcriptome were merged into a 'lost human reads' category. The following options were used to map the reads using Megablast: for each reference: task = megablast, use_index = true, perc_identity = 90, outfmt = 6, max_target_seqs =1, e-value = $1e^{-05}$.

C. Identification of hyper-edited reads

We have used hyper-editing pipeline (HE-pipeline http://levanonlab.ls.biu.ac.il/resources/zip), which is capable of identifying hyper-edited reads. When running HE-pipeline, additional changes can be made to parallelize the scripts for use with UCLA's Hoffman2 cluster. Before proceeding, follow the instructions in the README that is included with the scripts to prepare the reference and provide the necessary third-party tools. Ensure that the output directory is set correctly in config_file.sh (it is acceptable to use a single output directory), and check that the list of input files has been prepared correctly.

Details on how to run HE-pipeline are available here: https://github.com/smangul1/rop/wiki/How-to-run-hyper-editing-pipeline

D. Mapping unmapped reads onto the repeat sequences

We filtered out the reads that failed QC and lost human reads. The remaining reads were mapped to the reference repeat sequences. The reference repeat sequences were downloaded from Repbase v20.07 (http://www.girinst.org/repbase/). Human repeat elements (humrep.ref and humsub.ref) were merged into a single reference. We prepared the index from the merged repeat reference using makeblastdb and makembindex from BLAST+. In total, we obtained sequences for 1,117 repeat elements. The following options were used to map the reads using the Megablast: task = megablast, use_index = true, perc_identity = 90, outfmt = 6, max_target_seqs = 1, e-value = $1e^{-05}$. Blast hits with alignment length shorter than 80% of the read length were discarded (corresponding to 80bp of the 100bp read).

The repeat elements from humrep.ref and humsub.ref were classified into families and classes using RepeatMasker annotations (hg19_rmsk_TE_prepared_noCDS.bed). Repetitive reads identified from the unmapped reads were confirmed by directly applying RepeatMasker [6].

***E. Workflow to* detect 'non-co-linear' reads (trans-splicing, gene fusions, and circRNAs)**

We divide non-co-linear reads into three categories:

1) gene fusion characterized by reads that map on different chromosomes
2) trans-splicing events characterized by reads that map on the same chromosome, but are at least 1 Mb apart from each other
3) circRNAs characterized by reads that map in a head-to-tail configuration on the same chromosome

To distinguish between these three categories, we make use of circExplorer2 (Zhang et al., 2016), which was recently identified as one of the best tools to detect circRNAs (Hansen et al., 2015). CircExplorer2 relies on TopHat-Fusion and thus allows also the monitoring NCL events in the same run. TopHat-Fusion (v2.0.13, bowtie1 v0.12.9) and circExplorer2 (v2.2.4) were invoked with the following commands:

```
$ tophat2 -o tophat-output-directory -p 4 --fusion-search --keep-fasta-order --bowtie1 --no-coverage-search bowtie1-index fastq-file
```

```
$ python CIRCexplorer2 parse -t TopHat-Fusion -o circrna-output-folder tophat-output-directory/accepted_hits.bam
```

```
$ python CIRCexplorer2 annotate -r ensemble-reference.txt -g genome.fa circrna-output-folder
```

To separate potential gene and trans-fusions from the TopHat-Fusion output, we ran a ruby custom script, which is part of the ROP pipeline.

F. Mapping unmapped reads onto the V(D)J recombinations of B and T cell receptors

Gene segments of B cell receptors (BCR) and T cell receptors (TCR) were imported from IMGT (International ImMunoGeneTics information system): (http://www.imgt.org/vquest/refseqh.html#V-D-J-C-sets).

IMGT database contains:

- Variable (V) gene segments
- Diversity (D) gene segments
- Joining (J) gene segments

Unmapped reads categorized by step (A)-(D) were filtered out. We used IgBLAST (v. 1.4.0) with stringent e-value threshold (e-value < 10^{-20}) to map the remaining high-quality unmapped reads onto the V(D)J regions of the of the BCR and TCR loci. Reference files with BCR and TCR VDJ gene segments are distributed with ROP protocol and available at https://drive.google.com/folderview?id=0Bx1fyWeQo3cOTkhKdHFDb3c5MjA&usp=sharing

The complete list of the references is presented in Supplemental Methods Table SM5.

Name of the reference file	Description of the gene

**BCR heavy chain**	
**IGHV.fa**	V genes of BCR heavy chain
**IGHD.fa**	D genes of BCR heavy chain
**IGHJ.fa**	J genes of BCR heavy chain
**BCR light chains**	
**IGLV.fa**	V genes of BCR lambda chain
**IGLJ.fa**	J genes of BCR lambda chain
**IGKV.fa**	V genes of BCR kappa chain
**IGKJ.fa**	J genes of BCR kappa chain
**TCR chains**	
**TCRAV.fa**	V genes of TCR alpha chain
**TCRAJ.fa**	J genes of TCR alpha chain
**TCRBV.fa**	V genes of TCR beta chain
**TCRBD.fa**	D genes of TCR beta chain
**TCRBJ.fa**	J genes of TCR beta chain
**TCRGV.fa**	V genes of TCR gamma chain
**TCRGJ.fa**	J genes of TCR gamma chain
**TCRDV.fa**	V genes of TCR delta chain
**TCRDD.fa**	D genes of TCR delta chain
**TCRDJ.fa**	J genes of TCR delta chain

Supplemental Methods Table SM5. List of the references files prepare for V-D-J from BCR and TCR loci.

We prepared the index from each reference sequence using makeblastdb and makembindex from BLAST+. The following options were used to map the reads using IgBLAST: -germline_db_V; germline_db_D; -germline_db_J; -organism=human; -outfmt = 7; –evalue = 1e-20.

The number of genes and gene alleles per antibody locus is presented in Supplemental Methods Table SM6.

	V domain	D domain	J domain
IGH locus	**136**(370)	**27**(34)	**9**(16)
IGK locus	**100**(124)	-	**5**(9)
IGL locus	**70**(111)	-	**7**(10)
TCRA locus	**54**(112)	-	**61**(68)
TCRB locus	**77**(160)	**2**(3)	**14**(16)
TRG locus	**14**(26)	-	**5**(6)
TRD locus	**8**(22)	**0**(0)	**1**(4)

Supplemental Methods Table SM6. The number of V-D-J genes and gene alleles per antibody locus. Number of genes is presented in bold and number of gene alleles is

presented in parenthesis. Gene and gene alleles of B cell receptors (BCR/IG) and T cell receptors (TCR) were imported from IMGT.

We assessed combinatorial diversity of the antibody repertoire by looking at the recombinations of the VJ gene segments of BCR and TCR loci. We extracted the reads spanning the V-J gene boundaries.

G. Identification of microbial reads

Unmapped reads mapping in step (A -E) were filtered out. The remaining reads were high-quality non-human reads used to profile the taxonomic composition of the microbial communities. We used MetaPhlAn2 (Metagenomic Phylogenetic Analysis, v 2.0) to assign reads on microbial genes and to obtain a taxonomic profile. The database of the microbial marker genes is provided by MetaPhlAn. We run MetaPhlAn in two stages as follow: the first stage identifies the candidate microbial reads (i.e., reads hitting a marker), while the second stage profiles metagenomes in terms of relative abundances – the commands used are as follow:

- metaphlan.py <fastq> <map> --input_type multifastq --bowtie2db bowtie2db/mpa -t reads_map --nproc 8 --bowtie2out
- metaphlan.py --input_type blastout <bowtie2out.txt> -t rel_ab <tsv>

The output of the first stage is a file containing a list of candidate microbial reads with the microbial taxa assigned (.map file). The second stage outputs the taxonomic profile (taxa

detected and its relative abundance, in tab separated format (.tsv file). We used taxa detected from stage 2 to extract the reads associated with it in stage 1.

In addition to MetaPhlAn2 we used to create the curated database of taxa-specific genes, we mapped the reads onto the entire reference genomes of microbial organisms. We used Megablast (BLAST+ 2.2.30) to align reads onto the collection of bacterial, viral, and eukaryotic pathogens reference genomes. Bacterial and viral genomes were downloaded from NCBI ftp://ftp.ncbi.nih.gov/ on February 1, 2015. Genomes of eukaryotic pathogens were downloaded from EuPathDB database, which is available at: http://eupathdb.org/eupathdb/.

The following parameters were used for the megablast alignment: e-value = 10^{-5}, perc_identity = 90. The Megablast hits shorter than 80% of the input read sequence were removed (corresponding to 80bp of the 100bp read).

<u>Comparing diversity across groups</u>

First, we sub-sampled unmapped reads to the number of reads corresponding to a sample with the smallest number of unmapped reads. Diversity within a sample was assessed using the richness and alpha diversity indices. Richness was defined as a total number of distinct ***events*** in a sample. We used Shannon Index (SI), incorporating richness and evenness components, to compute alpha diversity, which is calculated as follows:

$$\mathrm{SI} = -\sum (p \times \log_2(p))$$

We used beta diversity (Sørensen–Dice index) to measure compositional similarities between the samples in terms of gain or loss in the events. We calculated the beta diversity for each combination of the samples, and we produced a matrix of all pairwise sample dissimilarities. The Sørensen–Dice beta diversity index is measured as $1-\frac{2J}{A+B}$, where J is the number of shared events, while A and B are the total number of events for each sample, respectively.

Percentage of unmapped reads calculation

We calculated the percentage of unmapped reads using the following formula:

$$P_{\text{unmapped}} = \frac{(N_{\text{ud}} + (N_{\text{uc}} \times 2))}{(N_{\text{total}} \times 2\ )}$$

where,

N_{ud} – number of discordant unmapped reads (one end is mapped, while the other end is unmapped);

N_{uc} – number of unmapped read pairs (both ends are unmapped);

N_{total} – total number of read pairs (fragments).

Identification of reads originated during the library construction

We have investigated the number of reads that could have originated during library construction. We have used the database of primer and adapter sequences prepared by

FASTQC, a quality control tool for high throughput sequence data. The database was downloaded from: http://www.bioinformatics.babraham.ac.uk/projects/fastqc/

BWA index was prepared with the following command:

- bwa index contaminant_list.custom.fa

Reads containing the adapter or primer sequencing with the read are identified using the following command:

- bwa mem /rop/data/contaminant_list.custom.fa <unmapped.fasta> | samtools view -F 4 | grep "NM:i:0"

We have investigated the number of the reads containing adapter or primer sequence across 2000 SRA samples. On average we observe 0.01% of the reads containing adapter or primer sequence. Above 90% of the samples have less than 0.01% of adapter or primer sequences.

Prepared database of of primer and adapter sequences and corresponding bowtie2 index are distributed with ROP software package.

Simulated RNA-Seq data as a mixture of transcriptomic, repeat, immune, and microbial reads

We simulated RNA-Seq data as a mixture of transcriptomic, repeat, immune, and microbial reads using wgsim read simulator (https://github.com/lh3/wgsim). We use referenced human transcript sequences (Homo_sapiens.GRCh38.79.gtf) to simulate transcriptomics reads. We used referenced repeat sequences to simulate repeat reads. Immune transcripts were simulated using ImRep-simulation tool[8]. We have use microbiome sequences downloaded from NCBI to simulate the microbial reads.

To simulate human transcriptomics reads we first obtain the sequences of the transcripts using the following command:

```
$gffread genes.gtf -g genome.fa -w isoforms_GRCh37_Ensembl.fasta
```

We simulate 618 human transcriptomics reads from known isoforms using the following command:

```
$wgsim -r 0.01 -e 0.01 -1 100 -2 100 -A 0 -N 618 isoforms_GRCh37_Ensembl.fasta
reads_TR_1.fastq reads_TR_2.fastq >log 2>log2
```

To simulate repeat reads we used the repbase database of repeat elements (distribuuted with ROP)

```
$ wgsim -r 0.01 -e 0.01 -1 100 -2 100 -A 0 -N 250 repbase.fa repeats_1.fastq
repeats_2.fastq
```

We simulated 250 immune reads from recombined B and T cell receptor transcripts, as described in Mangul et al. (2017).

We simulated microbial reads from viral and bacterial reference genomes.

```
wgsim -r 0.01 -e 0.01 -1 100 -2 100 -A 0 -N 250 ~/project/Viruses/viruses.fa virus_1.fastq
virus_2.fastq >log
```

```
wgsim -r 0.01 -e 0.01 -1 100 -2 100 -A 0 -N 250 ~/project/Bacteria/bacteria.fa
bacteria_1.fastq bacteria_2.fastq >log_bacteria.txt
```

TCRB-Seq

We have downloaded TCRB-Seq data from https://clients.adaptivebiotech.com/pub/Liu-2016-NatGenetics. Data was prepared by Li, Bo, et al. (2017). It contains 3 TCRB-Seq

samples from 3 individuals from TCGA study. From TCRB-Seq data we have extracted VJ recombinations using the following script:

https://github.com/smangul1/rop-project/blob/master/validation/experimental_data/TCRB-SEQ/extract_VJ.sh

For sample TCGA-CZ-4862 we have extracted 54 recombinations of V and J gene segments . For sample TCGA-CZ-5463 we have extracted 53 recombinations of V and J gene segments. For sample TCGA-CZ-5985 we have extracted 53 recombinations of V and J gene segments

ROP was able to identify between 1 and 4 VJ recombinations. All recombinations inferred by ROP were confirmed by TCRB-Seq. Per sample recombinations are available here https://github.com/smangul1/rop-project/tree/master/validation/experimental_data

The robustness of the ROP results against changing the thresholds for each of the ROP steps

We have performed the robustness analysis to investigate the impact of the thresholds used in each step of the ROP approach. For each ROP step, we have reported number of reads identified under different thresholds. The results are presented as cumulative frequency plots.

a

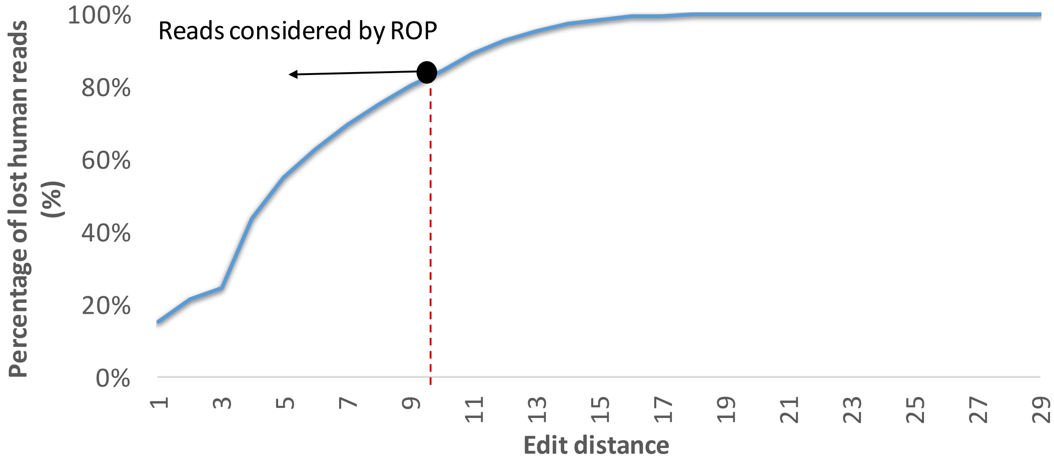
Reads considered by ROP
Percentage of lost human reads (%)
100%
80%
60%
40%
20%
0%
1 3 5 7 9 11 13 15 17 19 21 23 25 27 29
Edit distance

b

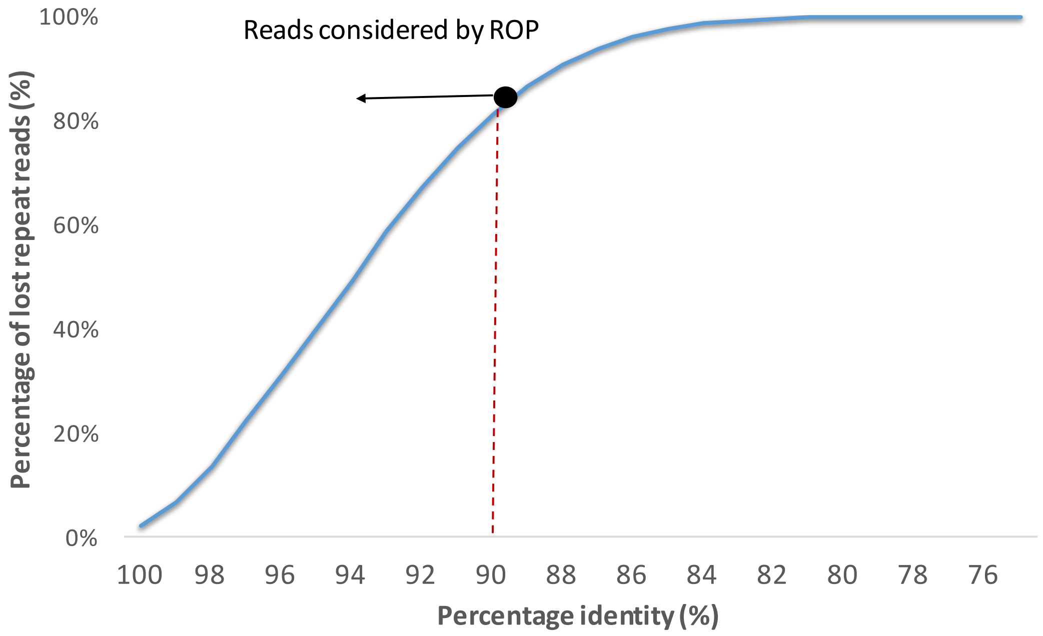
Reads considered by ROP
Percentage of lost repeat reads (%)
100%
80%
60%
40%
20%
0%
100 98 96 94 92 90 88 86 84 82 80 78 76
Percentage identity (%)

c

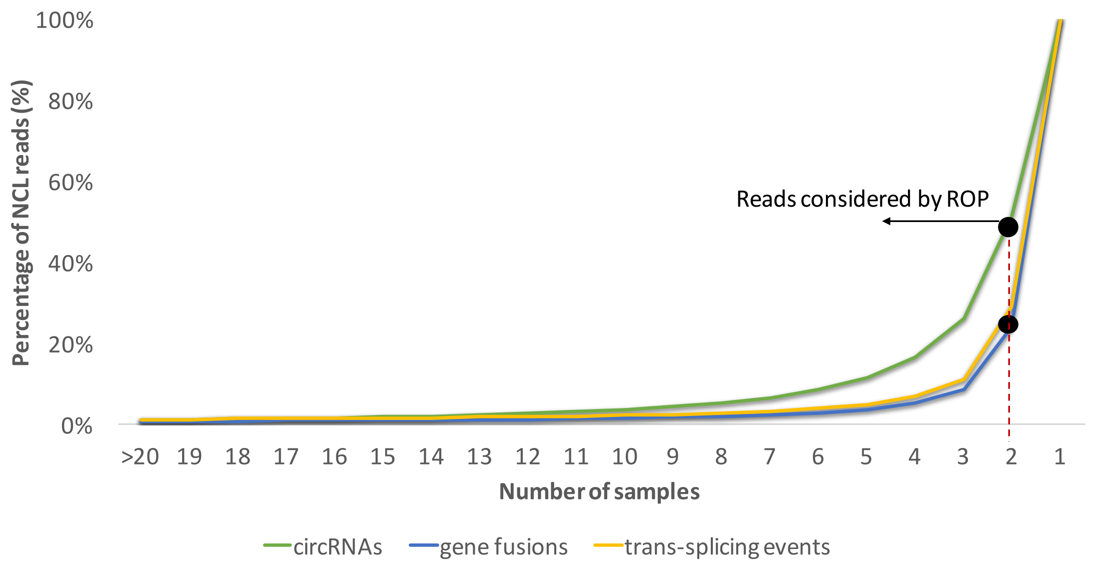
100%
80%
60%
40%
20%
0%
Percentage of NCL reads (%)
Reads considered by ROP
>20 19 18 17 16 15 14 13 12 11 10 9 8 7 6 5 4 3 2 1
Number of samples
circRNAs
gene fusions
trans-splicing events

d

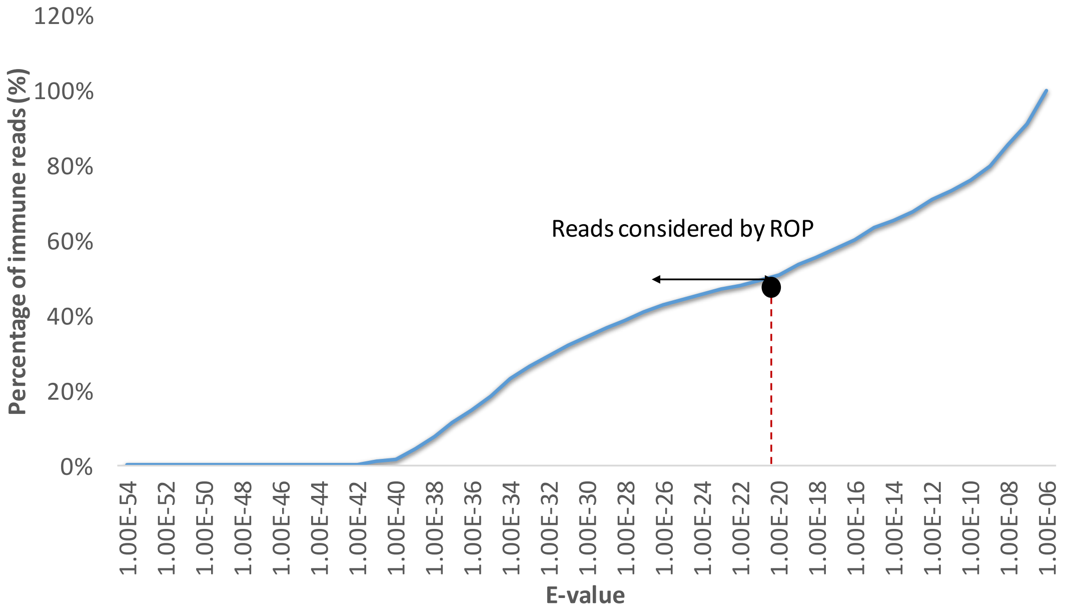
120%
100%
80%
60%
40%
20%
0%
Percentage of immune reads (%)
Reads considered by ROP
1.00E-54 1.00E-52 1.00E-50 1.00E-48 1.00E-46 1.00E-44 1.00E-42 1.00E-40 1.00E-38 1.00E-36 1.00E-34 1.00E-32 1.00E-30 1.00E-28 1.00E-26 1.00E-24 1.00E-22 1.00E-20 1.00E-18 1.00E-16 1.00E-14 1.00E-12 1.00E-10 1.00E-08 1.00E-06
E-value

e

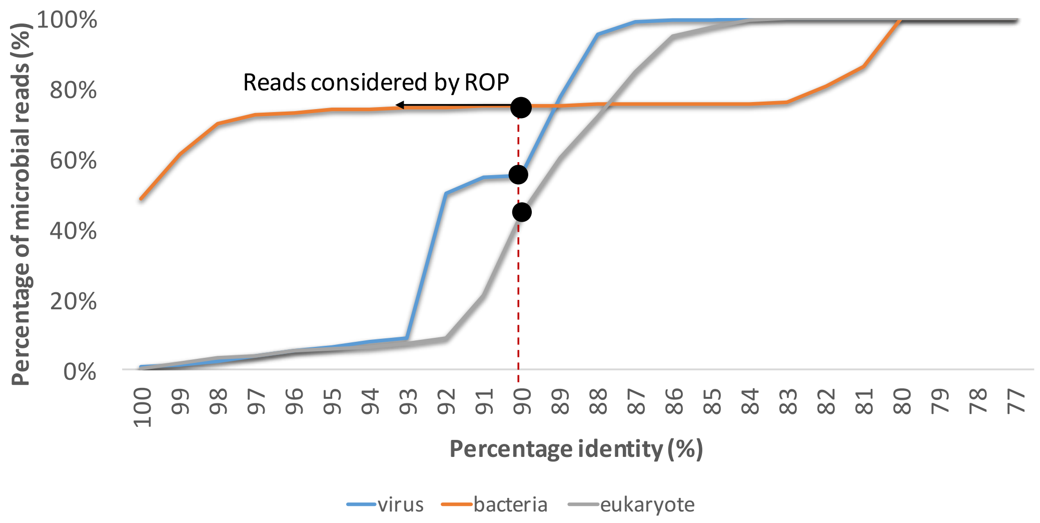

Supplemental Methods Figure SM1. Percentage of reads identified under different threshold values. Results are presented as cumulative frequency plots for each step of ROP. ROP threshold is highlighted with red line.

The percentages are the averages across 87 samples. (a) Step 2 (Remap to human references). Cumulative frequency plot reporting the percentage of lost human reads averaged across all samples (y-axis) identified under different threshold (edit distance) (x-axis). Edit distance was calculated as the minimum number of operations required to transform a read sequence into the corresponding reference subsequence. Reads are grouped by edit distance with the transcriptome or the genome reference. (b) Step 3 (Map to repeat sequences). Cumulative frequency plot reporting the percentage of lost repeat reads (y-axis) identified under different threshold averaged across (percentage identity) (x-axis). (c) Step 4 (NCl RNA profiling). Cumulative frequency plot of the percentage of NCL reads averaged across all samples (y-axis) identified under different

thresholds (number of reads supporting NCL event) (x-axis). Results are reported separately for circRNAs, gene fusions and trans-splicing events. (d) Step 5 (B and T cell receptors profiling). Cumulative frequency plot reporting the percentage of immune reads averaged across all samples (y-axis) identified under different threshold (e-value) (x-axis). (e) Step 6 (Microbiome profiling). Cumulative frequency plot reporting the percentage of microbial reads averaged across all samples (y-axis) identified under different threshold (percentage identity) (x-axis). Results are reported separately for viral, bacterial and eukaryotic reads.

The impact of ROP step ordering on the read classification

We have investigated the effect of the ordering on read classification. Ordering of ROP steps will have an effect only when references of each step share homologous sequences. For each ROP step, we have swapped its order with another ROP step. For example, we considered swapping 'Remapping to human references' reads and 'QC' steps. Before swapping, 'Remapping to human references' was number 2 in the queue. After swapping, it became number 1.

We observed a major effect of swapping 'Remapping to human references' with all other steps. For example, swapping 'Remapping to human references' and 'QC' steps results in classifying 79.6% of rRNA reads as lost human reads. Similarly, swapping 'Remapping to human references' and 'Microbiome profiling' steps results in classifying 0.2% of the lost

human reads as microbiome reads. In other words, this swap produces a 27.8% increase of microbiome reads. Similarly, considering ‘B and T lymphocytes profiling’ prior to ‘Remapping to human references’ produces a 50.8% increase of identified immune reads. Considering partial mapping of BCR and TCR reads prior to the ‘Remapping to human references’ step may produce many false positives. Swapping other steps of ROP resulted in minor effects (i.e., <1% of reads from each category were effected).

<u>The effect of different library preparation techniques over the ability to detect B and T cell receptor transcripts.</u>

Using in-house data, we investigated the effect of different library preparation techniques over the ability to detect B and T cell receptor transcripts. We compared the alpha diversity in large airway samples to nasal samples (Supplemental Fig. S16). Decreased alpha diversity in large airway samples compared to nasal (2.5 for nasal versus 1.0 for large airway) could correspond to an overall decrease in percentage of immune reads. This effect can be attributed to the ribo-depletion protocol not enriching for polyadenylated antibody transcripts. Alternatively, it may result from clonal expansion of certain clonotypes responding to the cognate antigen.

<u>Distribution of low quality reads across categories of ROP</u>

We investigated the distribution of low quality reads across categories of ROP. On average, 40.3% of low quality reads were assigned to ROP categories. Majority of low quality reads are classified as microbial and lost human reads, corresponding to 21.4% and 18.7%, respectively, of all low quality reads (**Supplemental Methods Table SM7**). The fraction of low quality reads among all the reads across ROP categories was 21.6% on average. The largest contribution of low quality reads was detected in lost human reads and microbial reads, which represented 44.0% and 43.6%, of all low quality reads respectively (**Supplemental Methods Table SM8**).

sample	rRNA repeat	lost human reads	lost repeat elements	NCL RNAs	recombined BCR/TCRs	microbial reads
SRR3703207	0.00%	20.15%	0.03%	0.05%	0.00%	8.30%
SRR5831944	0.00%	17.21%	0.08%	0.12%	0.02%	34.59%

Supplemental Methods Table SM7. Distribution of low quality reads across categories of ROP. Low quality reads are defined as reads that have quality lower than 30 in at least 75% of their base pairs. The percentage for each category is calculated as a fraction

from the total number of low quality reads. Results are presented for SRR3703207 and SRR5831944 SRA RNA-Seq samples.

sample	rRNA repeat	lost human reads	lost repeat elements	NCL RNAs	recombined BCR/TCRs	microbial reads
SRR3703207	0.0%	63.7%	39.1%	4.5%	0.0%	45.1%
SRR5831944	0.0%	24.3%	8.3%	1.2%	30.8%	42.0%

Supplemental Methods Table SM8. Contribution of low quality reads across categories of ROP. Low quality reads are defined as reads that have quality lower than 30 in at least 75% of their base pairs. The percentage for each category is calculated as a fraction from the total number of reads in each ROP category. Results are presented for SRR3703207 and SRR5831944 SRA RNA-Seq samples.

<u>Analysis of read pairs discordant across ROP classes</u>

Using both simulated and real data we have investigated the number of read pairs discordant across ROP classes, where the reads from the same pair are classified into different classes. In the simulated data, no discordant read pairs across classes were detected, except 0.18% of discordant reads pairs across transcriptomic and repeat

categories. We detected an average of 0.47% discordant read pairs across transcriptomic and repeat categories across SRA RNA-seq samples. The number of discordant read pairs across microbiome and human sequences was 0.001% in SRA RNA-Seq samples. Discordant read pairs across microbiome and human sequences can be due to spurious mapping or due to viral integration sites[9].

Complexity analysis using Capture Recapture Model

Given a sequencing experiment, the Read Origin Protocol (ROP) attempts to classify every sequenced read in the experiment to an "origin" class. These origins can be considered to be features of interest (e.g., exons, retroviral, immune, or bacterial). Since every read is assigned to only one class, we can consider the reads assigned to a specific class to be a random sample from the population of possibilities within that class. This leads us to consider statistical models for population sampling, which are known as "capture-recapture" models [10].

Using capture-recapture models allows us to make statistical inferences on several quantities of interest. Of primary interest is the total number of possibilities in the feature. We shall refer to this as the feature size but is commonly known in the statistics literature as species richness [10,11]. We also consider the number of identified possibilities within a feature as a function of the number of reads. We call this the complexity of the feature, in line with the notation of Daley and Smith [12]. The rate of change in the complexity curve is proportional to the probability the next read in a previously unobserved class [13]. This quantity is commonly known in statistics literature as the

mathematical coverage [14], but to avoid confusion with sequencing coverage, we call this the discovery probability [15]. One minus the discovery probability will be called the saturation of the feature.

Statistical Model

Suppose we sequence N reads from an experiment. There are C feature classes, represented in the sequencing library with proportions $\pi_1, \dots, \pi_C$. Features may overlap, so it is not necessary that the proportions sum to one. The features are all known and defined beforehand. This trait is in contrast to the number of classes within each feature. Within each feature c, there are a fixed but unknown number of classes; Sc represented in the experiment. Within the feature, these are represented with relative proportions

$$p_1, \dots, p_{S_C}, \sum_{i=1}^{S_C} p_i = 1$$

If we are interested in the relative proportions within the experiment, we multiply the relative proportion within the feature by the relative abundance of the feature within the experiment.

The problem is that we only have information on the classes that were sequenced in the experiment. We observed $D_C \leq S_C$ classes with observed frequencies x_i = # reads from class i with $\sum_{i=1}^{S_C} x_i = N_C$ and $\sum_{c=1}^{C} N_c = N$.

The problem of estimating the complexity is to estimate the number of expected distinct classes observed as a function of reads sequenced. We use the non-parametric empirical Bayesian? approach of Daley and Smith [12] to estimate the feature complexity curve. The limit of the feature complexity curve can be regarded as an estimate of the feature size

[16].

The discovery probability of the observed experiment is the sum of the relative proportions of the unobserved classes,

$$\sum_{\square=1}^{s_c} \boldsymbol{p_i 1(x_i = 0)}.$$

The non-parametric empirical Bayes estimator for this quantity is given by the Good Turing formula, $(\sum_{i=1}^{S_c} \frac{1(x_i=1)}{N_C})$.

Read Complexity Analysis

We first examine the read complexity as determined by the mapped start position of the first end in the read pair. We observe little difference between the two libraries for the single end complexity (Supplemental Methods Figure SM3). We observe only an average of 20% and 29% of the reads that can be mapped at the sequenced read depth. We estimate that all libraries are an average of 58% saturated; that is, we observed 58% of the abundance. This is natural since one would naturally sequence the most abundant reads first.

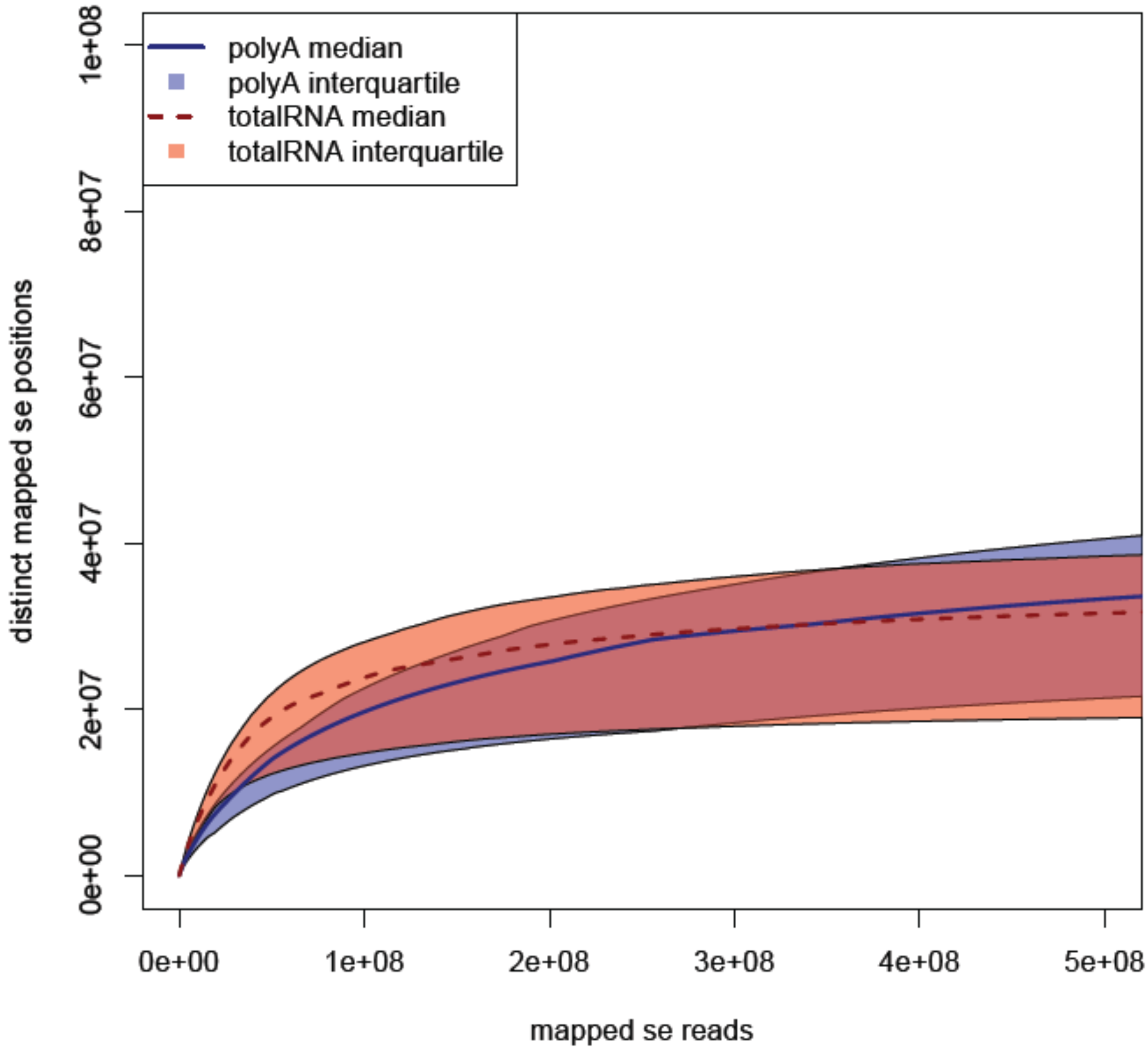

Supplemental Methods Figure SM3. Single end read complexity medians and interquartile ranges across the two library preparations.

Annotated Feature Complexity Analysis

The mapped reads can be assigned to features within the genome. These include exons, introns, coding sequences (CDS), and untranslated regions (UTR). In this section we shall investigate the complexity of these features, which can be interpreted as estimating the

transcriptional diversity within these libraries.

As expected, more exons, CDSs, and UTRs were observed per sequenced fragment for the polyA libraries than for the totalRNA libraries. Yet all libraries are very saturated. Most of the abundant classes within these features have already been observed, and the unobserved features are extremely rare. This is in line with the common practice of sequencing a few tens of millions of reads for inferring differential expression.

To compare the saturation across libraries, we extrapolated the saturation to a common value. The saturation is asymptotically normal [17], and the sequencing depth is sufficiently high that we can use a standard t-test to investigate differences. The polyA libraries are more saturated when all the features for all libraries are extrapolated out to 100 million observations (exons: p = 3.764E-16; CDS: p = 1.036E-14; UTR: p = 5.183E-14; more significant differences were observed at lower depths, indicating that the differences are not artifacts of the sampling depth).

Despite the large saturation for all features across libraries, a multitude of unobserved classes remain (Supplemental Methods Table SM7). This means that most of the unobserved classes are exceedingly rare. For example, we estimate that there are an average of 41,990 unobserved exons in the polyA libraries. There is an average remaining abundance of $1 - 0.9988 = 0.0012$, implying that the average abundance of the unobserved exons is $\frac{0.0012}{41990} = 2.86\ E - 8$. Since, on average, a read has $2 \cdot 0.176 = 0.352$ probability of overlapping an exon, the average abundance of the unobserved exons is

1E-8 and the total abundance, 0.00042, gives the marginal probability that the next sequenced read is a new exon. For the totalRNA libraries, the average abundance of the unobserved exons is 3.2E-8. Similarly, we calculated the average abundance of the unobserved CDS for polyA and totalRNA libraries as 1.84E-8 and 7.78E-8, respectively, and for UTRs it was 1.1E-8 and 6.48E-8.

Feature	Mean hits		Mean observed		Mean saturation		Mean estimated total	
	polyA	totalRNA	polyA	totalRNA	polyA	totalRNA	polyA	totalRNA
Exons	10310521		110553		0.9969		145950	
	17713362	5745436	115507	107498	0.9988	0.9956	157497	138829
CDS	4791394		105820		0.984		131521	
	8804113	2316884	116068	99500	0.9977	0.9756	144062	123788
UTR	4359596		33165		0.9948		43136	
	8035082	2093047	37448	30524	0.99913	0.99209	49849	38997

Supplemental Methods Table SM7. Mean number of observations, distinct observed classes, observed saturation, and estimated total number of classes for exons, CDS, and

UTR Features.

Finally, we examined differences of diversity between case and controls for a fixed tissue type and library type. The results are quite anticlimactic, as we found little differences between cases and controls for extrapolated saturation and feature diversity. This indicates that there are little differences in transcriptome diversity between the two groups of case and controls. Alternatively, it may indicate that the differences between the two groups are so small that a much larger cohort is required to accurately infer the disparity.

Genomic profiles across library preparation protocols

Similar to Li, S. et al. we observed that library preparation has a strong effect on the fraction of both mapped and lost human reads mapping to CDS and intronic regions. Genomic profile of mapped and unmapped reads across library preparation protocols is presented in **Supplemental Methods Figure SM4.**

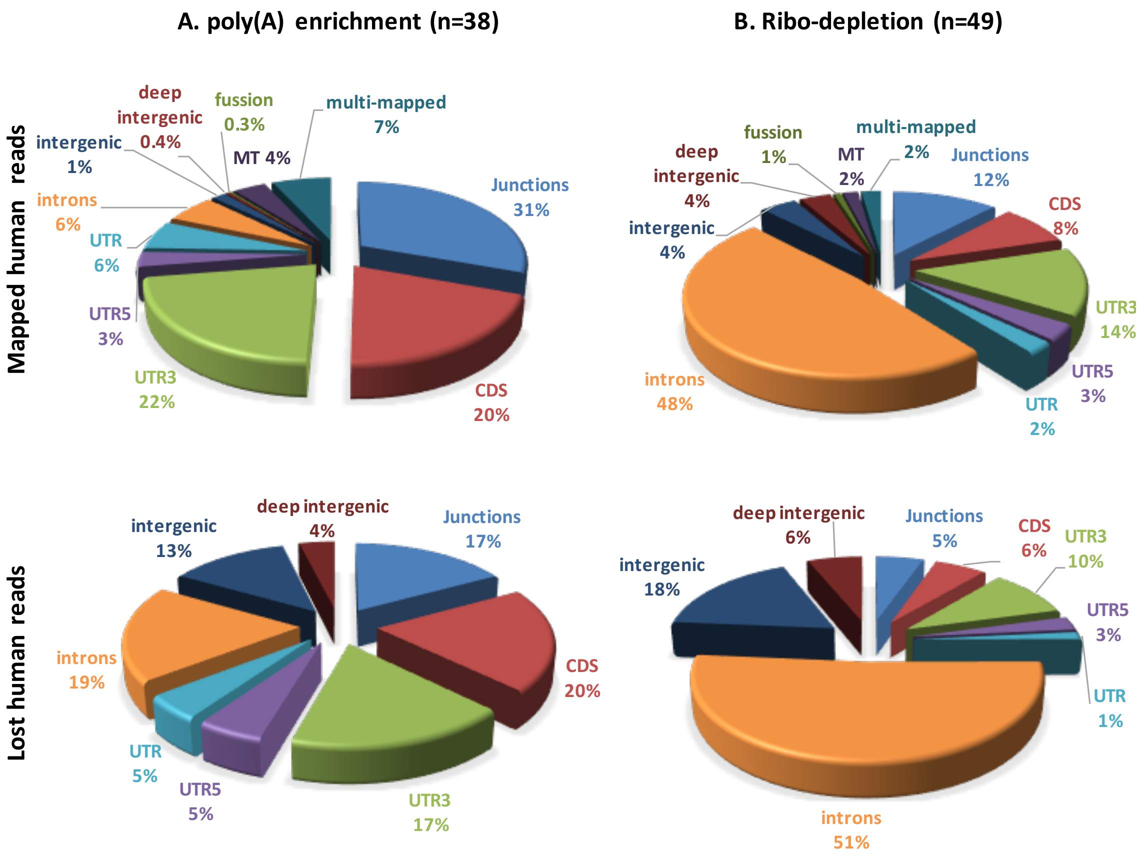

Supplemental Methods Figure SM4. Genomic profile of mapped and lost human reads across poly(A) enrichment and ribo-depletion libraries.

(A) RNA-Seq samples were prepared by poly(A) enrichment protocol (n=38). (B) RNA-Seq samples were prepared by ribo-depletion protocol (n=49). Mapped human reads are identified as RNA-Seq reads that mapped to the human reference genome and transcriptome (ENSEMBL hg19 build, ENSEMBL GRCh37 transcriptome) via tophat2. Lost human reads are unmapped RNA-Seq reads that aligned to the human reference genome and transcriptome (ENSEMBL hg19 build, ENSEMBL GRCh37 transcriptome) via more sensitive Megablast alignment. Single alignment is reported for each read by Megablast. ROP categorizes the reads into genomic categories based on the compatibility of each

read from the pair with the features defined by the Ensembl gene annotations. Percentages are calculated as a fraction of reads from a category from the total number of mapped or lost human reads. Junction read is defined as a read spanning exon-exon boundary; CDS, UTR3, UTR5: reads overlapping CDS, UTR3 or UTR5 region; UTR: reads simultaneously overlapping UTR3 and UTR5 regions; intronic: reads overlapping intronic regions; intergenic: reads mapped within the proximity of 1Kb from the gene boundaries; deep intergenic: reads mapped beyond the proximity of 1Kb from the gene boundaries; MT: mitochondrial reads; multi-mapped: reads mapped to multiple locations of the human genome; fusion: reads from the read pair mapped to different chromosomes.

Genomic profile across tissue types and library preparation methods in S1. Genomic Profile is obtained based on both mapped and lost human RNA-Seq reads.

A. Genomic profile obtained based on mapped RNA-Seq reads. Mapped human reads are identified as the RNA-Seq reads mapped to the reference genome and transcriptome (ENSEMBL hg19 build, ENSEMBL GRCh37 transcripome) via tophat2.

Tissue	Whole blood	Nasal epithelium	Lung epithelium
N	19	19	49
Library preparation method	poly(A) enrichment	poly(A) enrichment	ribo-depletion
Splice junction reads, %*, mean (std)	23.3% (3.3%)	29.8% (2.2%)	10.0% (3.3%)
CDS reads %, mean (std)	18.0% (3.1%)	16.9% (1.3%)	6.9% (2.0%)
UTR3 reads %, mean (std)	15.6% (3.1%)	22.5% (1.7%)	11.4% (2.5)
UTR5 reads %, mean (std)	3.2% (0.7%)	2.2% (0.3%)	2.6% (0.7%)
UTR** reads %, mean (std)	4.3% (0.8%)	5.9% (0.5%)	1.9% (0.6%)
Intronic reads %, mean (std)	5.6% (1.6%)	4.4% (0.8%)	39.4% (6.5%)
Proximate inter-genic*** reads %, mean (std)	1.2% (0.6%)	1.5% (0.6%)	3.3% (0.4%)
Deep inter-genic reads**** %, mean (std)	0.3% (0.1%)	0.3% (0.1%)	2.8% (0.9%)
Mitochondrial (MT) reads %*, mean (std)	2.3% (1.0%)	4.3% (1.3%)	1.5% (1.8%)
Milti-mapped reads %, mean (std)	10.6% (2.4%)	1.9% (0.2%)	1.9% (0.5%)
Fusion reads %, mean (std)	0.2% (0.1%)	0.4 % (0.1%)	0.7% (0.2%)

B. Genomic profile obtained based on lost human reads. Lost human reads are the unmapped RNA-Seq reads that aligned to the human reference genome and transcriptome (ENSEMBL hg19 build, ENSEMBL GRCh37 transcripome) via more sensitive Megablast alignment.

Tissue	Whole blood	Nasal epithelium	Lung epithelium
N	19	19	49
Library preparation method	poly(A) enrichment	poly(A) enrichment	ribo-depletion
Splice junction reads, %*, mean (std)	1.5% (0.5%)	0.7% (0.1%)	0.6% (0.2%)
CDS reads %, mean (std)	1.9% (0.7%)	0.7% (0.1%)	0.7% (0.2%)
UTR3 reads %, mean (std)	1.3% (0.3%)	0.9% (0.1%)	1.1% (0.2%)
UTR5 reads %, mean (std)	0.4% (0.1%)	0.2% (0.03%)	0.3% (0.1%)
UTR** reads %, mean (std)	0.4% (0.1%)	0.2% (0.1%)	0.2% (0.1%)
Intronic reads %, mean (std)	1.0% (0.4%)	1.3% (1.1%)	5.9% (3.1%)
Proximate inter-genic*** reads %, mean (std)	0.6% (0.4%)	1.0% (1.1%)	2.1% (2.5%)
Deep inter-genic reads**** %, mean (std)	0.2% (0.1%)	0.3% (0.3%)	0.7% (0.4%)
Mitochondrial (MT) reads %*, mean (std)	0.0% (0.0%)	0.0% (0.0%)	0.0% (0.0%)

Notes :
* percentage from the total number of reads are reported
** reads simultaneously overlapping UTR3 and UTR5 regions
*** mapped with the 1K proximity from gene boundaries
**** mapped further than 1K from the gene boundaries

Repeat profile across tissues types and library preparation methods.

Repeat profile is based on both mapped and lost repeat reads.

A. Repeat profile obtained based on mapped RNA-Seq reads. Mapped reads were categorized based on the overlap with the repeat instances prepared from RepeatMasker annotation (Repeatmasker v3.3, Repeat Library 20120124).

Tissue	Whole blood	Nasal epithelium	Lung epithelium
N	19	19	49
Library preparation method	poly(A) enrichment	poly(A) enrichment	ribo-depletion
L1, %*, mean	0.4%	0.5%	5.5%
L2, %, mean	0.2%	0.2%	1.0%
CR1, %, mean	0.02%	0.01%	0.1%
Alu, %, mean	1.0%	1.0%	2.5%
MIR, %, mean	0.1%	0.1%	0.6%
ERVL-MaLR, %, mean	0.2%	0.2%	1.1%
ERV1, %, mean	0.2%	0.2%	0.8%
ERVK, %, mean	0.0%	0.0%	0.1%
ERVL, %, mean	0.1%	0.1%	0.5%
RNA, %, mean	0.0%	0.0%	0.2%
hAT-Charlie, %, mean	0.1%	0.1%	0.4%
TcMar-Tigger, %, mean	0.04%	0.1%	0.5%
Others, %, mean	0.05%	0.1%	0.3%

* Percentage from the total number of reads

B. Repeat profile obtained based on lost repeat reads. Lost human reads are the unmapped RNA-Seq reads that aligned to human reference genome and transcriptome (ENSEMBL hg19 build, ENSEMBL GRCh37 transcripome) via more sensitive Megablast alignment.

Tissue	Whole blood	Nasal epithelium	Lung epithelium
N	19	19	49
Library preparation method	poly(A) enrichment	poly(A) enrichment	ribo-depletion
%, mean*			
hAT, mean	0.0001%	0.0004%	0.0000%
TcMar-Mariner, mean	0.0001%	0.0005%	0.0001%
TcMar-Tigger, mean	0.0001%	0.0015%	0.0001%
L1, mean	0.0045%	0.1409%	0.0048%
ERVK, mean	0.0002%	0.0026%	0.0001%
ERV, mean	0.0017%	0.0082%	0.0014%
ERV1, mean	0.0025%	0.0106%	0.0016%
ERVL, mean	0.0000%	0.0014%	0.0000%
Satellite, mean	0.0001%	0.0006%	0.0000%
Alu, mean	0.0495%	0.0896%	0.0382%
Deu, mean	0.0001%	0.0024%	0.0001%
Others, mean	0.0051%	0.0072%	0.0025%

*Percentage from the total number of reads

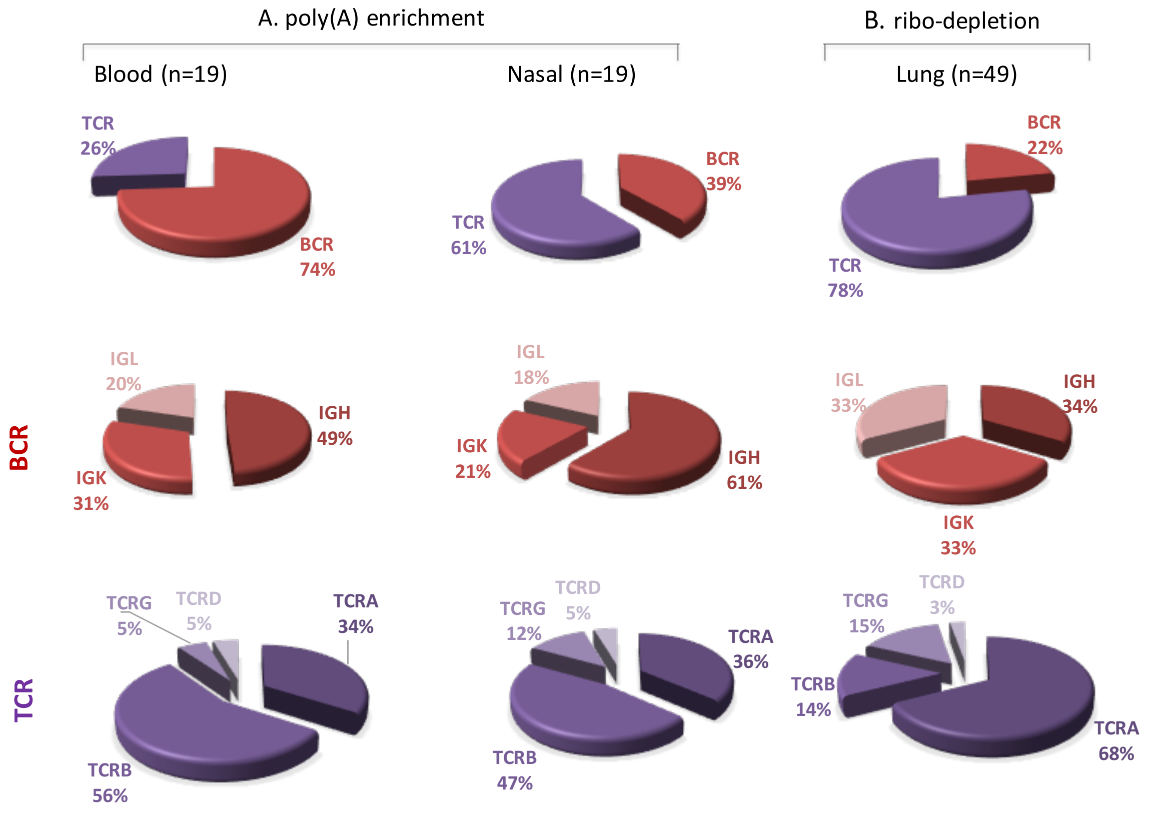

Supplemental Methods Figure SM5.. **Percentage of immune reads mapped to B-cell receptor (BCR) and T-cell receptor (TCR) loci.**

(A) RNA-Seq samples were prepared by poly(A) enrichment protocol (whole blood and nasal epithelium). (B) RNA-Seq samples were prepared by ribo-depletion protocol (lung epithelium). Immune reads that are entirely mapped to BCR and TCR genes are identified by tophat2. Immune reads with extensive somatic hyper mutations (SHM) and reads arising from V(D)J recombination are identified by IgBLAST. Blood samples show a larger fraction of reads mapped to BCR locus, while nasal and lung epithelium samples show a larger fraction of reads mapped to TCR locus. BCR are composed of heavy (IGH) and light chains. Among the reads mapped to BCR locus, the number of reads mapped to

immunoglobulin heavy locus (IGH), immunoglobulin kappa locus (IGK), and immunoglobulin lambda locus (IGL) is determined. Among the reads mapped to TCR locus, the number of reads mapped to T cell receptor alpha locus (TCRA), T cell receptor beta locus (TCRB), T cell receptor gamma locus (TCRG), and T cell receptor delta locus (TCRD) is determined.

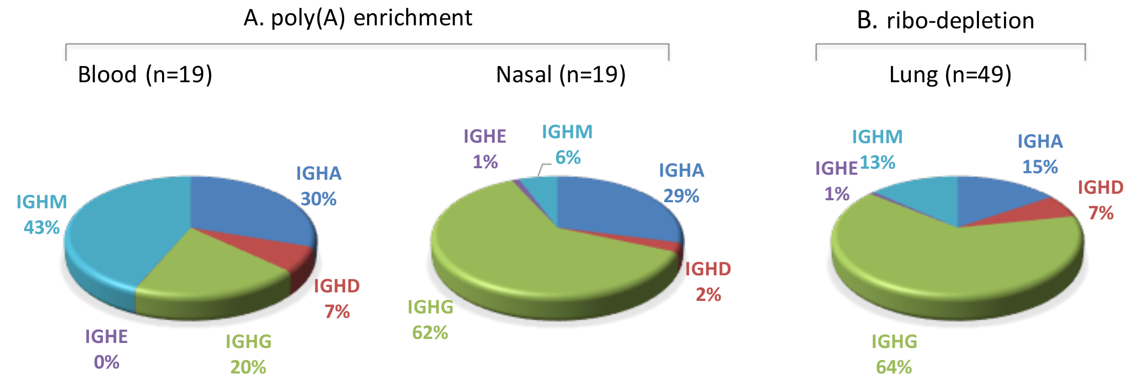

Supplemental Methods Figure SM6. Percentage of immune reads mapped to genes encoding the constant region of immunoglobulin heavy locus (IGH).

(A) RNA-Seq samples were prepared by poly(A) enrichment protocol (whole blood and nasal epithelium). (B) RNA-Seq samples were prepared by ribo-depletion protocol (lung epithelium). Immune reads that are entirely mapped to IGHA (Immunoglobulin Heavy Constant Alpha), IGHD (Immunoglobulin Heavy Constant Delta), IGHG (Immunoglobulin Heavy Constant Gamma), IGHE (Immunoglobulin Heavy Constant Epsilon), and IGHM (Immunoglobulin Heavy Constant Mu) are identified by tophat2.

Number of RNA-Seq reads mapped to BCR and TCR genes (immune reads).

Reads entirely mapped to BCR and TCR genes are identified by Tophat2. Reads with extensive somatic hyper mutations (SHM) and reads arising from V(D)J recombination are identified by IgBLAST.

Tissue	Whole blood	Nasal epithelium	Lung epithelium
N	19	19	49
Library preparation method	poly(A) enrichment	poly(A) enrichment	ribo-depletion
Number of immune reads (tophat2), RPM, mean	4805	107	16
Number of immune reads (IgBlast), RPM, mean	270	7	1
Total number of immune reads , RPM, mean	5075	114	17

RPM : reads per million

List of software tools used:

Tophat2 v.2.0.13 - http://ccb.jhu.edu/software/tophat/index.shtml

STAR v2.5.2b - https://github.com/alexdobin/STAR

Bowtie v.0.12.9 - http://bowtie-bio.sourceforge.net/index.shtml

Bowtie2 v.2.2.9 - http://bowtie-bio.sourceforge.net/bowtie2/index.shtml

Samtools v.0.1.18 - http://www.htslib.org/

Bamtools v.2.3.0 - https://github.com/pezmaster31/bamtools

FASTX-Toolkit v.0.0.13 - http://hannonlab.cshl.edu/fastx_toolkit/

SEQLEAN v(seqclean-x86_64) - http://sourceforge.net/projects/seqclean/files/

BLAST+ v.2.2.30 - ftp://ftp.ncbi.nlm.nih.gov/blast/executables/blast+/LATEST/

IgBLAST v.1.4.0- http://www.ncbi.nlm.nih.gov/IgBLAST/

TopHat-Fusion v.2.0.13- http://ccb.jhu.edu/software/tophat/fusion_index.shtml

circExplorer2 v.2.2.4 - http://circexplorer2.readthedocs.io/

MetaPhlAn2 v.2.0 - http://huttenhower.sph.harvard.edu/metaphlan

HTSeq v.0.6.1 - http://www-huber.embl.de/users/anders/HTSeq/

Preseq v 2.0- http://smithlabresearch.org/software/preseq/

Quicksect v.0.0.2 - https://github.com/brentp/quicksect

Databases

Ensembl hg19 - http://www.ensembl.org/Homo_sapiens/Info/Index

Human ribosomal DNA complete repeating unit - http://www.ncbi.nlm.nih.gov/nuccore/U13369

GTF formatted file for repeat annotations- http://labshare.cshl.edu/shares/mhammelllab/www-data/TEToolkit/TE_GTF/hg19_rmsk_TE.gtf.gz

Repeat elements (*RepBase20.07*) – http://www.girinst.org/repbase/

V(D)J genes of *B and T cell receptor* - http://www.imgt.org/vquest/refseqh.html#V-D-J-C-sets

Database of viral genomes: http://ftp.ncbi.nlm.nih.gov/genomes/Viruses

Database of bacterial genomes: http://ftp.ncbi.nlm.nih.gov/genomes/Bacteria/

Database of eukaryotic pathogens - http://eupathdb.org/eupathdb/

References:

Adiconis, X., Borges-Rivera, D., Satija, R., DeLuca, D. S., Busby, M. A., Berlin, A. M., … others. (2013). Comparative analysis of RNA sequencing methods for degraded or low-input samples. *Nature Methods*, *10*(7), 623–629.

Anders, S., Pyl, P. T., & Huber, W. (2014). HTSeq--A Python framework to work with high-throughput sequencing data. *Bioinformatics*, btu638.

Andrews, S. (2010). FastQC: A quality control tool for high throughput sequence data. Retrieved from http://www.bioinformatics.babraham.ac.uk/projects/fastqc/

Ardlie, K. G., Deluca, D. S., Segrè, A. V, Sullivan, T. J., Young, T. R., Gelfand, E. T., … others. (2015). The Genotype-Tissue Expression (GTEx) pilot analysis: Multitissue gene regulation in humans. *Science*, *348*(6235), 648–660.

Beck, J. M., Young, V. B., & Huffnagle, G. B. (2012). The microbiome of the lung. *Translational Research : The Journal of Laboratory and Clinical Medicine*, *160*(4), 258–66. https://doi.org/10.1016/j.trsl.2012.02.005

Blachly, J. S., Ruppert, A. S., Zhao, W., Long, S., Flynn, J., Flinn, I., … others. (2015). Immunoglobulin transcript sequence and somatic hypermutation computation from unselected RNA-seq reads in chronic lymphocytic leukemia. *Proceedings of the National Academy of Sciences*, *112*(14), 4322–4327.

Bunge, J., & Fitzpatrick, M. (1993). Estimating the number of species: a review. *Journal of the American Statistical Association*, *88*(421), 364–373.

Camacho, C., Coulouris, G., Avagyan, V., Ma, N., Papadopoulos, J., Bealer, K., & Madden, T. L. (2009). BLAST+: architecture and applications. *BMC Bioinformatics*, *10*(1), 421.

Carrara, M., Beccuti, M., Cavallo, F., Donatelli, S., Lazzarato, F., Cordero, F., & Calogero, R. A. (2013). State of art fusion-finder algorithms are suitable to detect transcription-induced chimeras in normal tissues? *BMC Bioinformatics*, *14*(7), 1.

Chuang, T.-J., Wu, C.-S., Chen, C.-Y., Hung, L.-Y., Chiang, T.-W., & Yang, M.-Y. (2015). NCLscan: accurate identification of non-co-linear transcripts (fusion, trans-splicing and circular RNA) with a good balance between sensitivity and precision. *Nucleic Acids Research*, gkv1013.

Cloonan, N., Forrest, A. R. R., Kolle, G., Gardiner, B. B. A., Faulkner, G. J., Brown, M. K., … others. (2008). Stem cell transcriptome profiling via massive-scale mRNA sequencing. *Nature Methods*, *5*(7), 613–619.

Colwell, R. K., & Coddington, J. A. (1994). Estimating terrestrial biodiversity through extrapolation. *Philosophical Transactions of the Royal Society B: Biological Sciences*, *345*(1311), 101–118.

Criscione, S. W., Zhang, Y., Thompson, W., Sedivy, J. M., & Neretti, N. (2014). Transcriptional landscape of repetitive elements in normal and cancer human cells. *BMC Genomics*, *15*(1), 583. https://doi.org/10.1186/1471-2164-15-583

Daley, T. P. (2014). *Non-parametric Models for Large Capture-recapture Experiments with Applications to DNA Sequencing*. University of Southern California.

Daley, T., & Smith, A. D. (2013). Predicting the molecular complexity of sequencing libraries. *Nature Methods*, *10*(4), 325–327.

Deng, C., Daley, T., & Smith, A. D. (n.d.). Applications of species accumulation curves in large-scale biological data analysis. *Journal of Quantitative Biology*.

Engström, P. G., Steijger, T., Sipos, B., Grant, G. R., Kahles, A., Rätsch, G., … others. (2013). Systematic evaluation of spliced alignment programs for RNA-seq data. *Nature Methods*, *10*(12), 1185–1191.

Favaro, S., Lijoi, A., & Prünster, I. (2012). A new estimator of the discovery probability. *Biometrics*, *68*(4), 1188–1196.

Good, I. J. (1953). The population frequencies of species and the estimation of population parameters. *Biometrika*, *40*(3–4), 237–264.

Grabherr, M. G., Haas, B. J., Yassour, M., Levin, J. Z., Thompson, D. A., Amit, I., … Regev, A. (2011). Full-length transcriptome assembly from RNA-Seq data without a reference genome. *Nature Biotechnology*, *29*(7), 644–52. https://doi.org/10.1038/nbt.1883

Hach, F., Hormozdiari, F., Alkan, C., Hormozdiari, F., Birol, I., Eichler, E. E., & Sahinalp, S. C. (2010). mrsFAST: a cache-oblivious algorithm for short-read mapping. *Nature Methods*, *7*(8), 576–577.

Hansen, T. B., Venø, M. T., Damgaard, C. K., & Kjems, J. (2015). Comparison of circular RNA prediction tools. *Nucleic Acids Research* . https://doi.org/10.1093/nar/gkv1458

Inman, C. F., Murray, T. Z., Bailey, M., & Cose, S. (2012). Most B cells in non-lymphoid tissues are naïve. *Immunology and Cell Biology*, *90*(2), 235–242. https://doi.org/10.1038/icb.2011.35

Jeck, W. R., & Sharpless, N. E. (2014). Detecting and characterizing circular RNAs. *Nature Biotechnology*, *32*(5), 453–61. https://doi.org/10.1038/nbt.2890

Jin, Y., Tam, O. H., Paniagua, E., & Hammell, M. (2015). TEtranscripts: a package for including transposable elements in differential expression analysis of RNA-seq datasets. *Bioinformatics*, btv422.

Kim, D., Pertea, G., Trapnell, C., Pimentel, H., Kelley, R., & Salzberg, S. L. (2013). TopHat2: accurate alignment of transcriptomes in the presence of insertions, deletions and gene fusions. *Genome Biology*, *14*(4), R36. https://doi.org/10.1186/gb-2013-14-4-r36

Kostic, A. D., Ojesina, A. I., Pedamallu, C. S., Jung, J., Verhaak, R. G. W., Getz, G., & Meyerson, M. (2011). PathSeq: software to identify or discover microbes by deep sequencing of human tissue. *Nature Biotechnology*, *29*(5), 393–396.

Li, S., Tighe, S. W., Nicolet, C. M., Grove, D., Levy, S., Farmerie, W., … others. (2014). Multi-platform assessment of transcriptome profiling using RNA-seq in the ABRF next-generation sequencing study, *32*(9), 915–925. https://doi.org/10.1038/nbt.2972

Mao, C. X. (2004). Predicting the conditional probability of discovering a new class. *Journal of the American Statistical Association*, *99*(468).

Melé, M., Ferreira, P. G., Reverter, F., DeLuca, D. S., Monlong, J., Sammeth, M., … others. (2015). The human transcriptome across tissues and individuals. *Science*, *348*(6235), 660–665.

Mihaela Pertea, J. T. M. S. L. S. (2015). StringTie enables improved reconstruction of a transcriptome from RNA-seq reads. *Nature Biotechnology*, *33*, 290–295. https://doi.org/10.1038/nbt.3122

Nicolae, M., Mangul, S., Mandoiu, I. I., & Zelikovsky, A. (2011). Estimation of alternative splicing isoform frequencies from RNA-Seq data. *Algorithms for Molecular Biology*, *6*(1), 9.

Ozsolak, F., & Milos, P. M. (2011). RNA sequencing: advances, challenges and opportunities. *Nature Reviews. Genetics*, *12*(2), 87–98. https://doi.org/10.1038/nrg2934

Perucheon, S., Chaoul, N., Burelout, C., Delache, B., Brochard, P., Laurent, P., … Richard, Y. (2009). Tissue-specific B-cell dysfunction and generalized memory B-cell loss during acute SIV infection. *PLoS ONE*, *4*(6), e5966. https://doi.org/10.1371/journal.pone.0005966

Porath, H. T., Carmi, S., & Levanon, E. Y. (2014). A genome-wide map of hyper-edited RNA reveals numerous new sites. *Nature Communications*, *5*, 4726. https://doi.org/10.1038/ncomms5726

Salter, S. J., Cox, M. J., Turek, E. M., Calus, S. T., Cookson, W. O., Moffatt, M. F., … Walker, A. W. (2014). Reagent and laboratory contamination can critically impact sequence-based microbiome analyses. *BMC Biology*, *12*(1), 87.

Seqc/Maqc-Iii Consortium. (2014). A comprehensive assessment of RNA-seq accuracy, reproducibility and information content by the Sequencing Quality Control Consortium. *Nature Biotechnology*, *32*(9), 903–914. https://doi.org/10.1038/nbt.2957

Siragusa, E., Weese, D., & Reinert, K. (2013). Fast and accurate read mapping with approximate seeds and multiple backtracking. *Nucleic Acids Research*, *41*(7), e78--e78.

Spreafico, R., Rossetti, M., van Loosdregt, J., Wallace, C. A., Massa, M., Magni-Manzoni, S., … Albani, S. (2016). A circulating reservoir of pathogenic-like CD4+ T cells shares a genetic and phenotypic signature with the inflamed synovial micro-environment. *Annals of the Rheumatic Diseases*, *75*(2), 459–465.

Strauli, N., & Hernandez, R. (2015). Statistical Inference of a Convergent Antibody Repertoire Response to Influenza Vaccine. *bioRxiv*, 25098.

Sultan, M., Schulz, M. H., Richard, H., Magen, A., Klingenhoff, A., Scherf, M., … others. (2008). A global view of gene activity and alternative splicing by deep sequencing of the human transcriptome. *Science*, *321*(5891), 956–960.

Tang, F., Barbacioru, C., Wang, Y., Nordman, E., Lee, C., Xu, N., … others. (2009). mRNA-Seq whole-transcriptome analysis of a single cell. *Nature Methods*, *6*(5), 377–382.

Tarailo-Graovac, M., & Chen, N. (2009). Using RepeatMasker to identify repetitive elements in genomic sequences. *Current Protocols in Bioinformatics*, 4–10.

Trapnell, C., Williams, B. a, Pertea, G., Mortazavi, A., Kwan, G., van Baren, M. J., … Pachter, L. (2010). Transcript assembly and quantification by RNA-Seq reveals unannotated transcripts and isoform switching during cell differentiation. *Nature Biotechnology*, *28*(5), 511–515. https://doi.org/10.1038/nbt.1621

Truong, D. T., Franzosa, E. A., Tickle, T. L., Scholz, M., Weingart, G., Pasolli, E., … Segata, N. (2015). MetaPhlAn2 for enhanced metagenomic taxonomic profiling. *Nature Methods*, *12*(10), 902–903.

Wang, X.-S., Prensner, J. R., Chen, G., Cao, Q., Han, B., Dhanasekaran, S. M., … Chinnaiyan, A. M. (2009). An integrative approach to reveal driver gene fusions from paired-end sequencing data in cancer. *Nature Biotechnology*, *27*(11), 1005–11. https://doi.org/10.1038/nbt.1584

Wang, Z., Gerstein, M., & Snyder, M. (2009). RNA-Seq: a revolutionary tool for transcriptomics. *Nature Reviews Genetics*, *10*(1), 57–63.

Wu, C.-S., Yu, C.-Y., Chuang, C.-Y., Hsiao, M., Kao, C.-F., Kuo, H.-C., & Chuang, T.-J. (2014). Integrative transcriptome sequencing identifies trans-splicing events with important roles in human embryonic stem cell pluripotency. *Genome Research*, *24*(1), 25–36.

Yan, M., Pamp, S. J., Fukuyama, J., Hwang, P. H., Cho, D. Y., Holmes, S., & Relman, D. a. (2013). Nasal microenvironments and interspecific interactions influence nasal microbiota complexity and S. aureus carriage. *Cell Host and Microbe*, *14*(6), 631–640. https://doi.org/10.1016/j.chom.2013.11.005

Ye, J., Ma, N., Madden, T. L., & Ostell, J. M. (2013). IgBLAST: an immunoglobulin variable domain sequence analysis tool. *Nucleic Acids Research*, gkt382.

Zhang, X.-O., Dong, R., Zhang, Y., Zhang, J.-L., Luo, Z., Zhang, J., … Yang, L. (2016). Diverse alternative back-splicing and alternative splicing landscape of circular RNAs. *Genome Research*. https://doi.org/10.1101/gr.202895.115
