# Supplementary material for: ROP: dumpster diving in RNA-sequencing to find the source of 1 trillion reads across diverse adult human tissues
Source: Genome Biol. 2018 Feb 15;19:36. doi: 10.1186/s13059-018-1403-7 (PMC5857127; doi:10.1186/s13059-018-1403-7)
Supplement: Supplementary file 2 — Table S1. The effect of altering order of ROP step on the classification accuracy. Table S2. Concordance of targeted TCRB-Seq and ROP based on three TCGA samples from kidney renal clear cell carcinoma (KIRC). Table S4. RNA-seq datasets overview. Table S5. Genomic profile of unmapped reads reported for each dataset (S1, S2, S3). Table S6. Relative genomic abundance of microbial taxa at different levels of taxonomic classification after removal of reads with human origin (average over all samples of three tissues, performed for in-house RNA-Seqdata). Table S7. Genomic profile of unmapped reads across two SRA RNA-seq samples using ROP v1.0.8. Percentage for each category is calculated as a fraction from the total number of reads. (PDF 100 kb) [file 13059_2018_1403_MOESM2_ESM.pdf]

Supplemental Tables

Supplemental Table S1. The effect of altering order of ROP step on the classification accuracy. Each column represents a category from which reads were simulated. Each row represents a distribution of reads from the same class across ROP categories. The results are presented for both the default and the altered order of ROP steps. Reads were mapped with TopHat2. Order of steps is presented in column 1. (a) The default order of ROP steps: "lost human reads," "lost repeat reads," "immune reads," and "microbial reads." (b) Altered order of ROP steps: "lost repeat reads," lost human reads," "immune reads," and "microbial reads." (c) Altered order of ROP steps: "immune reads," lost human reads," "lost repeat reads," and "microbial reads." (d) Altered order of ROP steps: "microbial reads," "lost human reads," "lost repeat reads," and "immune reads." Percentages are calculated from total number of reads from each category. Reads were simulated as described here: https://github.com/smangul1/rop-project/wiki/Simulated-RNA-Seq-data-as-a-mixture. We used reference human transcript sequences to simulate transcriptomics reads. We used reference repeat sequences to simulate repeat reads. Immune transcripts were simulated as recombinations of V D and J segments with non-template insertions at the junction points. We have used microbiome sequences downloaded from NCBI to simulate the microbial reads.

(a)

Order of steps		transcriptomics reads	repeat reads	BCRs/TCRs	microbiome reads
	Total number of reads	1226	500	500	500
1	TopHat2	921	99	307	0
2	ROP step "lost human reads"	288	109	116	0
3	ROP step "lost repeat reads"	0	283	0	0
4	ROP step "immune(BCRs/TCRs)"	0	0	7	0
5	ROP step "microbiome reads"	0	0	0	500
	Unaccounted	5	9	70	0

(b)

Order of steps		transcriptomics reads	repeat reads	BCRs/TCRs	microbiome reads
	Total number of reads	1226	500	500	500
1	TopHat2	921	99	307	0

3	ROP step "lost human reads"	288	1	116	0
2	ROP step "lost repeat reads"	0	391	0	0
4	ROP step "immune(BCRs/TCRs)"	0	0	7	0
5	ROP step "microbiome reads"	0	0	0	500
	Unaccounted	5	9	70	0

(c)

Order of steps		transcriptomics reads	repeat reads	BCRs/TCRs	microbiome reads
	Total number of reads	1226	500	500	500
1	TopHat2	921	99	307	0

3	ROP step "lost human reads"	288	109	116	0
4	ROP step "lost repeat reads"	0	283	0	0
2	ROP step "immune(BCRs/TCRs)"	0	0	7	0
5	ROP step "microbiome reads"	0	0	0	500
	Unaccounted	5	9	70	0

(d)

Order of steps		transcriptomics reads	repeat reads	BCRs/TCRs	microbiome reads
	Total number of reads	1226	500	500	500
1	TopHat2	921	99	307	0
3	ROP step "lost human reads"	287	79	116	0

4	ROP step "lost repeat reads"	0	283	0	0
5	ROP step "immune(BCRs/TCRs)"	0	0	7	0
2	ROP step "microbiome reads"	1	30	0	500
	Unaccounted	5	9	70	0

Supplemental Table S2. Concordance of targeted TCRB-Seq and ROP based on three TCGA samples from kidney renal clear cell carcinoma (KIRC). The table presents the number of matching VJ recombinations reported by the immunoSEQ Analyzer (http://www.adaptivebiotech.com/) from TCRB-Seq and VJ recombinations detected by ROP from non-specific RNA-Seq data. The raw results and script used to process data are available at: https://github.com/smangul1/rop-project/ .

Sample name	VJ recombinations detected by TCRB-Seq	Vj recombinations detected by ROP	Matching VJ recombinations detected by both TCRB-Seq and ROP
TCGA-CZ-4862	54	4	4

TCGA-CZ-5463	53	1	1
TCGA-CZ-5985	53	2	2

Supplemental Table S3. The list of the 2000 SRA RNA-Seq samples used in the analysis. SRA samples were randomly selected from the Sequence Read Archive (SRA)

[Table S3 is provided as a separate file]

Supplemental Table S4. RNA-Seq datasets overview. In-house RNA-Seq data (n=86) from the peripheral blood, nasal, and large airway epithelium of asthmatic and control individuals (S1); (2) multi-tissue RNA-Seq data from Genotype-Tissue Expression (GTEx v6) from 53 human body sites [1] (n=8555) (S2); (3) randomly selected RNA-Seq samples from the Sequence Read Archive (SRA) (n=2000) (S3). Unless otherwise noted, we reported percentage of reads averaged across 3 datasets. For counting purposes, the pairing information of the reads is disregarded, and each read from a pair is counted separately.

Datasets	S1	S2	S3
Number of samples	87	8555	2000
Read length	100bp	76bp	25-100bp
Average number of reads per sample, (million reads)	88.8	54.6	90.2
Percentage of mapped reads (%)	83.8%	88.2%	77.2%

Supplemental Table S5. Genomic profile of unmapped reads reported for each dataset (S1, S2, S3). Percentage of unmapped reads for each category is calculated as a fraction from the total number of reads. Bars of the plot are not scaled. Human reads (black color) mapped to reference genome and transcriptome via TopHat2. (a) Low quality/low-complexity (light brown) and reads matching rRNA repeating unit (dark brown) were excluded. (b) Hyper-edited reads are captured by hyper-editing pipeline proposed in [2]. (c) ROP identifies lost human reads (red color) from unmapped reads using a more sensitive alignment. (d) ROP identifies lost repeat sequences (green color) by mapping unmapped reads onto the reference repeat sequences. (e) Reads arising from trans-spicing, gene fusion and circRNA events (orange color) are captured by a TopHat-Fusion and CIRCexplorer2 tools. (f) IgBLAST is used to identify reads spanning B and T cell receptor gene rearrangement in the variable domain (V(D)J recombinations) (violet color). (g) Microbial reads (blue color) are captured by mapping the reads onto the microbial reference genomes.

	S1	S2	S3	Averaged across 3 datasets

Mapped	83.2%	88.2%	77.2%	82.9%
Unmapped	17%	11.8%	23%	17.1%
Low quality reads	4.8%	7.0%	9%	7.0%
rRNA repeat	3.8%	0.1%	3%	2.4%
Lost human reads	6.0%	3.7%	8%	5.7%
Hyper-edited reads	0.02%	0.1%	0.1%	0.1%
Lost repeat reads	0.3%	0.1%	0.1%	0.2%
NCL RNA	0.3%	0.3%	0.4%	0.3%
V(D)J recombinations	0.01%	0.03%	0.01%	0.02%
Microbial reads	1.5%	0.5%	2.3%	1.4%
Unaccounted reads	0.18%	0.09%	0.10%	0.12%

Supplemental Table S6. Relative genomic abundance of microbial taxa at different levels of taxonomic classification after removal of reads with human origin (average over all samples of tissues). Taxonomic classification is performed using MetaPhlAn2, which is able to assign the filtered unmapped reads to the microbial marker genes.

Tissue	Whole blood	Nasal epithelium	Lung epithelium
N	19	19	49
Library preparation method	poly(A) enrichment	poly(A) enrichment	ribo-depletion
**Phylum**			
Proteobacteria	0.0%	0.9%	100.0%
Actinobacteria	0.0%	99.1%	0.0%
**Class**			
Betaproteobacteria	0.0%	0.5%	86.7%
Gammaproteobacteria	0.0%	0.5%	13.3%
Actinobacteria	0.0%	98.9%	0.0%
**Order**			
Burkholderiales	0.0%	0.0%	87.0%
Enterobacteriales	0.0%	0.0%	12.0%
Actinomycetales	0.0%	99.5%	0.0%
Pseudomonadales	0.0%	0.5%	1.0%

Supplemental Table S7. Genomic profile of unmapped reads across 2 SRA RNA-Seq samples using ROP v1.0.8. Percentage for each category is calculated as a fraction from the total number of reads.

sample	mapped reads	rRNA repeat	lost human reads	lost repeat elements	NCL RNAs	recombined BCR/TCRs	microbial reads	low quality reads	unaccounted reads
SRR3703207	95.3%	0.001%	1.1%	0.002%	0.04%	0.0004%	0.7%	2.6%	0.3%
SRR5831944	93.6%	0.003%	1.7%	0.0%	0.2%	0.0017%	2.0%	1.1%	1.4%
