# Supplementary material for: ROP: dumpster diving in RNA-sequencing to find the source of 1 trillion reads across diverse adult human tissues
Source: Genome Biol. 2018 Feb 15;19:36. doi: 10.1186/s13059-018-1403-7 (PMC5857127; doi:10.1186/s13059-018-1403-7)
Supplement: Supplementary file 4 — Figure S1. Edit distance of lost human reads. Figure S2. Profile of repeat elements based on repeat sequences inferred from mapped and unmapped reads (lost repeat reads). Figure S3. Profile of DNA repeats based on repeat sequences inferred from mapped and unmapped reads (lost repeat reads). Figure S4. Profile of SVA retrotransposons based on repeat sequences inferred from mapped and unmapped reads (lost repeat reads). Figure S5. Profile of repeat elements across poly(A) enrichment and ribodepletion libraries. Figure S6. Average number of SVA-F reads across GTEx tissues. Figure S7. Co-expression of Alu and L1 elements across GTEx tissues. Figure S8. Distribution 77 of hyper-edited reads. Figure S9. The sequence context of Figure S8. Figure S10. Distribution of NCL events across 10,641 samples. Figure S11. Number of NCL events across in-house tissues and library preparation protocols. Figure S12. Percentage of NCL reads across GTEx tissues (n = 54). Figure S13. An example of coverage plot of EBV virus. Figure S14. Number of VJ recombinations across GTEx human tissues for IGK chain. Figure S15. Number of VJ recombinations across GTEx human tissues for IGL chain. Figure S16. Association between microbial load and immune diversity. Figure S17. Combinatorial diversity of IGL locus differentiates disease status. Figure S18. Combinatorial diversity of TCRB locus differentiates disease status. Figure S19. Combinatorial diversity of TCRG locus differentiates disease status. Figure S20. The effect of RNA-seq aligner on the fraction of reads accounted by ROP. Figure S21. Relationship between the number of soft clipped RNA-seq reads (partially mapped reads) and the total number of reads. Figure S22. Number of the RNA-seq reads mapped to the human reference genome across five state-of-the-art RNA-seq aligners. (PDF 2344 kb) [file 13059_2018_1403_MOESM4_ESM.pdf]

Supplementary Figures

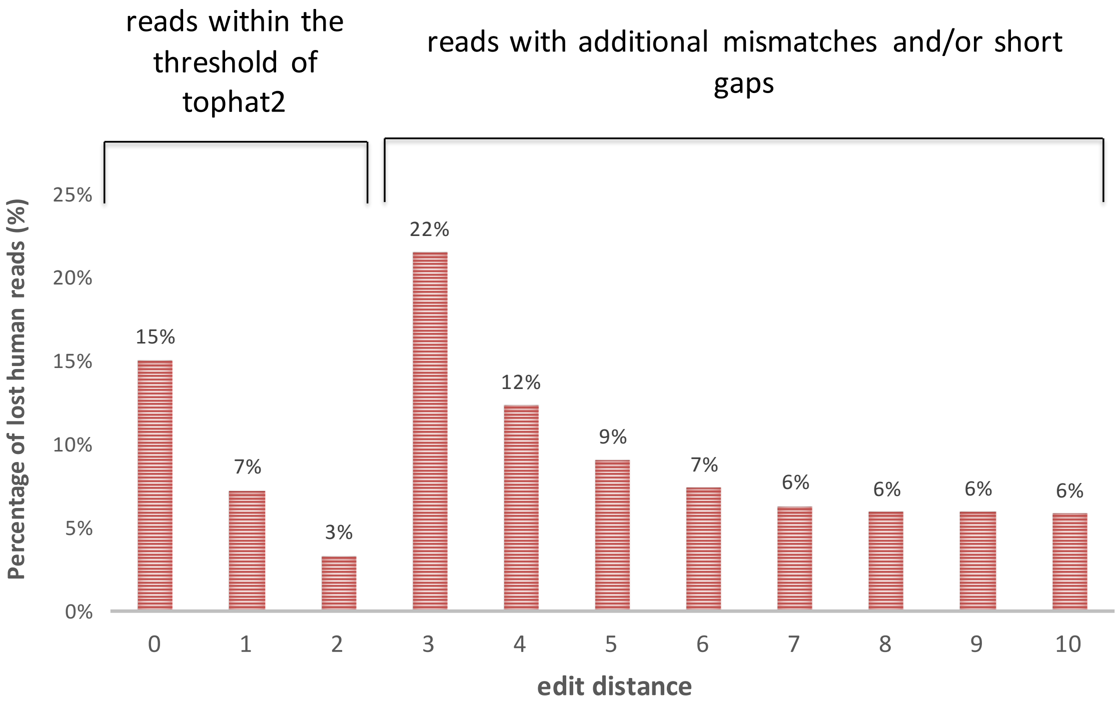

Supplemental Figure S1. Edit distance of lost human reads.

Unmapped reads were remapped to the human references using Megablast. Edit distance was calculated as the minimum number of operations required to transform a read sequence into the corresponding reference subsequence. Reads are grouped by edit distance with the transcriptome or the genome reference. The percentages are the averages across 10,641 samples.

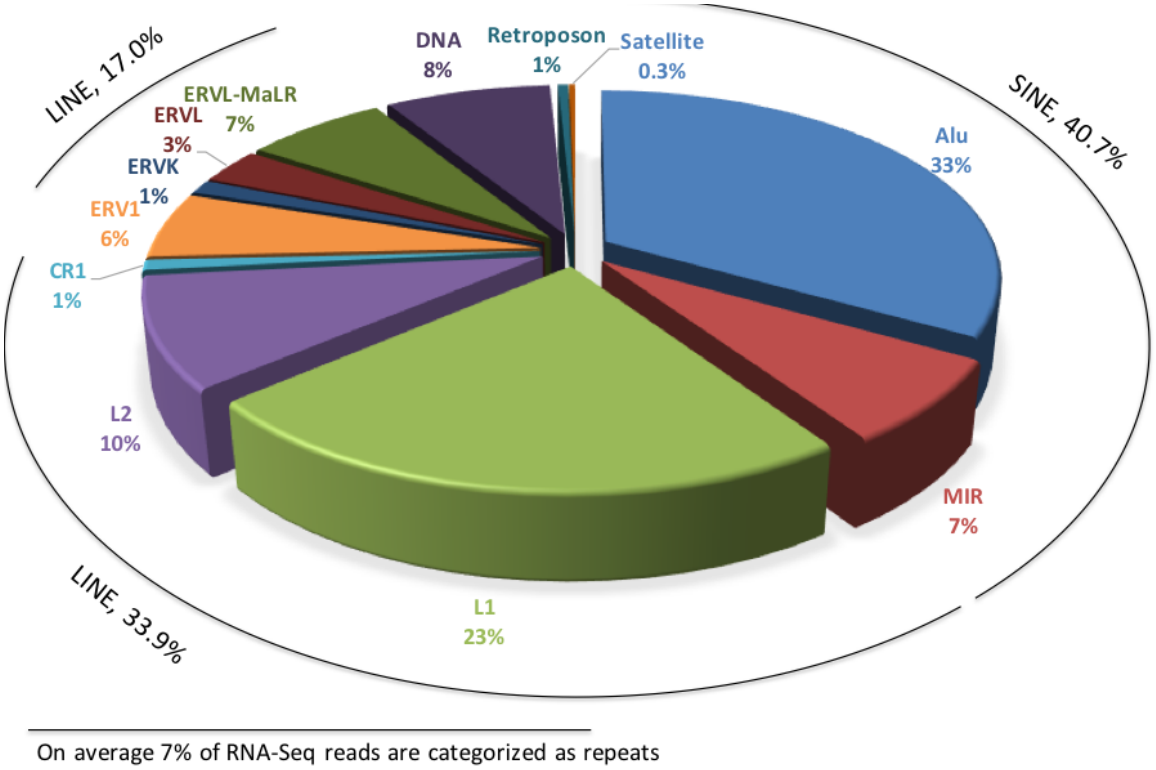

Supplemental Figure S2. Profile of repeat elements based on repeat sequences inferred from mapped and unmapped reads (lost repeat reads).

ROP identifies and categorizes repetitive sequences among the mapped and unmapped reads. Mapped reads were categorized based on the overlap with the repeat instances prepared from RepeatMasker annotation (RepeatMasker v3.3, Repeat Library 20120124). Lost repeat reads are unmapped RNA-Seq reads aligned onto the reference repeat sequences (prepared from Repbase v20.07). The percentages are the averages across 10,641 samples.

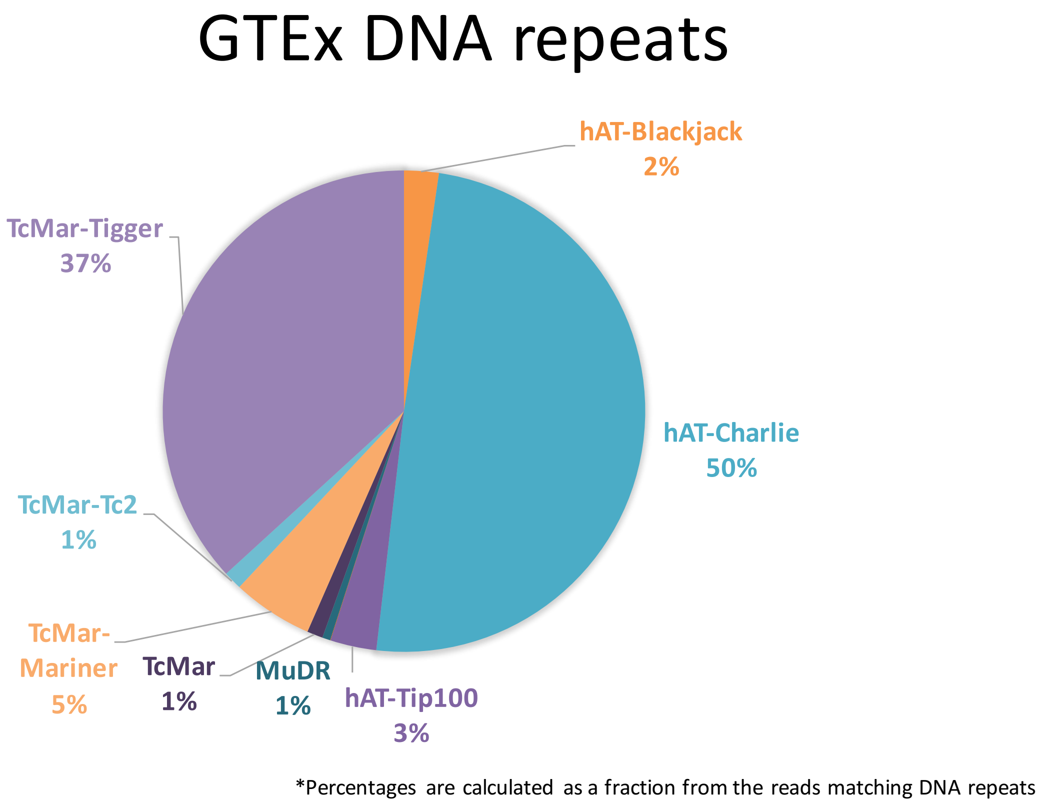

Supplemental Figure S3. Profile of DNA repeats based on repeat sequences inferred from mapped and unmapped reads (lost repeat reads).

ROP identifies and categorizes DNA repetitive sequences among the mapped and unmapped reads. Mapped reads were categorized based on the overlap with the repeat instances prepared from RepeatMasker annotation (RepeatMasker v3.3, Repeat Library 20120124). Lost repeat reads are unmapped RNA-Seq reads aligned onto the reference repeat sequences (prepared from Repbase v20.07). The percentages are the averages across 10,641 samples.

SINE–VNTR–*Alu* (SVA)

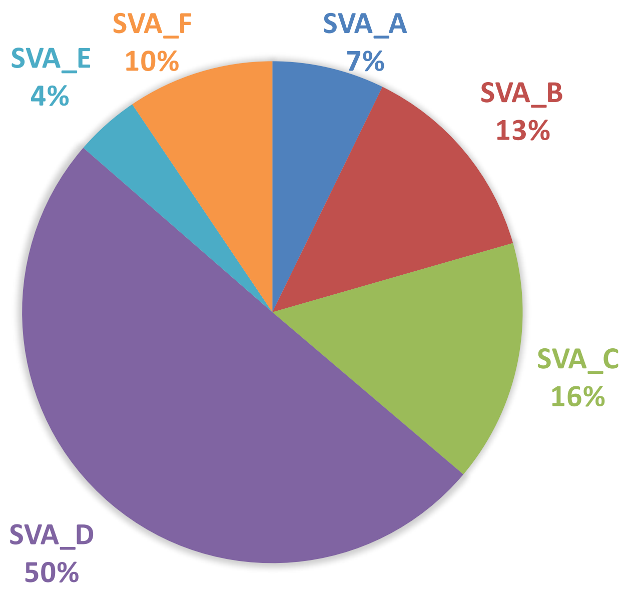

*Percentages are calculated as a fraction from the reads matching SVA Retroposons

Supplemental Figure S4. Profile of SVA retrotransposons based on repeat sequences inferred from mapped and unmapped reads (lost repeat reads). ROP identifies and categorizes SVA retrotransposons sequences among the mapped and unmapped reads. Mapped reads were categorized based on the overlap with the repeat instances prepared from RepeatMasker annotation (Repeatmasker v3.3, Repeat Library 20120124). Lost repeat reads are unmapped RNA-Seq reads aligned onto the reference repeat sequences (prepared from Repbase v20.07). The percentages are the averages across 10,641 samples.

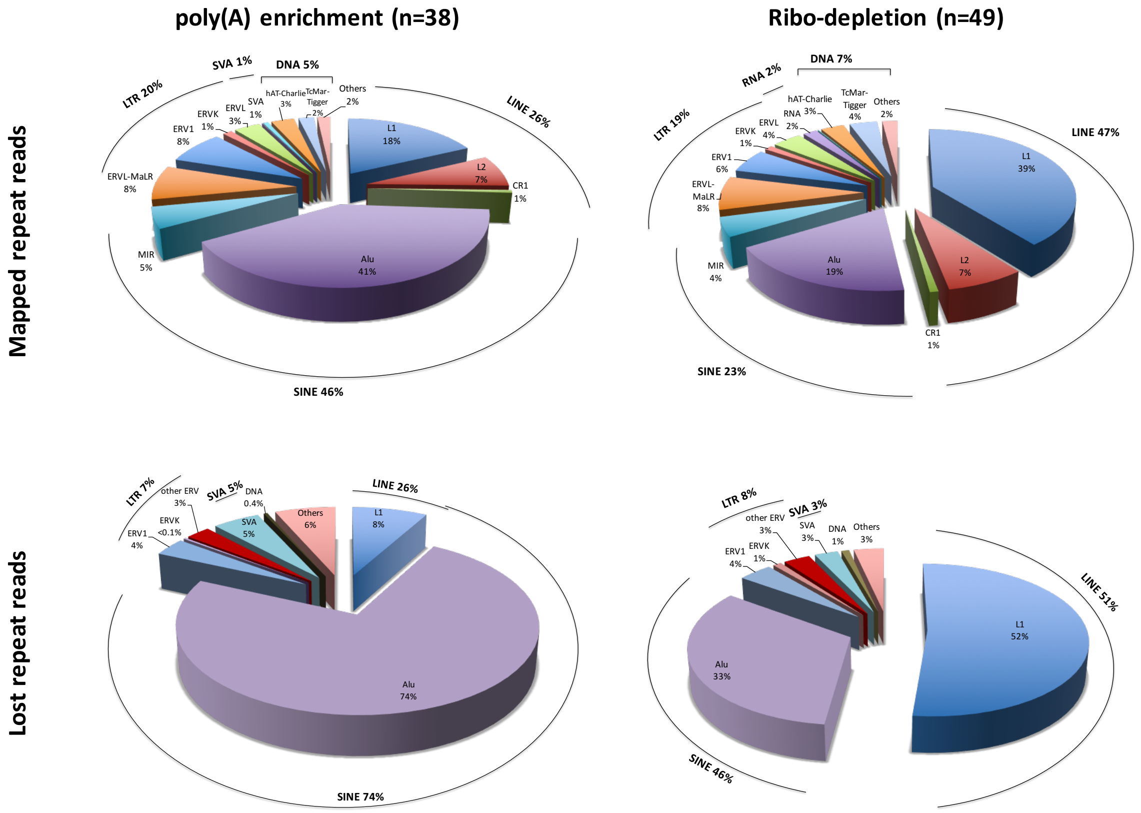

Supplemental Figure S5. Profile of repeat elements across poly(A) enrichment and ribo-depletion libraries. ROP identifies and categorizes repetitive sequences among the mapped and unmapped reads. RNA-Seq samples were prepared by poly(A) enrichment protocol (n=38) and ribo-depletion protocol (n=49). Mapped reads were categorized based on the overlap with the repeat instances prepared from RepeatMasker annotation (RepeatMasker v3.3, Repeat Library 20120124). Lost repeat reads are unmapped RNA-Seq reads aligned onto the reference repeat sequences (prepared from Repbase v20.07).

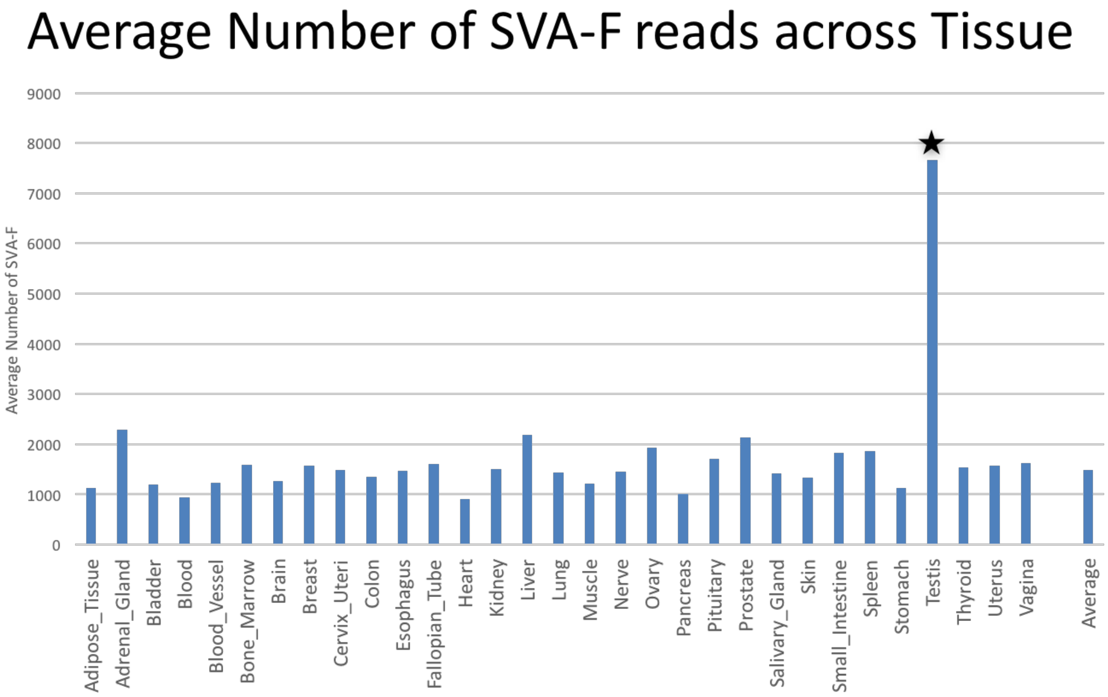

Supplemental Figure S6. Average number of SVA-F reads across GTEx tissues. ROP identifies and categorizes SVA retrotransposons sequences among the mapped and unmapped reads. Mapped reads were categorized based on the overlap with the repeat instances prepared from RepeatMasker annotation (RepeatMasker v3.3, Repeat Library 20120124). Lost repeat reads are unmapped RNA-Seq reads aligned onto the reference repeat sequences (prepared from Repbase v20.07). Among the GTEx tissues, *testis* showed significantly higher expression of SVA F retrotransposons compared to other tissues ($\mathbf{p = 2.46{\times}10^{-33}}$).

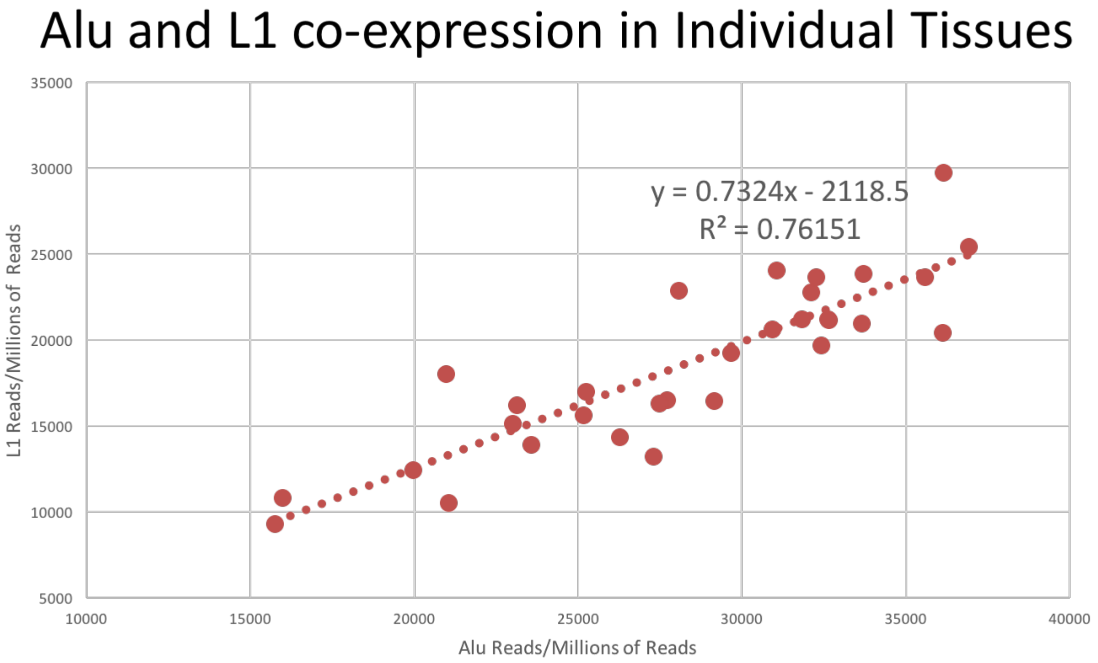

Supplemental Figure S7. Co-expression of Alu and L1 elements across GTEx tissues. ROP identifies and categorizes repetitive sequences among the mapped and unmapped reads. Mapped reads were categorized based on the overlap with the repeat instances prepared from RepeatMasker annotation (RepeatMasker v3.3, Repeat Library 20120124). Lost repeat reads are unmapped RNA-Seq reads aligned onto the reference repeat sequences (prepared from Repbase v20.07).

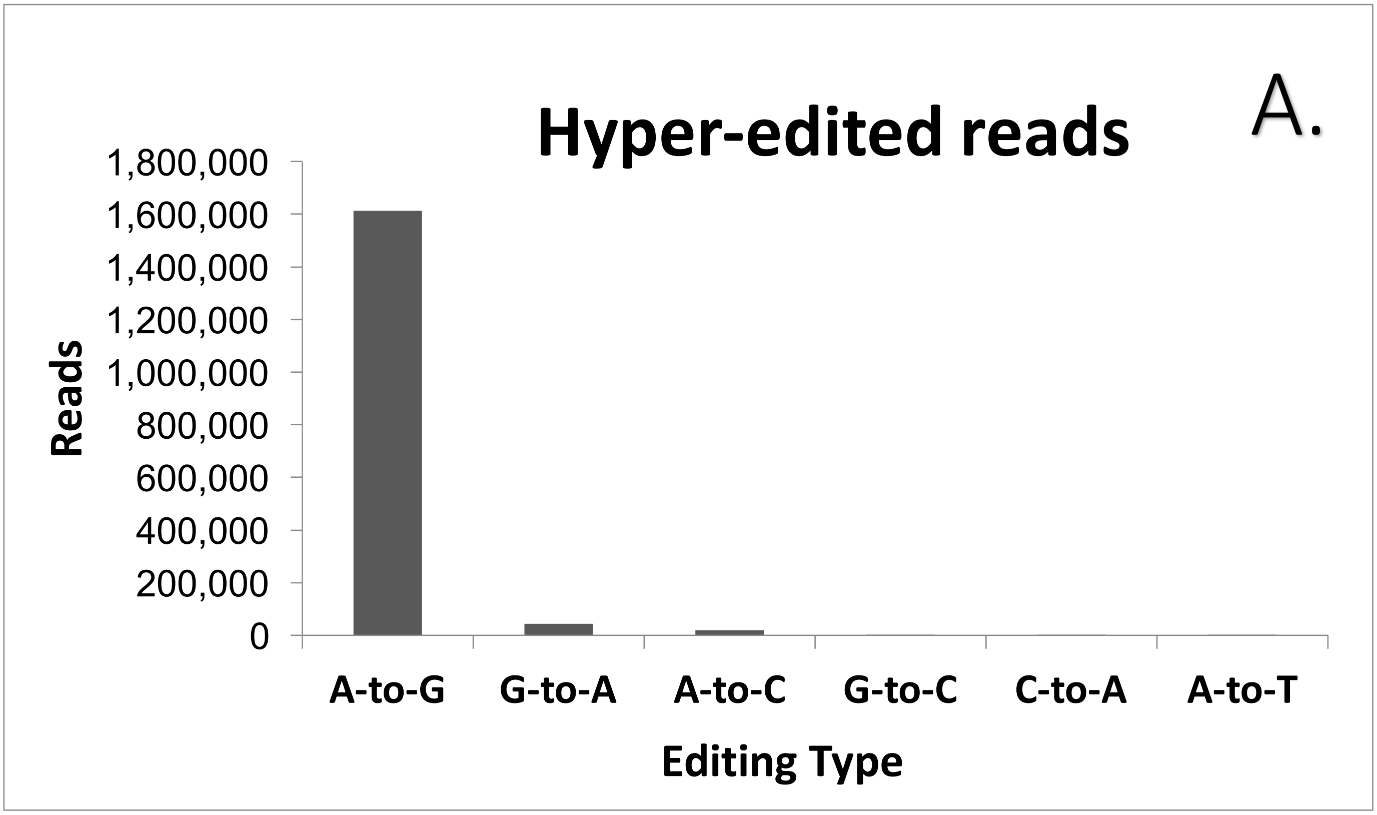
Hyper-edited reads
A.
1,800,000
1,600,000
1,400,000
1,200,000
1,000,000
800,000
600,000
400,000
200,000
0
Reads
A-to-G
G-to-A
A-to-C
G-to-C
C-to-A
A-to-T
Editing Type

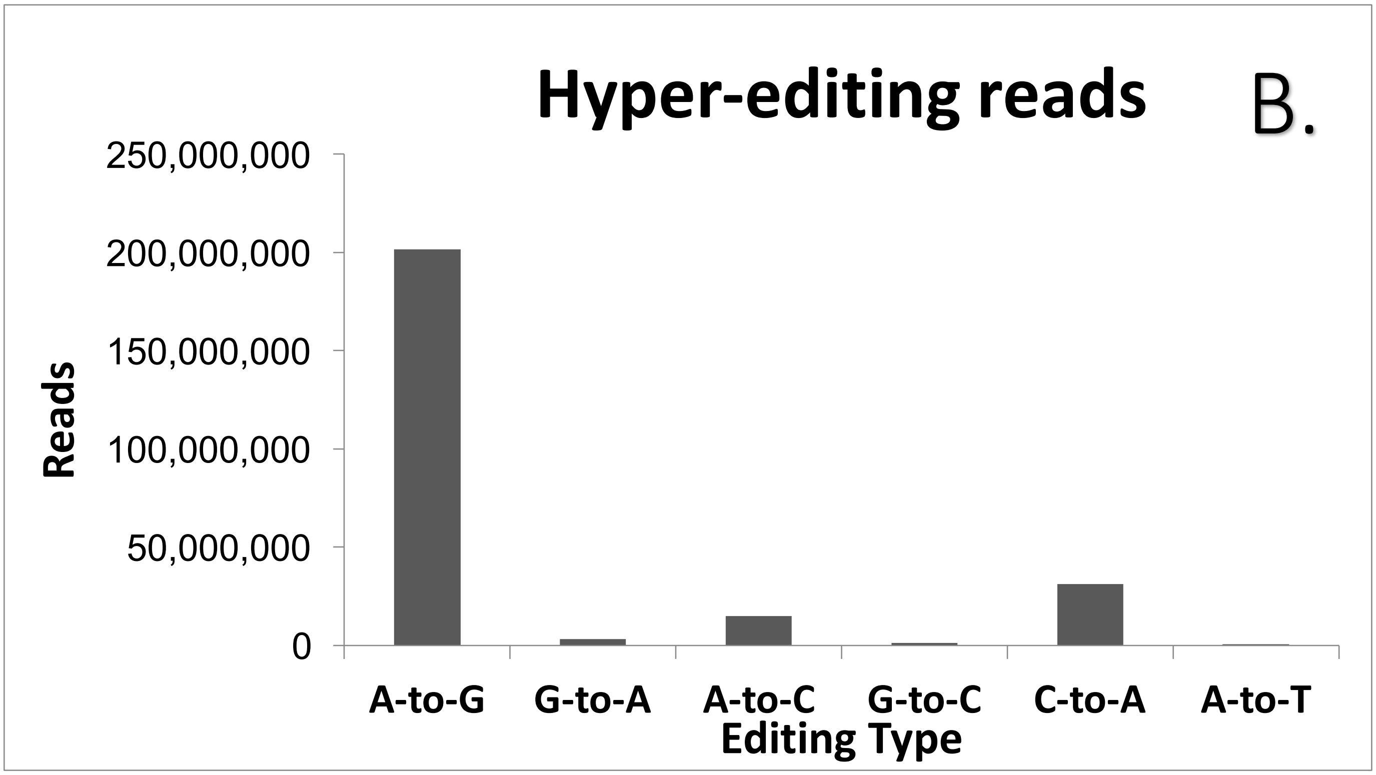
Hyper-editing reads
B.
250,000,000
200,000,000
150,000,000
100,000,000
50,000,000
0
Reads
A-to-G
G-to-A
A-to-C
G-to-C
C-to-A
A-to-T
Editing Type

Supplemental Figure S8. Distribution of hyper-edited reads.

A. Hyper-editing identified in the in-house data. Results showed that 96% of the reads were A-to-G, indicating a high level of specificity for the hyper-editing screen. The 1,613,213 detected A-to-G reads contain 10,666,458 editing events (3,157,685 unique editing-sites). **B.** Hyper-editing identified in the GTEx RNA-Seq data. Results showed that 80% of the reads were A-to-G, indicating a high level of specificity for the hyper-editing screen. The 201,676,069 detected A-to-G reads contain 1,130,591,911 editing events (690,386,562 unique editing-sites).

A.

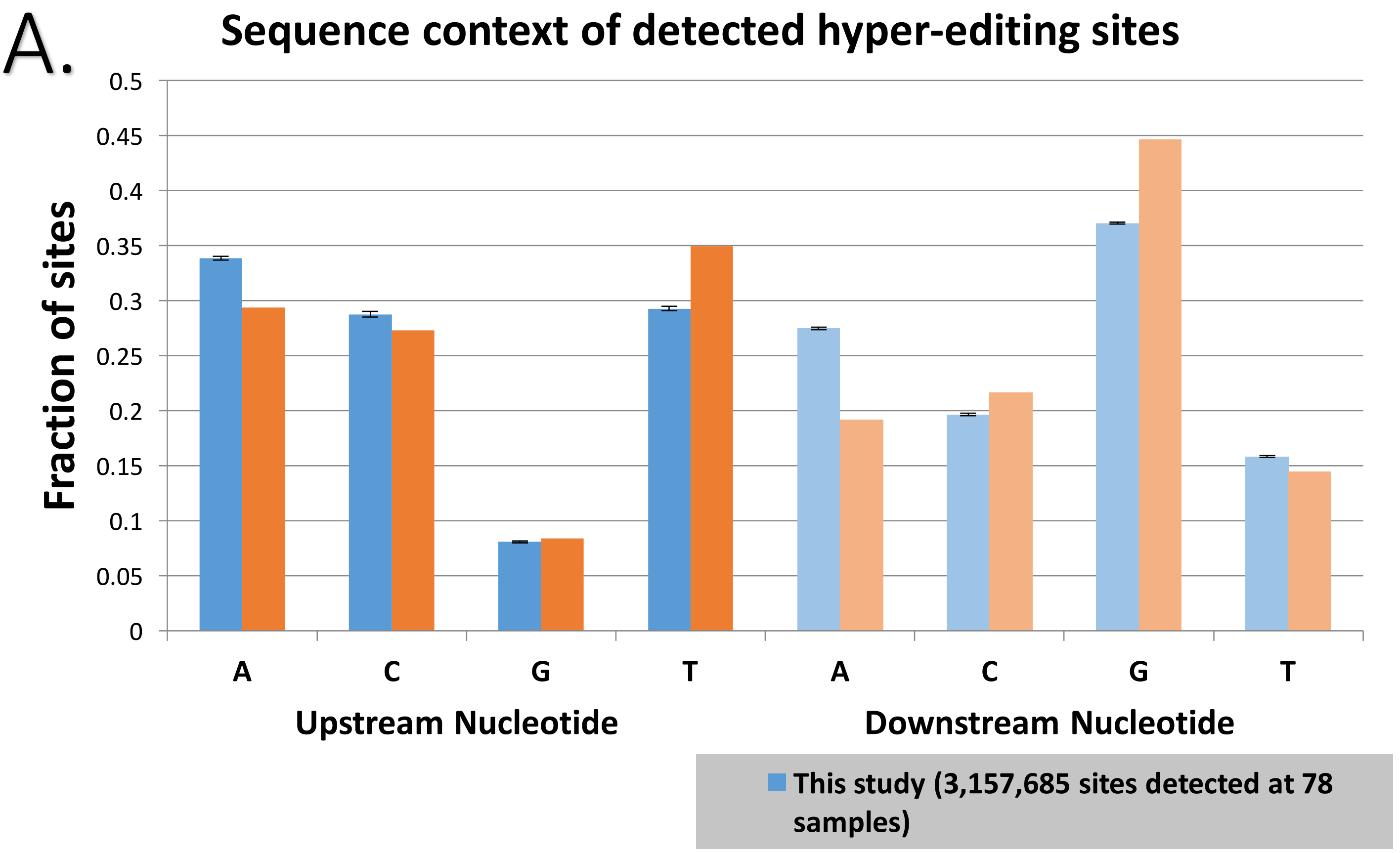

B.

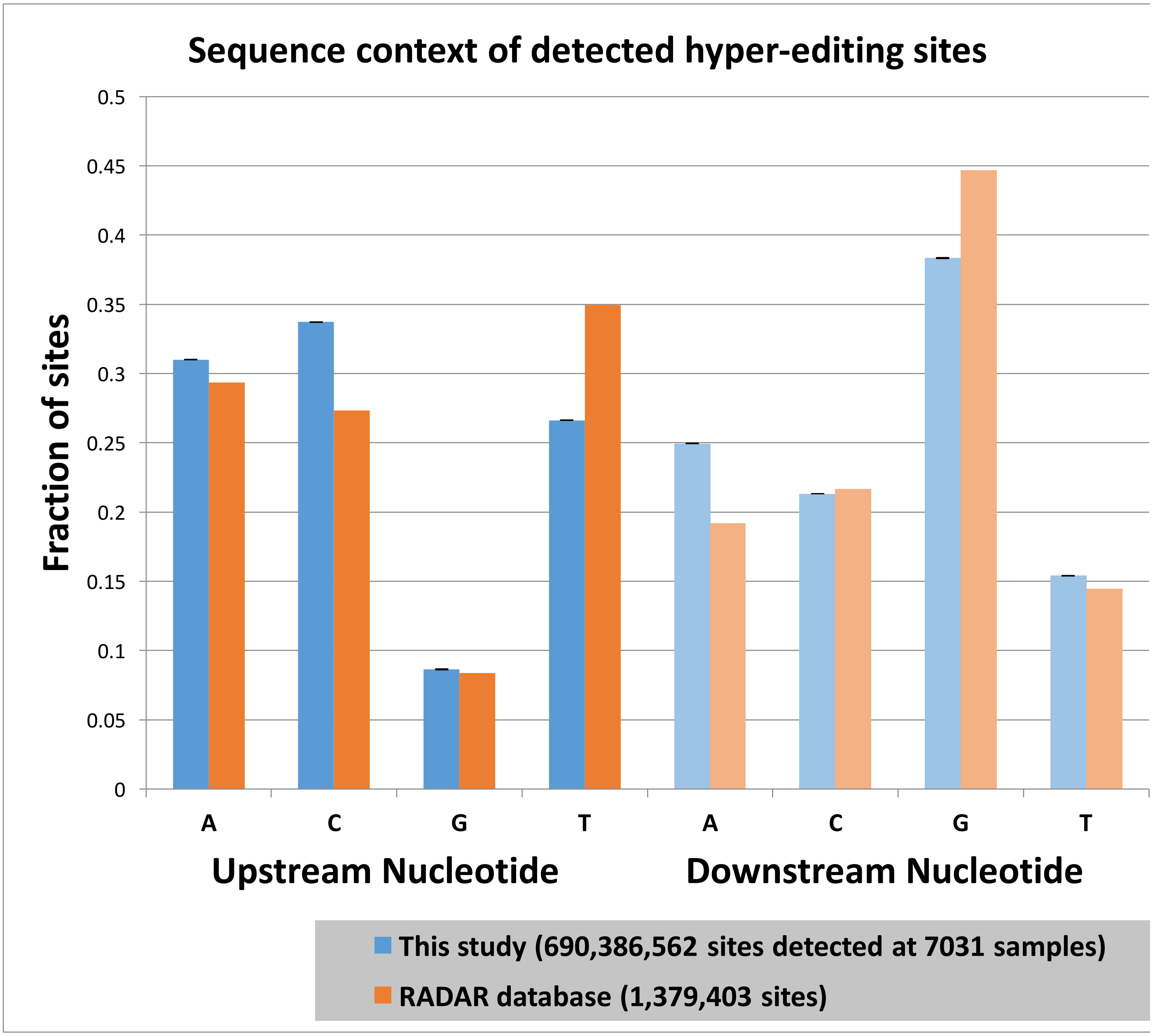
Sequence context of detected hyper-editing sites
Fraction of sites
0.5
0.45
0.4
0.35
0.3
0.25
0.2
0.15
0.1
0.05
0
A
C
G
T
A
C
G
T
Upstream Nucleotide
Downstream Nucleotide
This study (690,386,562 sites detected at 7031 samples)
RADAR database (1,379,403 sites)

Supplemental Figure S9. The sequence context of the Figure S8. The sequence context of the detected hyper-edited A-to-G sites.

The sequence near the detected hyper-editing sites is depleted of Gs upstream and enriched with Gs downstream, in agreement with previously known data about the ADAR motif. The bars correspond to the fraction of editing sites with each type of nucleotide one base upstream and downstream of the site. Results are shown for sites detected in-house RNA-Seq data (A) and GTEx RNA-Seq data (B) using the hyper-editing pipeline and human editing-sites from the RADAR database.

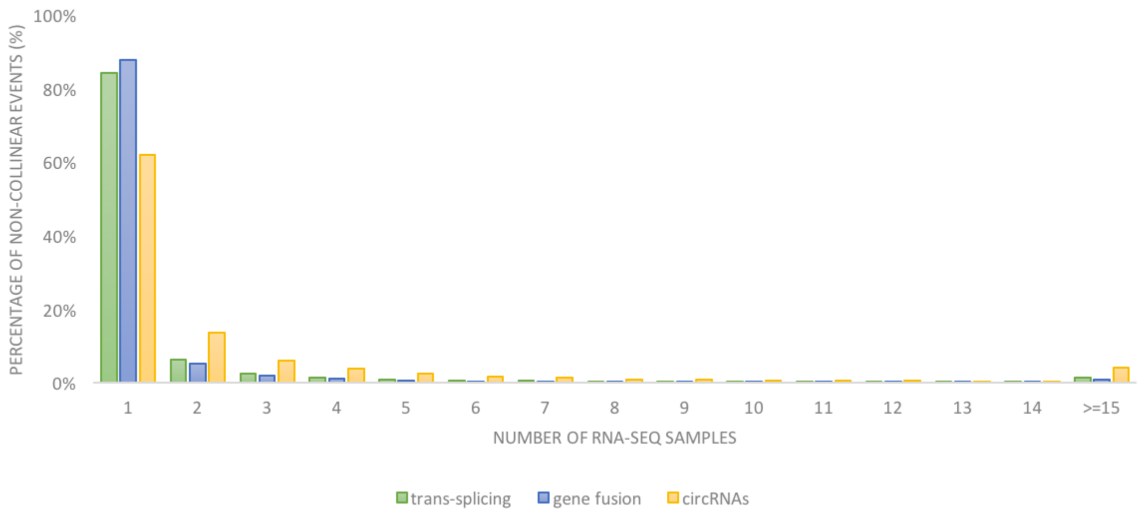

Supplemental Figure S10. Distribution of non-co-linear (NCL) events across 10,641 samples. .

Reads arising from trans-splicing, gene fusion and circRNA events are captured by a TopHat-Fusion and CIRCexplorer2 tools. Trans-splicing events are identified from reads that are spliced distantly on the same chromosome. Gene fusion events are identified from reads spliced across different chromosomes. CircRNAs are identified from reads spliced in a head-to-tail configuration.

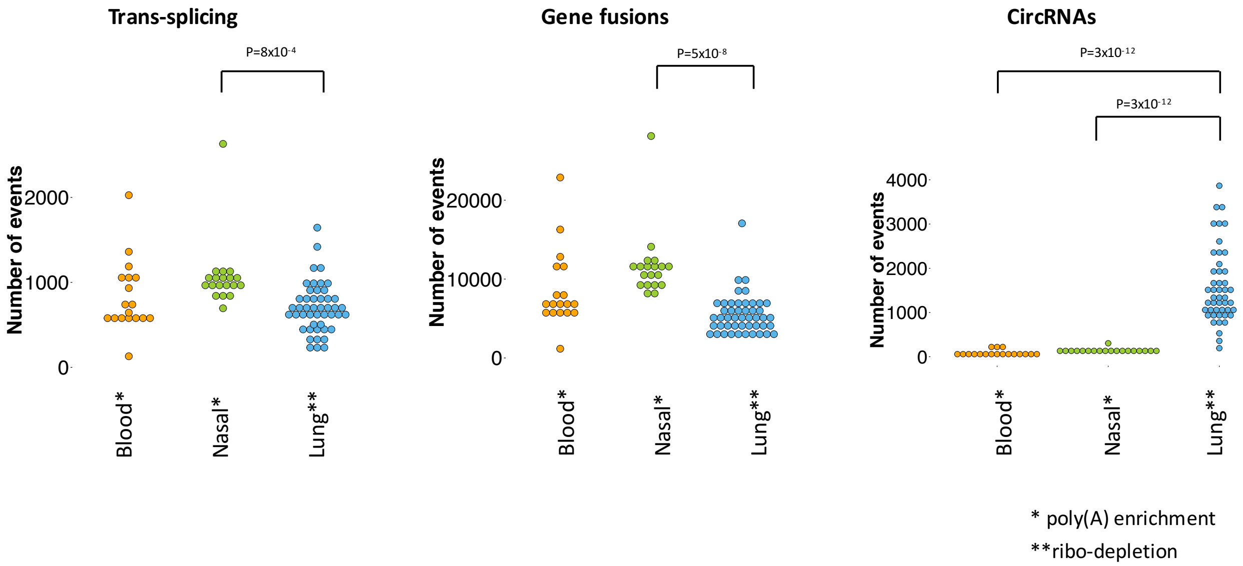

Supplemental Figure S11. Number of NCL events across in-house tissues and library preparation protocols.

NCL events per sample are detected by TopHat-Fusion and CIRCexplorer tools. Samples were prepared with poly(A) selection (whole blood and nasal epithelium) and ribo-depletion (lung epithelium) protocols. Trans-splicing events are identified from reads spliced distantly on the same chromosome. Gene fusion events are identified from reads spliced across different chromosomes.

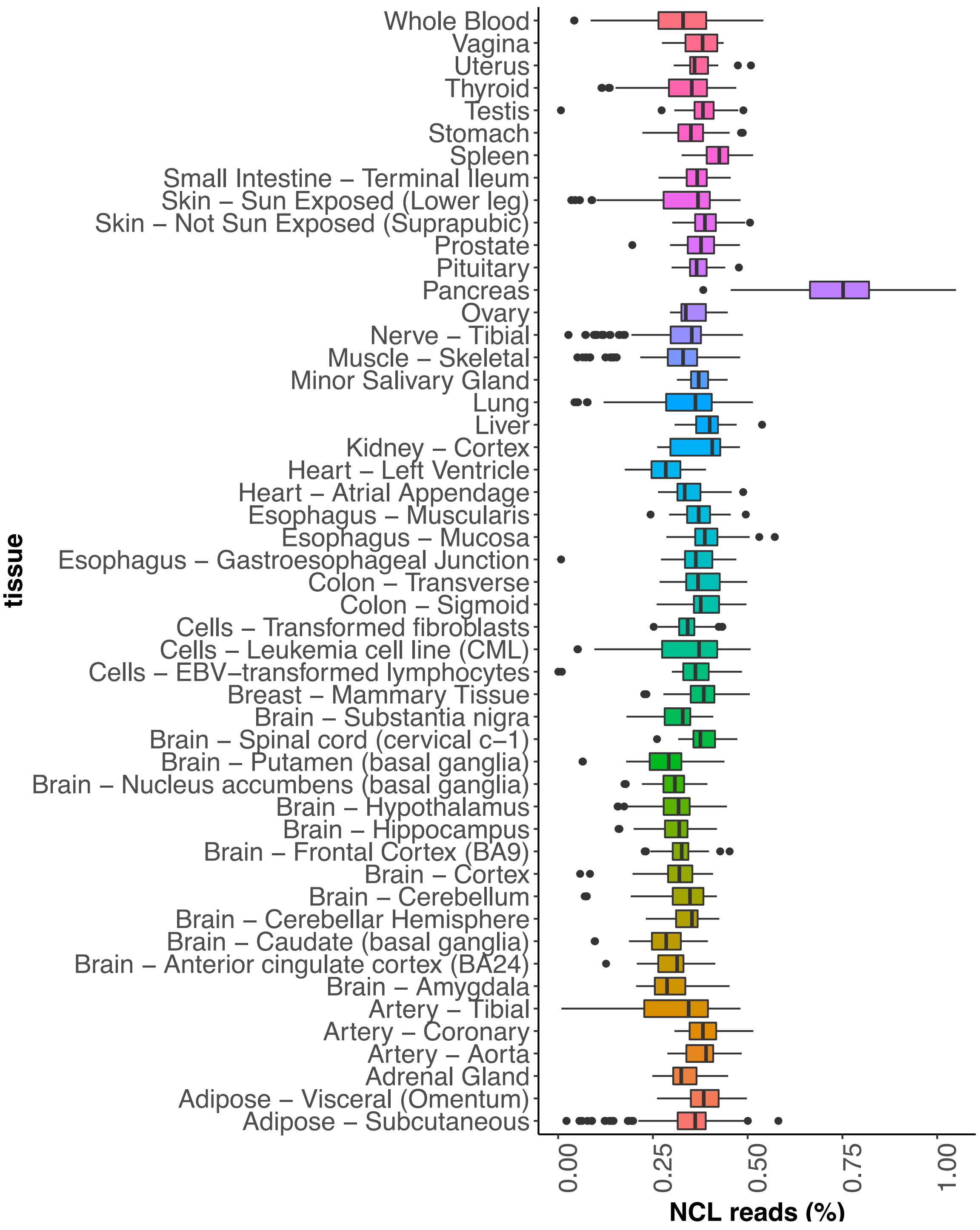
Whole Blood
Vagina
Uterus
Thyroid
Testis
Stomach
Spleen
Small Intestine – Terminal Ileum
Skin – Sun Exposed (Lower leg)
Skin – Not Sun Exposed (Suprapubic)
Prostate
Pituitary
Pancreas
Ovary
Nerve – Tibial
Muscle – Skeletal
Minor Salivary Gland
Lung
Liver
Kidney – Cortex
Heart – Left Ventricle
Heart – Atrial Appendage
Esophagus – Muscularis
Esophagus – Mucosa
Esophagus – Gastroesophageal Junction
Colon – Transverse
Colon – Sigmoid
Cells – Transformed fibroblasts
Cells – Leukemia cell line (CML)
Cells – EBV–transformed lymphocytes
Breast – Mammary Tissue
Brain – Substantia nigra
Brain – Spinal cord (cervical c–1)
Brain – Putamen (basal ganglia)
Brain – Nucleus accumbens (basal ganglia)
Brain – Hypothalamus
Brain – Hippocampus
Brain – Frontal Cortex (BA9)
Brain – Cortex
Brain – Cerebellum
Brain – Cerebellar Hemisphere
Brain – Caudate (basal ganglia)
Brain – Anterior cingulate cortex (BA24)
Brain – Amygdala
Artery – Tibial
Artery – Coronary
Artery – Aorta
Adrenal Gland
Adipose – Visceral (Omentum)
Adipose – Subcutaneous
tissue
0.00
0.25
0.50
0.75
1.00
NCL reads (%)

Supplemental Figure S12. Percentage of NCL reads across GTEx tissues (n=54). Percentages are calculated from the total number of reads. Reads arising from trans-splicing, gene fusion and circRNA events are captured by a TopHat-Fusion and CIRCexplorer2 tools and reported a NCL reads.

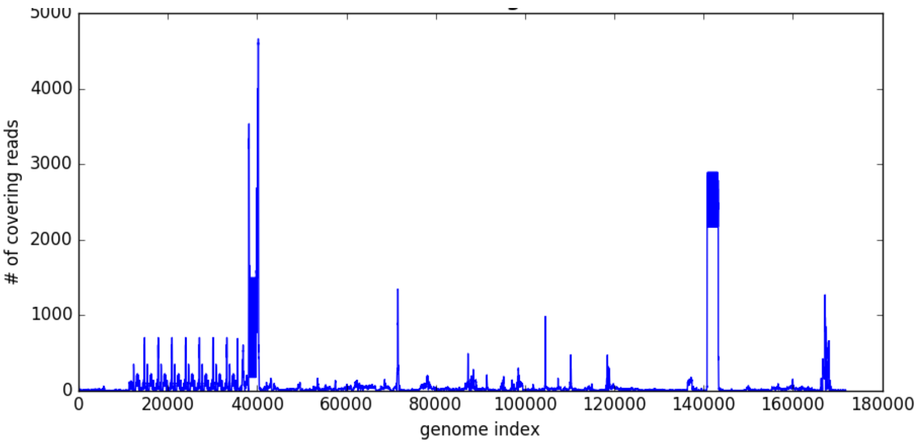

Supplemental Figure S13. An example of coverage plot of EBV virus. Viral reads were obtained by ROP protocol from GTEx RNA-Seq sample of EBV-transformed lymphoblastoid cell lines (LCLs).

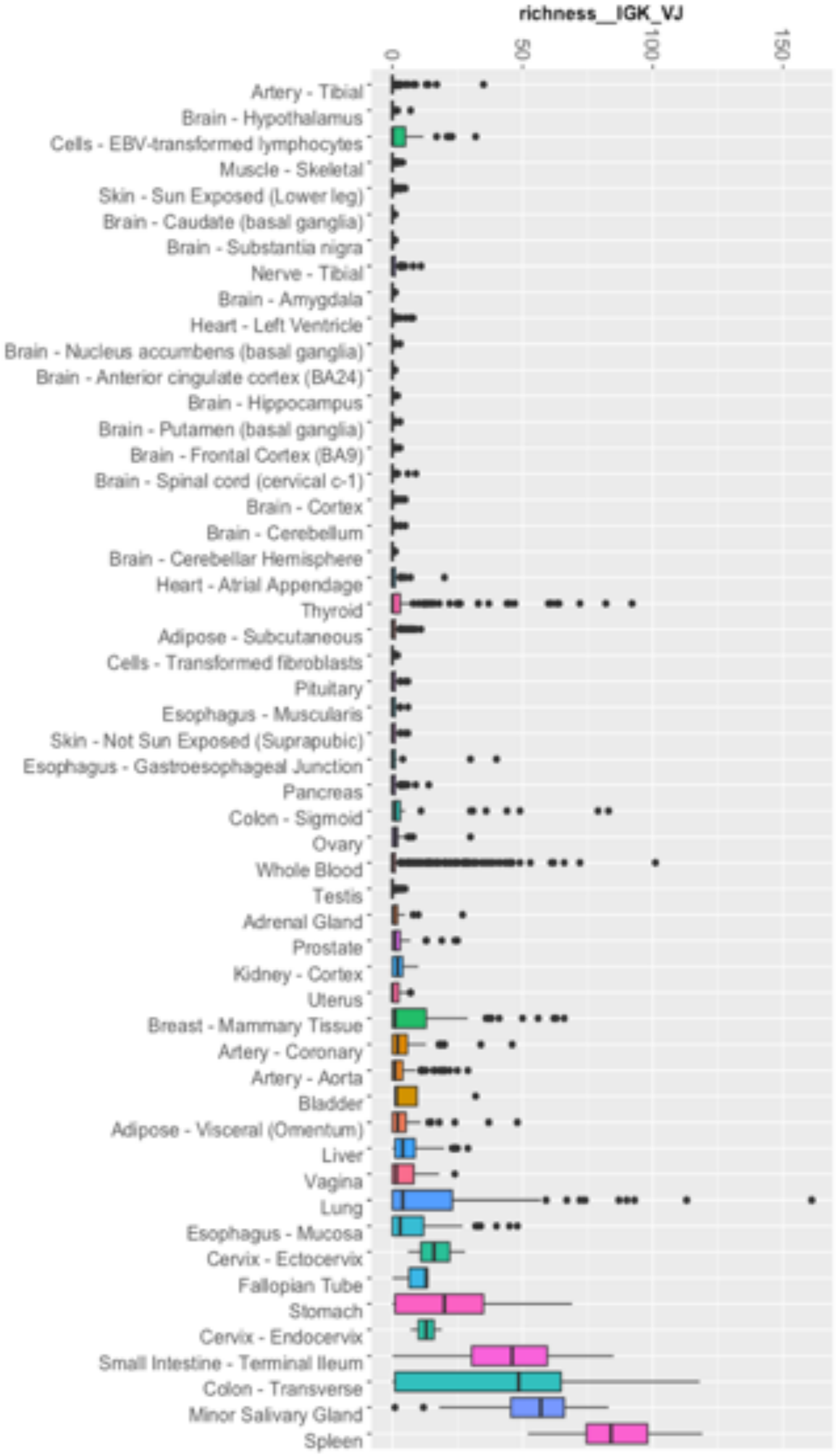
richness__IGK_VJ
0
50
100
150
Artery - Tibial
Brain - Hypothalamus
Cells - EBV-transformed lymphocytes
Muscle - Skeletal
Skin - Sun Exposed (Lower leg)
Brain - Caudate (basal ganglia)
Brain - Substantia nigra
Nerve - Tibial
Brain - Amygdala
Heart - Left Ventricle
Brain - Nucleus accumbens (basal ganglia)
Brain - Anterior cingulate cortex (BA24)
Brain - Hippocampus
Brain - Putamen (basal ganglia)
Brain - Frontal Cortex (BA9)
Brain - Spinal cord (cervical c-1)
Brain - Cortex
Brain - Cerebellum
Brain - Cerebellar Hemisphere
Heart - Atrial Appendage
Thyroid
Adipose - Subcutaneous
Cells - Transformed fibroblasts
Pituitary
Esophagus - Muscularis
Skin - Not Sun Exposed (Suprapubic)
Esophagus - Gastroesophageal Junction
Pancreas
Colon - Sigmoid
Ovary
Whole Blood
Testis
Adrenal Gland
Prostate
Kidney - Cortex
Uterus
Breast - Mammary Tissue
Artery - Coronary
Artery - Aorta
Bladder
Adipose - Visceral (Omentum)
Liver
Vagina
Lung
Esophagus - Mucosa
Cervix - Ectocervix
Fallopian Tube
Stomach
Cervix - Endocervix
Small Intestine - Terminal Ileum
Colon - Transverse
Minor Salivary Gland
Spleen

Supplemental Figure S14. Number of VJ recombinations across GTEx human tissues for IGK chain.

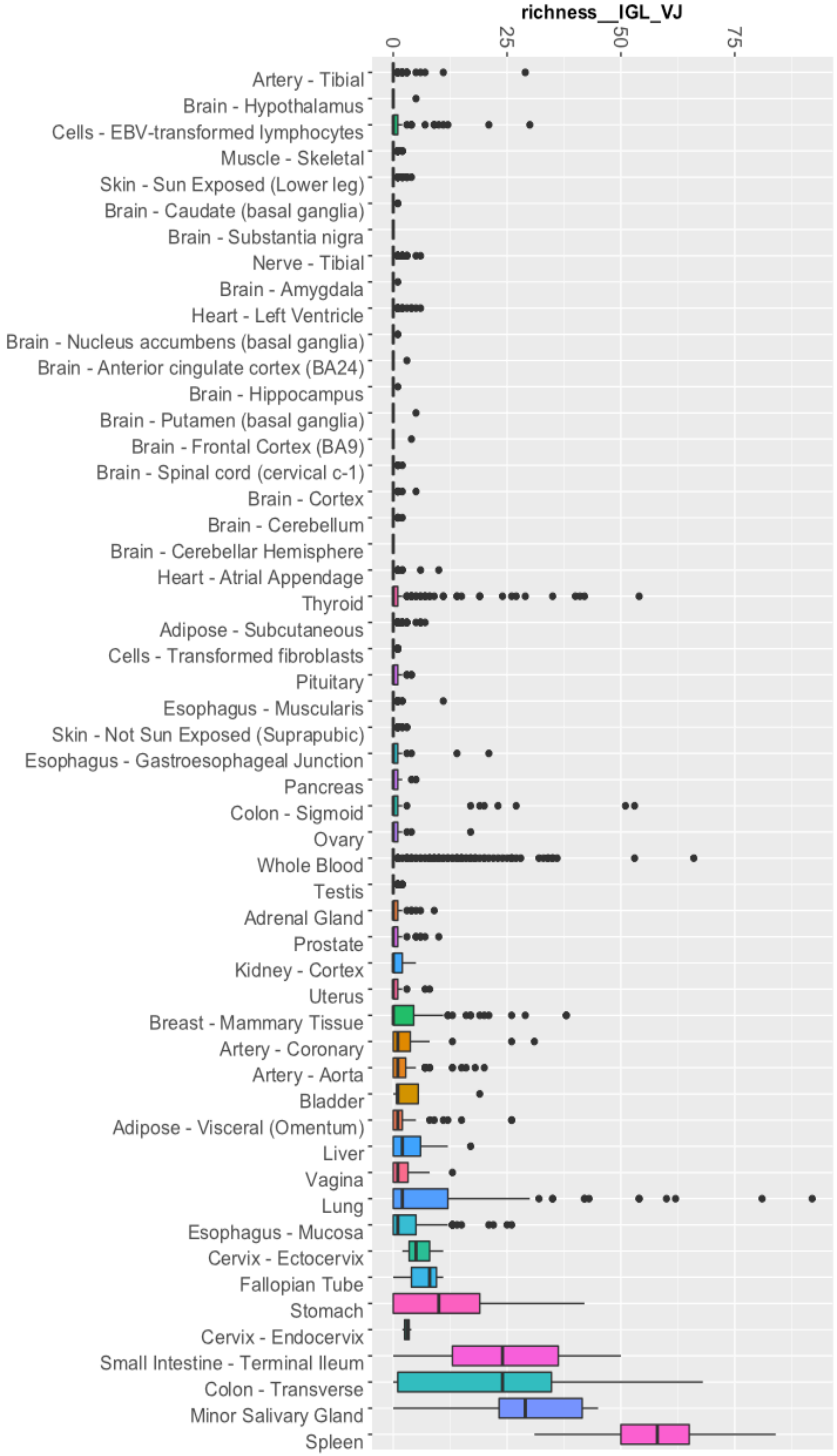

richness__IGL_VJ
0
25
50
75
Artery - Tibial
Brain - Hypothalamus
Cells - EBV-transformed lymphocytes
Muscle - Skeletal
Skin - Sun Exposed (Lower leg)
Brain - Caudate (basal ganglia)
Brain - Substantia nigra
Nerve - Tibial
Brain - Amygdala
Heart - Left Ventricle
Brain - Nucleus accumbens (basal ganglia)
Brain - Anterior cingulate cortex (BA24)
Brain - Hippocampus
Brain - Putamen (basal ganglia)
Brain - Frontal Cortex (BA9)
Brain - Spinal cord (cervical c-1)
Brain - Cortex
Brain - Cerebellum
Brain - Cerebellar Hemisphere
Heart - Atrial Appendage
Thyroid
Adipose - Subcutaneous
Cells - Transformed fibroblasts
Pituitary
Esophagus - Muscularis
Skin - Not Sun Exposed (Suprapubic)
Esophagus - Gastroesophageal Junction
Pancreas
Colon - Sigmoid
Ovary
Whole Blood
Testis
Adrenal Gland
Prostate
Kidney - Cortex
Uterus
Breast - Mammary Tissue
Artery - Coronary
Artery - Aorta
Bladder
Adipose - Visceral (Omentum)
Liver
Vagina
Lung
Esophagus - Mucosa
Cervix - Ectocervix
Fallopian Tube
Stomach
Cervix - Endocervix
Small Intestine - Terminal Ileum
Colon - Transverse
Minor Salivary Gland
Spleen

150

Supplemental Figure S15. Number of VJ recombinations across GTEx human tissues for IGL chain.

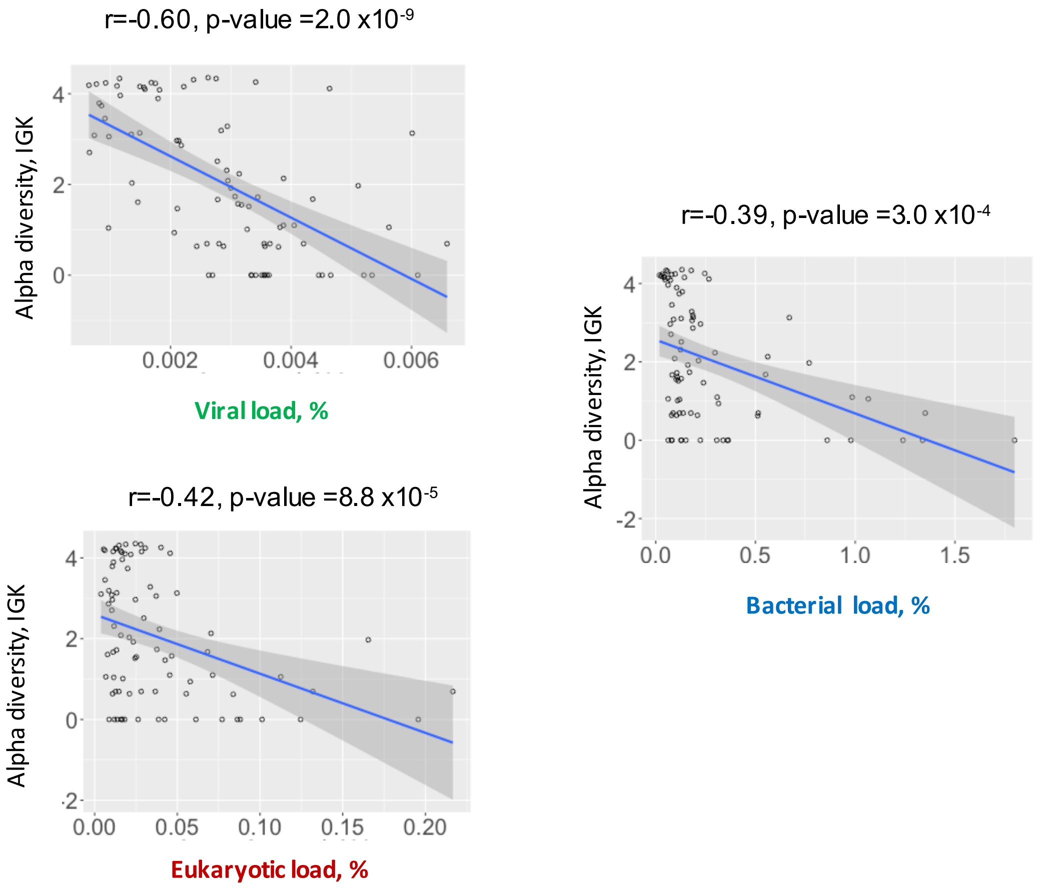

Supplemental Figure S16. Association between microbial load and immune diversity.

(a) Scatterplot of the viral load and combinatorial immune diversity of IGK locus. Pearson correlation coefficient (r) and p -value are reported. (b) Scatterplot of the eukaryotic load and combinatorial immune diversity of IGK locus. Pearson correlation coefficient (r) and p -value are reported. (c) Scatterplot of the bacterial load and combinatorial immune diversity of IGK locus. Pearson correlation coefficient (r) and p -value are reported.

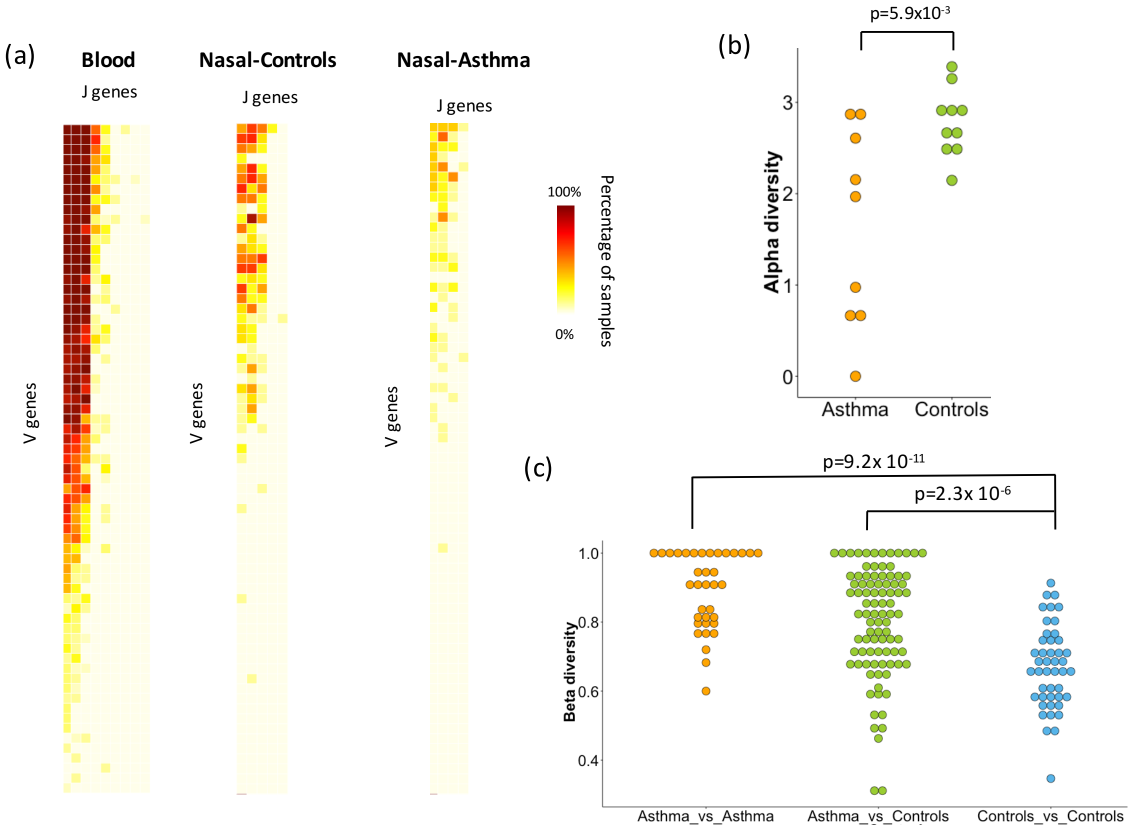

Supplemental Figure S17. Combinatorial diversity of immunoglobulin lambda locus (IGL) locus differentiates disease status.

(a) Heat map depicting the percentage of RNA-Seq samples supporting particular VJ combination for whole blood, nasal epithelium of healthy controls and asthmatic individuals. Each row corresponds to a V gene and each column corresponds to a J gene. (b) Alpha diversity is measured using the Shannon entropy incorporating the total number of VJ combinations and their relative proportions. Nasal epithelium of asthmatic individuals exhibits decreased combinatorial diversity of IGK locus compared to that of healthy controls (p-value=5.9×10^{-3}). (c) Compositional similarities between the samples in terms of gain or loss of VJ combinations of IGK locus are measured using the Sørensen–

Dice index across pairs of samples from the same group (Asthma, Controls) and pairs of sample from different groups (Asthma versus Controls). Lower level of similarity is observed between nasal samples of the asthmatic individuals compared to the unaffected controls (p-value<9.2×10^{-11}). Nasal samples of the unaffected controls are more similar to each other than to the asthmatic individuals (p-value<2.3×10^{-6}).

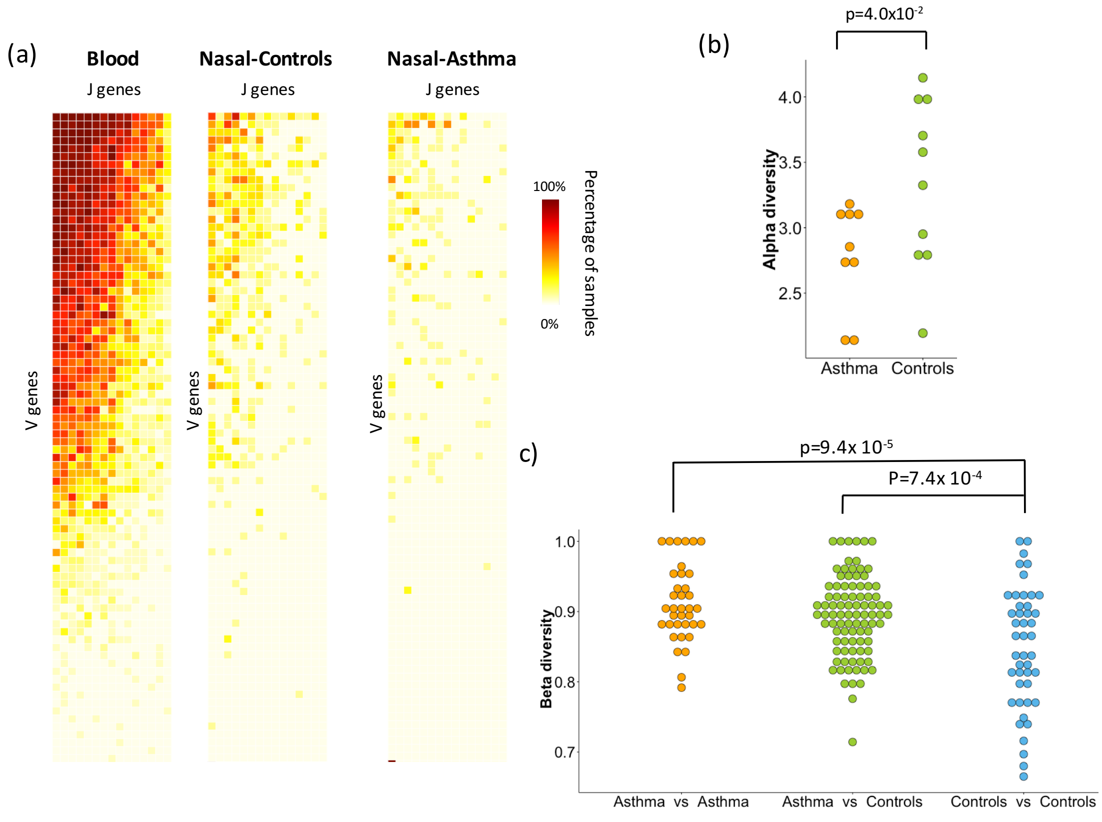

Supplemental Figure S18. Combinatorial diversity of T cell receptor beta (TCRB) locus differentiates disease status.

(a) Heat map depicting the percentage of RNA-Seq samples supporting of particular VJ combination for whole blood, nasal epithelium of healthy controls and of asthmatic individuals. Each row corresponds to a V gene and each column corresponds to a J gene. (b) Alpha diversity is measured using the Shannon entropy incorporating the total number of VJ combinations and their relative proportions. The nasal epithelium of asthmatic individuals exhibits a decrease in combinatorial diversity of IGK locus compared to that of healthy controls (p-value = 4.0×10^{-2}). (c) Compositional similarities between the samples in terms of gain or loss of VJ combinations of IGK locus are measured using the Sørensen–Dice index across pairs of sample from the same group (Asthma, Controls) and pairs of sample from different groups (Asthma versus Controls). Lower level of similarity is observed between nasal samples of asthmatic individuals compared to unaffected controls (p-value < 9.4×10^{-5}). Nasal samples of unaffected controls are more similar to each other than to the asthmatic individuals (p-value < 7.4×10^{-4}).

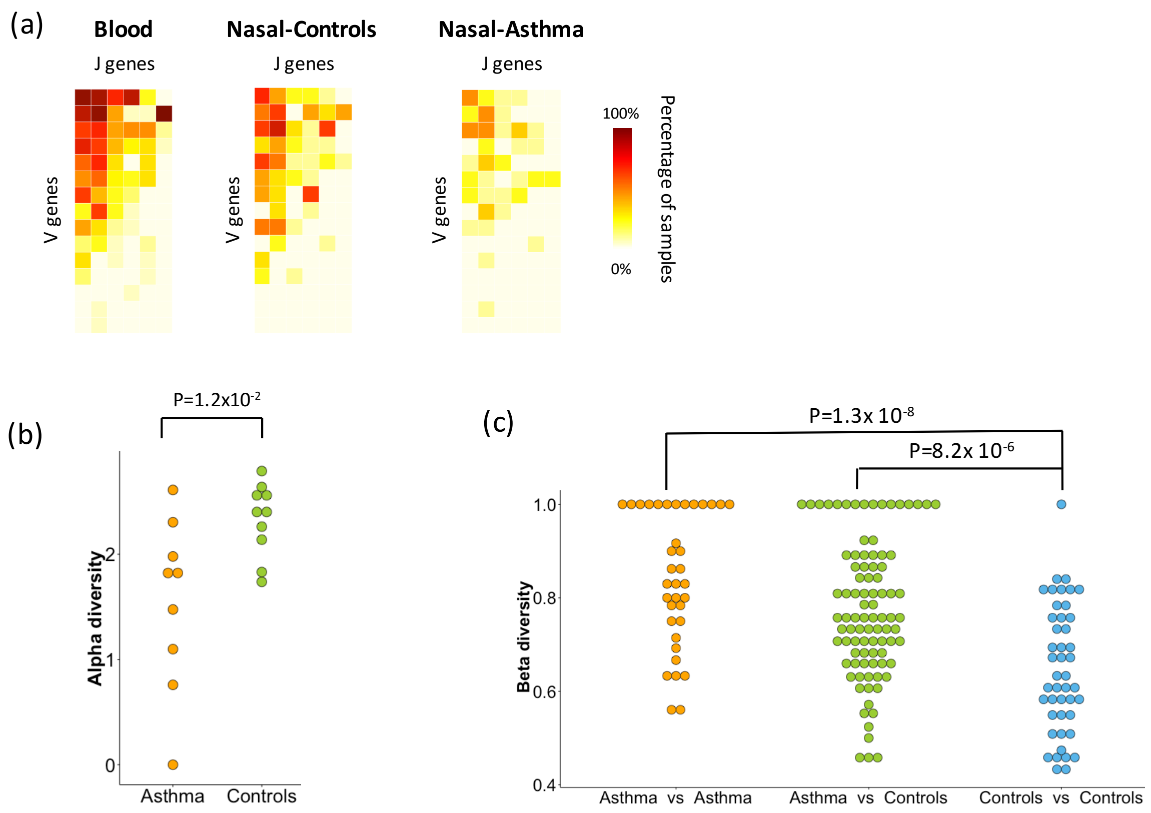

Supplemental Figure S19. Combinatorial diversity of T cell receptor gamma (TCRG) locus differentiates disease status.

(a) Heat map depicting the percentage of RNA-Seq samples supporting of a particular VJ combination for whole blood, nasal epithelium of healthy controls and asthmatic individuals. Each row corresponds to a V gene and each column corresponds to a J gene. (b) Alpha diversity is measured using the Shannon entropy incorporating the total number of VJ combinations and their relative proportions. Nasal epithelium of asthmatic individuals exhibits decreased combinatorial diversity of IGK locus compared to that of healthy controls (p-value = 1.2 x 10^{-2}, ANOVA). (c) Compositional similarities between the samples in terms of gain or loss of VJ combinations of IGK locus are measured using the

Sørensen–Dice index across pairs of sample from the same group (Asthma, Controls) and pairs of sample from different groups (Asthma versus Controls). Lower level of similarity is observed between nasal samples of asthmatic individuals compared to unaffected controls (p-value < 1.3×10^{-8}). Nasal samples of unaffected controls are more similar to each other than to the asthmatic individuals (p-value < 8.2×10^{-6}).

(a) Number of reads categorized by RNA-Seq aligners

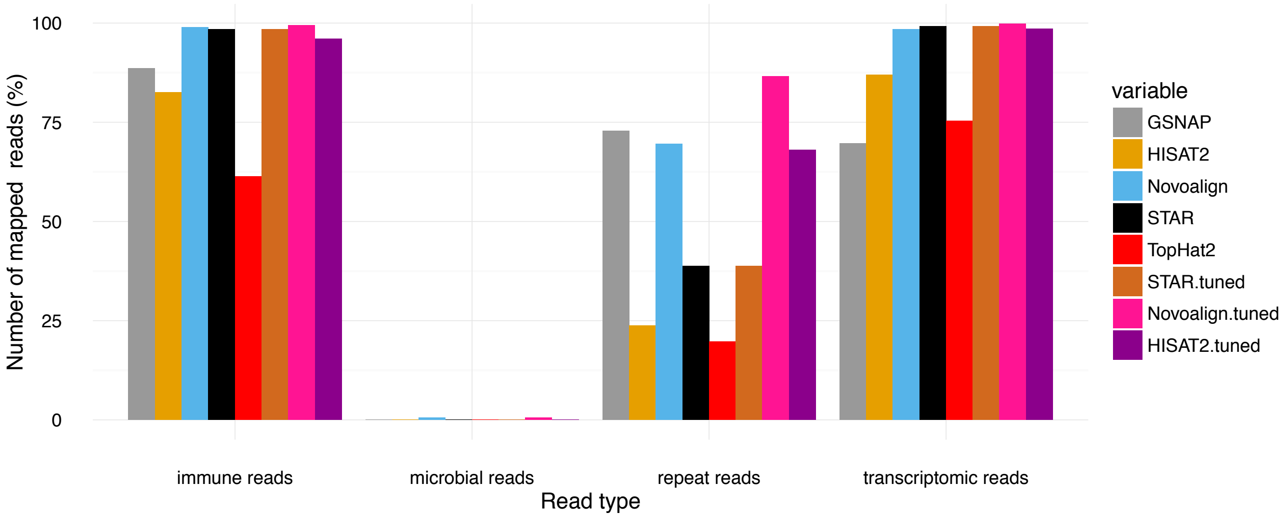

(b) Number of reads categorized by ROP using different RNA-Seq aligners

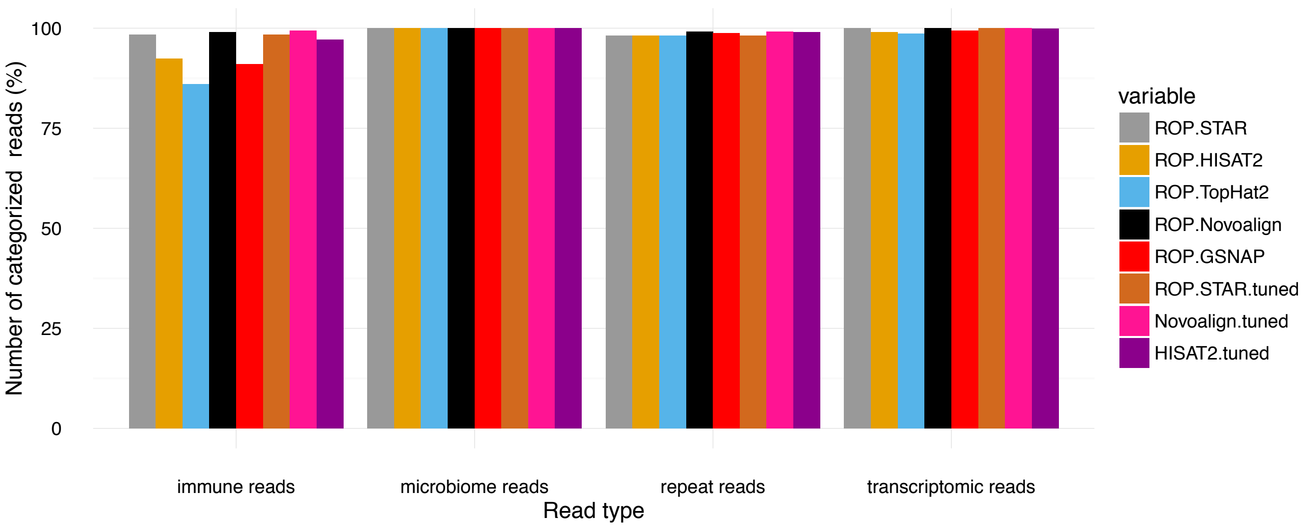

Supplemental Figure S20. The effect of RNA-Seq aligner on the fraction of reads accounted by ROP. Percentages are calculated from the total number of reads in each category. RNA-Seq aligners were run with default and optimized (tuned) parameters. We use tuned setting recommended by Baruzzo et al. (2017). TopHat2 and GSNAP were only run with default settings. Results are presented for simulated RNA-Seq data composed of transcriptomic, repeat, immune, and microbial reads. (a) Percentages of reads accounted by RNA-Seq aligners. (b) Percentages of reads categorized by ROP across five state of the art aligners.

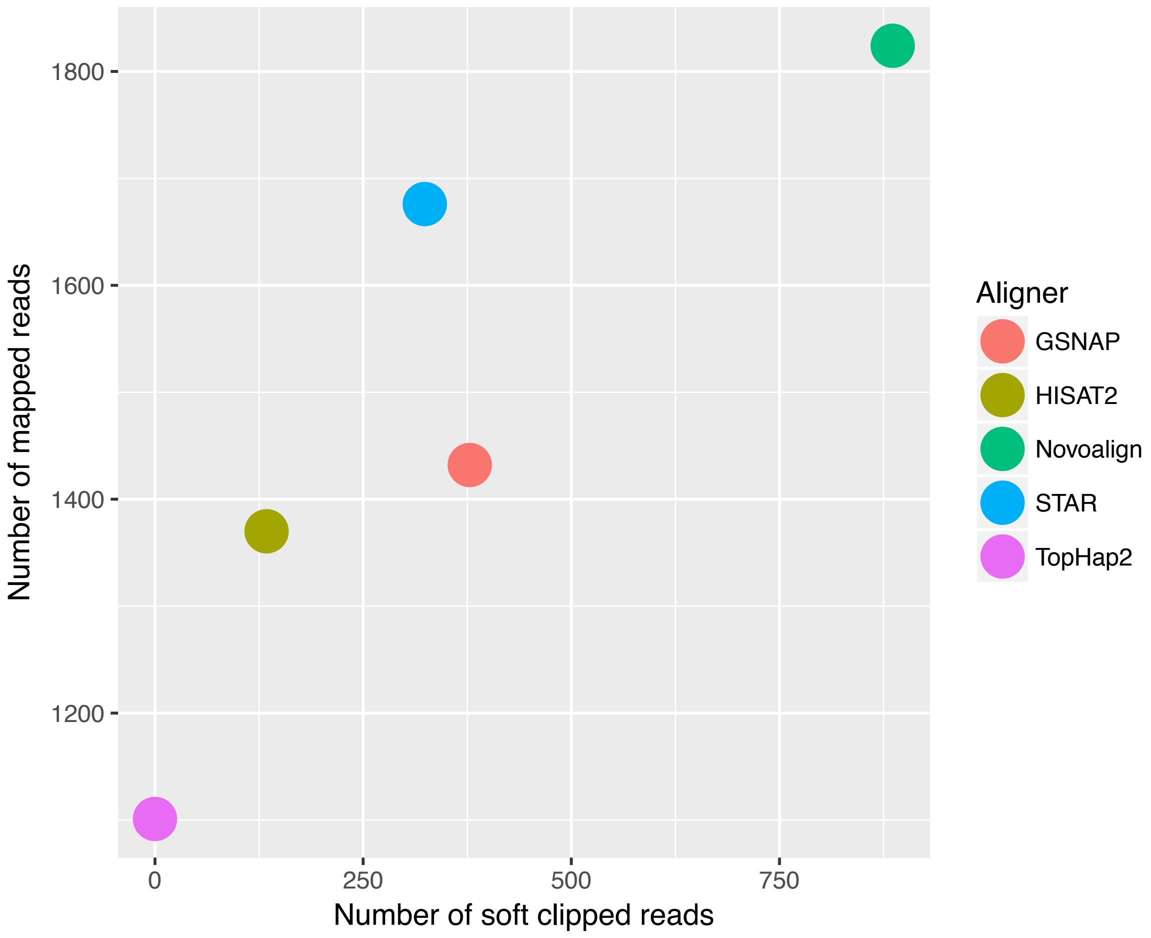

Supplemental Figure S21. Relationship between the number of soft clipped RNA-Seq reads (partially mapped reads) and the total number of reads. Results are presented for simulated RNA-Seq data composed of transcriptomic, repeat, immune, and microbial reads.

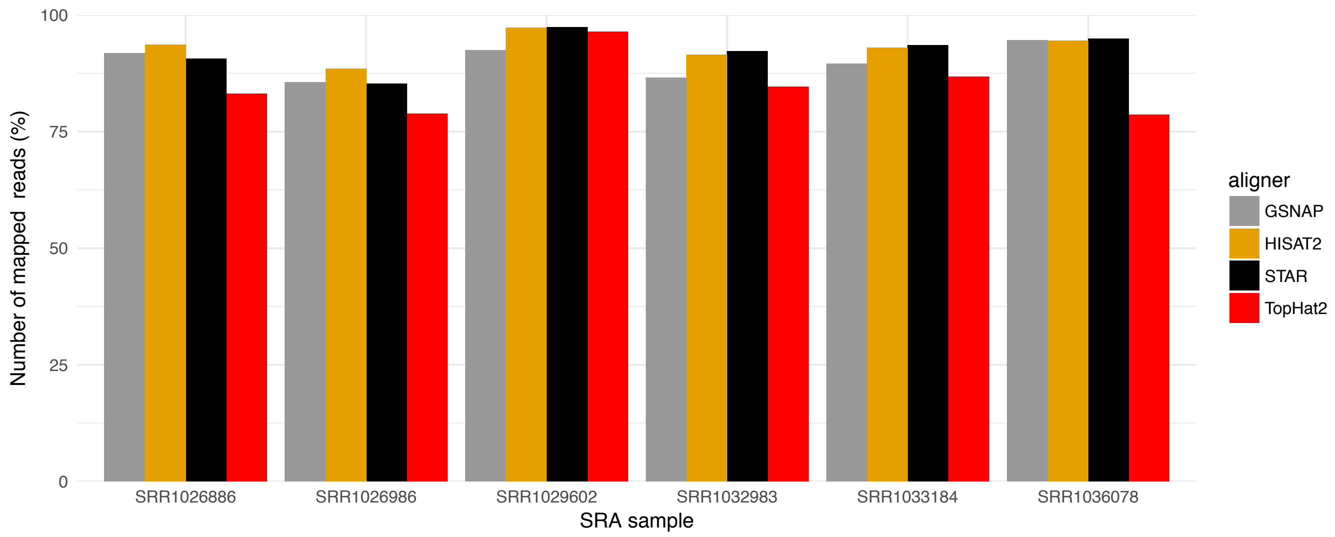

Supplemental Figure S22. Number of the RNA-Seq reads mapped to the human reference genome across five state-of-the-art RNA-Seq aligners. Number of mapped reads is separately reported for each SRA sample. Percentages are calculated from the total number of reads. Results are presented for 10 randomly selected SRA RNA-Seq samples. Tools were run with default parameters. Novoalign was excluded from this analysis because none of the experiments finished running within 24 hours.
